# The Inhibitory Potential of Ferulic Acid Derivatives against the SARS-CoV-2 Main Protease: Molecular Docking, Molecular Dynamics, and ADMET Evaluation

**DOI:** 10.3390/biomedicines10081787

**Published:** 2022-07-25

**Authors:** Io Antonopoulou, Eleftheria Sapountzaki, Ulrika Rova, Paul Christakopoulos

**Affiliations:** Biochemical Process Engineering, Division of Chemical Engineering, Department of Civil, Environmental and Natural Resources Engineering, Luleå University of Technology, SE-97187 Luleå, Sweden; eleftheria.sapountzaki@ltu.se (E.S.); ulrika.rova@ltu.se (U.R.); paul.christakopoulos@ltu.se (P.C.)

**Keywords:** SARS-CoV-2, M^pro^, enzyme inhibition, ferulic acid, molecular docking, molecular dynamics, ADMET

## Abstract

The main protease (M^pro^) of SARS-CoV-2 is an appealing target for the development of antiviral compounds, due to its critical role in the viral life cycle and its high conservation among different coronaviruses and the continuously emerging mutants of SARS-CoV-2. Ferulic acid (FA) is a phytochemical with several health benefits that is abundant in plant biomass and has been used as a basis for the enzymatic or chemical synthesis of derivatives with improved properties, including antiviral activity against a range of viruses. This study tested 54 reported FA derivatives for their inhibitory potential against M^pro^ by in silico simulations. Molecular docking was performed using Autodock Vina, resulting in comparable or better binding affinities for 14 compounds compared to the known inhibitors N3 and GC376. ADMET analysis showed limited bioavailability but significantly improved the solubility for the enzymatically synthesized hits while better bioavailability and druglikeness properties but higher toxicity were observed for the chemically synthesized ones. MD simulations confirmed the stability of the complexes of the most promising compounds with M^pro^, highlighting FA rutinoside and compound e27 as the best candidates from each derivative category.

## 1. Introduction

During the past two years, public health and socioeconomic life have been going through a severe crisis due to the Coronavirus disease 2019 (COVID-19) pandemic caused by severe acute respiratory syndrome Coronavirus-2 (SARS-CoV-2), which has led to more than 450 million cases and more than 6 million deaths worldwide [1]. SARS-CoV-2 belongs to the family of coronaviruses, which have posed a threat to public health in the past, with the severe acute respiratory syndrome-coronavirus (SARS-CoV) and Middle East respiratory syndrome (MERS-CoV) outbreaks in 2002 and 2012, respectively [2].

The development of vaccines was remarkably quick, allowing many countries to initiate the vaccination process in the beginning of 2021 and, therefore, provide a valuable weapon to boost immunity against the virus [3]. As far as drugs and other non-vaccine therapeutic options are concerned, remdesivir, an RNA-dependent RNA polymerase inhibitor, is the only one to receive FDA approval for use in COVID-19 patients [4]. Currently, 15 other products have received emergency use authorizations by the FDA, including the protease inhibitor Paxlovid [5].

However, new variants continue to emerge, affecting the transmissibility of the virus, the impact of the disease, and the immunity against it. Currently, variants beta, gamma, delta, and omicron are labeled as variants of concern (VOCs) by WHO [6]. Among them, the omicron variant is the most capable of antigenic escape, causing concern over the efficacy of current therapeutic routes, such as vaccines and monoclonal antibody treatments [7,8]. Thus, it is of great importance to develop tools that can remain effective against potential viral mutations.

Therapeutic targets to combat COVID-19 include structural and functional proteins of the virus, and virulence factors and host proteins that are useful for viral proliferation. Among them, the focus of this work is the SARS-CoV-2 main protease (M^pro^, 3CL^pro^, or Nsp5). M^pro^ is a very promising antiviral target, as it plays a major role in the viral life cycle, while there is also adequate information available on its structure and mechanism to allow further investigation, both in silico and in vitro [9]. Inhibition of M^pro^ is expected to exhibit high specificity and limited side effects, as there are no other human enzymes that recognize the same sequence and the peptide bond as a cleavage site [10]. In addition, M^pro^ seems to have fewer mutation hotspots compared to other targets, such as the spike protein [11]. More specifically, the main protease of the SARS-CoV-2 omicron and delta variants are almost identical to that of the wild type, with only one prevalent mutation observed in the case of omicron (P132H) and none in the case of delta [12,13]. Moreover, the protease exhibits high conservation among coronaviruses (e.g., 96% sequence identity between SARS-CoV-2 and SARS-CoV main protease), meaning that its inhibitors are very likely to be effective against variants or other viruses of the same family [14,15].

Taking into consideration all the above, and the fact that there are numerous natural compounds that are being screened for their antiviral properties and have exhibited efficacy in fighting a wide range of viruses, including SARS-CoV-2, plants emerge as a potential valuable source of bioactive compounds, which could be utilized as nutraceuticals to contribute to the protection against a viral infection and potentially aid immunity [16,17,18].

Ferulic acid (FA, 4-hydroxy-3-methoxycinnamic acid) is a phenolic bioactive compound belonging to a group of hydroxycinnamic acids that have drawn attention as nutraceuticals due to their numerous beneficial properties, such as high antioxidant activity, anti-inflammatory, antibacterial, neuro- and photoprotective, anticancer, antidiabetic, and skin-whitening effects [19,20,21]. It has also been identified as an antiviral agent against several viruses [22], including SARS-CoV-2 [23]. The profile of FA as a promising nutraceutical is reinforced by its low toxicity; however, its bioavailability is limited and needs to be improved [24]. Although further investigation of its pharmacokinetic properties is required, its absorption and metabolism appear to be dose and form dependent, indicating that further investigation of FA derivatives could lead to compounds with improved pharmacokinetic properties [25,26,27].

FA is abundantly present in vegetables, fruits, cereals, flowers, leaves, beans, coffee seeds, and nuts [28,29]. It can be found in monocots (rice, wheat, etc.) and dicots, (e.g., sugar beet pulp, spinach, glasswort, carrot) [30] and is the most commonly found hydroxycinnamic acid in plant cell walls, where it is esterified to polysaccharides. In monocots, it is bound to xylan at the *O*-5 of its-l-arabinose moieties while in dicots, it is often bound to the neutral side chains of pectin, esterified to the *O*-2 of a-l-arabinose or to the *O*-6 of b-d-galactose units. It also forms dimers and trimers, which create crosslinks between polysaccharide chains and lignin, contributing to the rigidity of the lignocellulosic matrix of plant cell walls. Overall, it is observed mainly in its *trans*- isomeric form and esterified with mono- and disaccharides, glycoproteins, polyamines, hydroxylated fatty acids, alcohols, and flavonoids, apart from plant cell wall polysaccharides [28,29,31]. Various studies have been carried out on possible routes for the derivatization of FA towards compounds with preferable bioactive and pharmacokinetic properties [32,33,34,35,36]. Such derivatives can be synthesized chemically or enzymatically, and many of them have exhibited promising antiviral properties [22].

This work focuses on the evaluation of a broad range of FA derivatives for their ability to bind to and, therefore, inhibit M^pro^. The goal is to determine which compounds are more likely to form a stable complex with M^pro^ and to provide further information on the effect of derivatization on the inhibitory efficacy of the compounds, and their pharmacokinetic properties. By examining enzymatically and chemically synthesized derivatives with varying structural characteristics, additional insight is gained into the potential of the different routes for the development of antiviral nutraceuticals.

## 2. Materials and Methods

### 2.1. Ligand and Receptor Preparation for Docking Simulations

The phytochemicals selected were FA derivatives reported in the literature. Their structures were adopted from the original publications and then constructed in Chemsketch (ACD/Labs, Toronto, ON, Canada). The docking simulation essentially calculates the lowest possible energy and the conformation that leads to it of the complex between a larger macromolecule (receptor), in this case SARS-CoV-2 M^pro^, and a smaller molecule (ligand), which in this case is a respective inhibitor or FA derivative. For the docking to be executed, the receptor and the ligand need to be defined and prepared. The 3D structure of each ligand, drawn in Chemsketch, was optimized through the same program, and saved in a .mol file, before being imported and energy minimized in YASARA Version 20.12.2 [37]. The protease (M^pro^) structure used for the simulation is available in the Protein Data Bank (PDB) (https://www.rcsb.org/ accessed on 1 May 2022) under the PDB ID 6LU7, with a resolution of 2.16 Å. This structure was selected among the numerous structures deposited in the PDB because it was the first one to be made available and the one that is more often used in studies featuring molecular docking simulations. The ligand that is bound to the protease in this particular co-crystallization structure was deleted, the receptor was cleaned, and its hydrogen network was optimized. A simulation cell was built as a cube centered in the atoms of the catalytic dyad His41 and Cys145 and extended as many Å as needed for its side to be 2–3 Å longer than the length of each ligand. This margin allowed for flexibility as the ligand may acquire different conformations onto the active site; however, it was not so large that it increased the uncertainty of the docking simulation.

### 2.2. Molecular Docking Simulation and Data Output

Molecular docking was performed using the embedded macro in YASARA, AutoDock Vina, using the default parameters. The program calculates the binding energy of the possible receptor–ligand complexes taking into consideration steric, hydrophobic, and hydrogen-bonding interactions [38]. During the simulation, the program performs 25 docking runs, which produce 25 possible ligand–receptor-binding conformations. The different conformations that are arranged around the same hotspot and have an RMSD smaller than 5 Å from each other form a cluster. The number of clusters differs depending on the simulation, and the binding energy of the most favorable conformation within the cluster is reported.

After each simulation, the program generated a report documenting the output data for each run and cluster. This includes the binding energy (in kcal/mol), the dissociation constant (in pM), and the contacting residues for each case (including hydrogen bond, hydrophobic, pi-pi, cation–pi, and ionic interactions). The binding energy is calculated by subtracting the energy of the ligand–receptor complex in the bound state from the energy when the ligand is at an infinite distance from the receptor and is given as output from the software as a positive number. Based on the used simulation, a higher binding energy indicates a higher binding affinity. Reference to other works attributes the binding energies as negative values; therefore, in such cases, the lowest binding energies are regarded as the ones resulting in a higher binding affinity. The software manual states that the only difference between the positive binding energy values reported and the negative energies of binding reported in other works is the flipped sign. Therefore, we report the binding energies as negative values to be in accordance with the relevant literature.

### 2.3. Validation of the Docking Method

Apart from FA and its derivatives, two known M^pro^ inhibitors with available co-crystallization structures in complex with the protease deposited at the PDB were docked to M^pro^. The docked complex was compared with the PDB co-crystallization complex in order to confirm the reliability of molecular docking and the selected software in particular as a tool to accurately simulate the targeted protein–ligand interactions. The selected inhibitors were N3 (PDB ID: 6LU7) and GC376 (PDB ID: 7D1M). The first cluster given as a simulation output was superposed to the co-crystallization structure and the reproducibility of the binding mode of the ligand in the docking simulation was evaluated based on the root-mean-square deviation (RMSD) and the intramolecular interactions.

### 2.4. Visualization of Binding Modes and M^pro^–Ligand Interactions

The results were visualized using PyMOL Version 2.4.1 [39]. The hydrogen bonds reported are the ones calculated by PyMOL while hydrophobic and pi-pi interactions were calculated in YASARA. Intramolecular interactions of the most promising compounds were depicted using LIGPLOT v.4.5.3 [40].

### 2.5. ADMET Prediction

The canonical SMILE notations of the selected phytochemicals were obtained through Chemsketch and used as an input to available online software tools that provide data on the druglikeness, pharmacokinetic properties, and toxicity of the compounds. More specifically, SwissADME (http://www.swissadme.ch/index.php accessed on 1 May 2022) was used to obtain data on the physicochemical properties, lipophilicity, water solubility, pharmacokinetics, druglikeness, and medicinal chemistry of the compounds. A free online tool provided by Molsoft (http://molsoft.com/mprop/ accessed on 1 May 2022) was also utilized to calculate the molecular properties and overall druglikeness of the compounds. The toxicity of the compounds under evaluation was predicted through the freely available webserver ProTox II (https://tox-new.charite.de/protox_II/index.php?site=compound_input accessed on 1 May 2022), which calculated the median lethal dose (LD_50_) and provides an estimation of the acute toxicity, hepatotoxicity, carcinogenicity, immunotoxicity, mutagenicity, and cytotoxicity.

### 2.6. Molecular Dynamics Simulation

Molecular dynamics (MD) simulation was performed to further investigate the stability of the complexes of selected docked compounds with M^pro^. The co-crystallization complex of the known inhibitor N3 with M^pro^ (PDB ID: 6LU7) was used as a reference. The simulation was run using YASARA Structure Version 20.12.24 for 10–30 ns, as this was considered a time within which the complexes reached equilibrium. The conditions set for the simulation were a pH of 7.4 [41], NaCl ions at a concentration of 0.9%, temperature of 298 K, and pressure of 1 atm. The setup included optimization of the hydrogen bonding network [42], energy minimization of the system, and definition of a simulation cell 20 Å larger than the protein in every direction. The AMBER14 force field [43] was used for the solute and the Van der Waals forces were calculated using a cutoff of 8 Å (the default used by AMBER [44]) while no cutoff was applied to electrostatic forces, which were calculated using the Particle Mesh Ewald algorithm [45]. The simulation trajectories were saved every 250 ps with a timestep of 2.5 fs for bonded interactions and 5.0 fs for non-bonded interactions [46]. The trajectories were analyzed to show the RMSD of the C-alpha atoms of the complexes, the root-mean-square fluctuation (RMSF) of the protein residues and its radius of gyration (RoG), and the hydrogen bonds between the protease and the ligands.

## 3. Results and Discussion

### 3.1. M^pro^ as a Receptor for Docking Simulations

**Figure 1:**
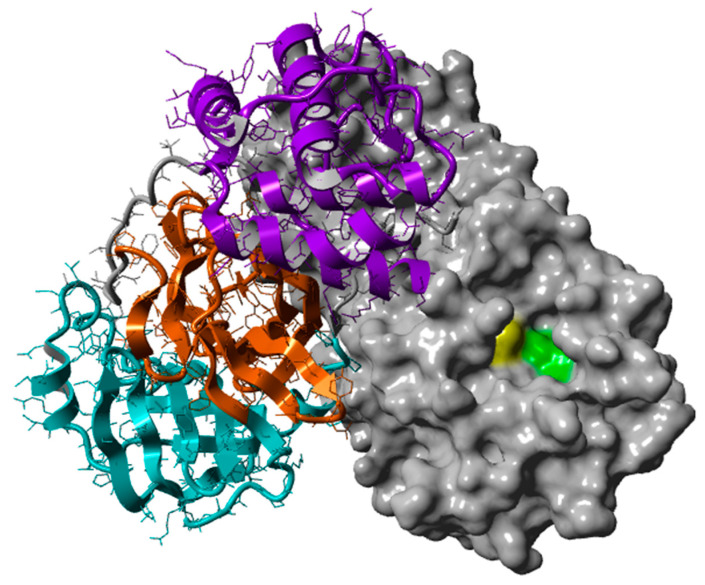
SARS-CoV-2 M^pro^ in the active form of a homodimer (PDB ID: 7JKV). The three domains and their secondary structure are visible in the left monomer (domain I is in cyan, domain II in orange, and domain III in purple). The right monomer is shown as surface, with the catalytic residues His41 and Cys145 being highlighted in green and yellow, respectively.

The role of M^pro^ is the proteolytic cleavage of two viral polyproteins pp1a and pp1ab towards the formation of nonstructural proteins required for further viral reproduction, including its self-release from the aforementioned polyproteins [47,48]. There are more than 11 proteolytic sites, characterized by the sequence (Leu-Gln)-(Ser/Ala/Gly), where the peptide bond being hydrolyzed is the one after Gln [49]. The enzyme functions in a homodimeric form, through a mechanism of nucleophilic addition facilitated by a cysteine-histidine catalytic dyad (Cys145-His41). Each monomer is composed of 306 residues arranged in a polypeptide chain with 3 distinct domains (domain I: residues 8–101; domain II: residues 102–184; and domain III: residues 201–303) (Figure 1). The active site of the enzyme is a cavity formed between domains I and II, which are made of antiparallel β-barrels. Domain III is composed of five α-helices and contributes to the formation of the dimer [50,51,52,53].

Apart from the presence of catalytic residues His41 and Cys145, it is important to mention the existence of four main subsites forming the active site of M^pro^, labeled as S1, S1′, S2, and S4. According to Stoddard et al. [2], the S1 subsite consists of the side chains of Phe140, Asn142, Ser144, Cys145, His163, Glu166, His172, and the backbone of Leu141, Gly143, His164, and Met165; the S1′ subsite is formed by the side chains of Thr25, His41, Val42, Asn119, Gly143, Cys145, and the backbone of Thr26; S2 is created by the side chains of His41, Met49, Tyr54, Asp187, and the backbone of Arg 188; and S4 is made up of the side chains of Met165, Leu167, Pro 168, Ala191, Gln192, and the backbones of Glu166, Arg188, and Thr190. Among them, His41, Gly143, Ser144, Cys145, and Glu166 have been pointed out as residues playing a major role in protein–ligand interactions in molecular dynamics studies [14]. Moreover, residues Gly143, Ser144, and Cys145 form an oxyanion hole capable of stabilizing the negative charge of ligands, such as that of the carbonyl oxygen of the scissile bond in the natural substrate [9,10,53].

Regarding the catalytic mechanism of the enzyme, it is suggested that it is established on the formation of a nucleophilic ion pair, through a proton transfer from the thiol group of Cys145 to the imidazole of His41. The catalytic cysteine attacks the carbonyl of the scissile bond, leading to a thiohemiketal intermediate, while the protonated histidine attacks the N-atom of the peptide bond, creating the acyl–enzyme complex intermediate. The participation of a water molecule in the reaction is of great importance, as it attacks the carbonyl carbon of the substrate’s Gln while the catalytic His is being reprotonated, and also stabilizes the polar contacts between residues His41, His164, and Asp187 by interacting with them. The last step of the mechanism is described by the release of Cys145 through the cleavage of its covalent bond with the peptide [53].

### 3.2. Docking Validation

**Figure 2:**
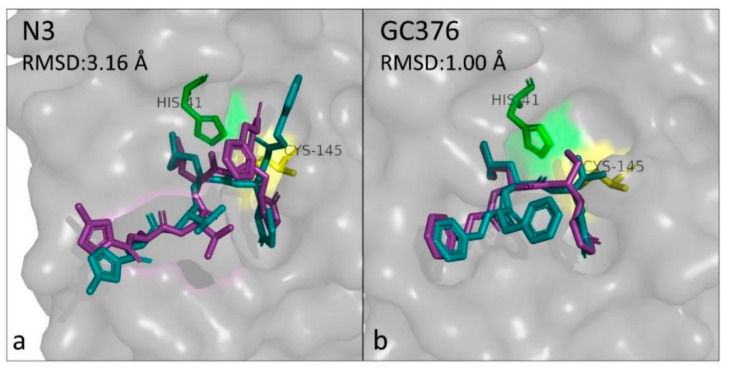
Superimposed binding modes of the inhibitors N3 (**a**) and GC376 (**b**) as occurring from the co-crystallization structure (PDB ID: 6LU7 and 7D1M, respectively) (petrol blue) and the molecular docking simulation (purple), respectively. The root-mean-square-deviation (RMSD) of the binding between the co-crystallized and docked complex is indicated.

Both known inhibitors examined, N3 and GC376, led to a docking output comparable to the co-crystallization structure of their complex with M^pro^. N3 is the most widely accepted and analyzed inhibitor of M^pro^ in the literature [51,54,55]. It is often used as a positive control to provide some reference values with which the binding energy and interactions of a purported inhibitor with M^pro^ can be compared. The binding energy for N3 calculated in this work by Vina is −8.26 kcal/mol, whereas Das et al. [56] reported it to be −7.7 kcal/mol and Ahmed et al. [57] −7.5 kcal/mol, a difference that can be attributed to the different pretreatment of the receptor and the ligand structures before the simulation. Superimposing of the co-crystallized protein–ligand complex and the one calculated by the simulation reveals high similarity between the conformation of N3 in the binding site in the two cases, with an RMSD of 3.16 Å (Figure 2a). In the docking output, the interaction of N3 with Cys145 is a 2 Å hydrogen bond, formed between the pentacyclic ring of N3 and the hydrogen attached to the sulfur atom of the protein residue. Whereas, as determined by the co-crystallization data, it is a 1.8 Å covalent bond between the sulfur atoms of Cys145 and the Cβ atom of the vinyl group of the inhibitor. Although a different part of the inhibitor is bound to the protease, it results in a very similar geometry of the molecule. Moreover, among the seven protein–ligand hydrogen bonds calculated for the co-crystallization structure, involving residues Gly143, His163, His164, Glu166, Gln189, and Thr190, three were also calculated from the simulation (including Glu166 and Gln189). GC376 is another broad-spectrum inhibitor that has demonstrated activity against the main protease of various coronaviruses [58]. In the present simulation, the binding energy calculated for GC376 was −7.80 kcal/mol. The docking output is remarkably similar to the co-crystallized structure, with the ligands in the two conformations having an RMSD as low as 1.0 Å (Figure 2b). In addition, another study featuring the docking of GC376 to PDB 6LU7 with AutoDock Vina reported a binding energy of −8.1 kcal/mol [59], which is very close to the result of our study. The hydrogen bonds that formed appear to be matching to a great extent, as GC376 interacts with His41, Phe140, His164, Glu166, and Gln189 in the co-crystallized complex and with His41, Phe140, His163, Glu166, and Gln189 in the docked complex.

Overall, the docking results for the known inhibitors are highly comparable to the binding modes observed in reality, reinforcing the validity of molecular docking as a tool to provide indications for the inhibitory potential of the compounds under evaluation.

### 3.3. FA as a ‘Reference’ Ligand

**Figure 3:**
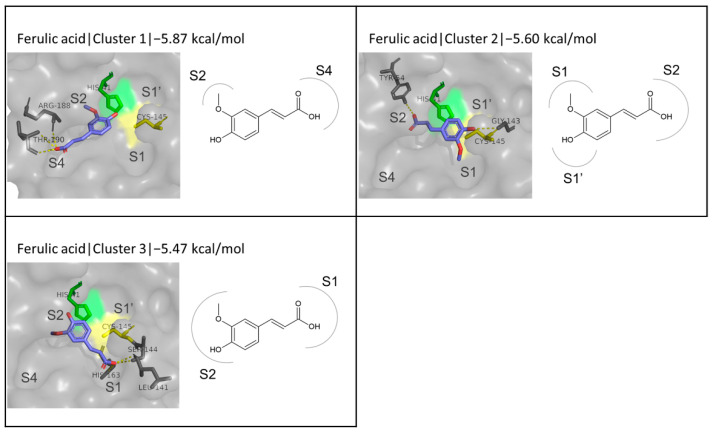
Binding of FA to the active site of M^pro^. The first three clusters resulting from the simulation are presented.

Although FA derivatives have not been extensively studied in silico for their inhibitory potential against SARS-CoV-2 M^pro^, FA itself has been part of related studies featuring natural compounds. The binding energy calculated by Autodock Vina for FA in this work was −5.88 kcal/mol for the first among six emerging clusters. The next two clusters did not deviate much from this value, with binding energies of −5.60 and −5.47 kcal/mol, respectively (Figure 3). In the first and third cluster, the phenolic group is situated in the S2 subsite, but the tail of the molecule extends towards the S4 subsite in the first case and towards the S1 subsite in the second. In the case of the second cluster, the phenolic ring is located between the S1 and S1′ subsites, in front of the catalytic dyad, while the other end of the molecule is inserted into the S2 subsite. His41 stands out as an interacting residue in all cases, involved in both hydrophobic and pi-pi interactions, while hydrogen bond interactions involve residues closer to the catalytic dyad in the case of clusters 2 and 3, and S4 site residues for cluster 1. Using the protease PDB structure 6LU7 and the Molecular Operating Environment 2019.0102 (MOE) software for molecular docking, two different studies reported almost identical binding energies of −5.33 [60] and −5.35 kcal/mol [61], and the formation of two and one hydrogen bonds, respectively, with residues Thr190 and Glu199, and Glu166. The latter also provided the orientation of the molecule when bound to the active site, which resembles the first cluster of this work (Figure 3), without being identical, however.

These results are quite similar with the results produced in the present work for the first cluster of FA, as the binding energies do not differ significantly and there is one common residue involved in hydrogen bonding (Thr190). Another work reported a binding energy of −10.63 kcal/mol and hydrogen bonds with residues Leu141, Gly143, Ser144, Cys145, His163, and Glu166 [62] while the orientation of FA in the active is identical to the one observed in the second cluster for FA in this work. In accordance with the present results, a docking score of −6.0 kcal/mol was reported for FA when the Autodock Vina and PDB structure 6W63 were used [63]. Overall, the docking scores for FA are quite low compared to established inhibitors such as the previously mentioned N3 (−8.26 kcal/mol) and GC376 (−7.80 kcal/mol). Therefore, the screening of FA derivatives aims to explore their potentially improved properties both in M^pro^ inhibition and in terms of pharmacokinetics.

### 3.4. Docking of Enzymatically Synthesized FA Derivatives

Enzymatic synthesis of FA derivatives has been prevalently dominated by esterification of FA or transesterification of a respective activated donor (such as methyl ferulate, MFA or vinyl ferulate, VFA), under a low water content towards the production of stabilized esters with tailored lipophilicity [36,64,65]. Esterases that are widely used for this purpose are the triaglycerol lipases (EC 3.1.1.3) due to their broad specificity towards glycerides and related substrates. Nevertheless, feruloyl esterases (EC 3.1.1.73), which have specificity towards hydroxynnamic acids, have also been employed for this purpose as they can offer the unique advantage of catalyzing the synthesis of esters with a wide range of substitutions on the phenolic ring, resulting in either more lipophilic (e.g., alkyl esters) or hydrophilic esters (e.g., sugar esters) [66,67,68,69,70]. Transglycosylation of FA with the glucoside rutin has been described [35]. An overview of the major FA derivatives that have been synthesized enzymatically along with the representative synthesis routes is described in Table 1. These derivatives were selected for further in silico simulations. Table 2 presents the results of the docking simulations for the major enzymatically synthesized derivatives.

#### 3.4.1. Alkyl and Alkenyl Esters of FA

The FA derivatives belonging to this category that were investigated include methyl, ethyl, propyl, butyl, isobutyl, pentyl, isopentyl, prenyl, hexyl, octyl, dodecyl, and octadecyl ferulates. Overall, the calculated binding energies ranged from −5.20 to −6.75 kcal/mol, with prenyl ferulate exhibiting the highest binding affinity to the active site of M^pro^. Although there is not a definite correlation between the binding energy and the potential of in vitro inhibitory effect, it is not encouraging that these values are significantly lower than the inhibitors N3 and GC376. The total results in terms of the binding energies and interactions calculated are shown in Table 2. It is interesting that prenyl ferulate is the only alkenyl ester among this category, and has better binding compared to the respective alkyl ester (isopentyl ferulate, −6.58 kcal/mol). It is observed that the binding energies become progressively lower as the carbon chain of the substitutions becomes larger, until the point of five carbons. For the larger substitutions, the binding affinity starts to decrease again.

**Figure 4:**
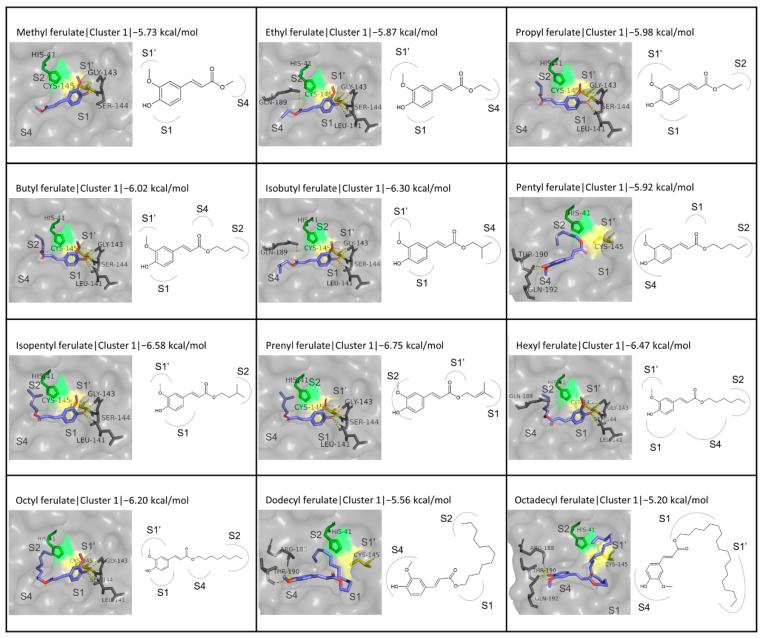
Structures and binding modes of alkyl and alkenyl FA esters in the active site of M^pro^.

The prevalent conformation observed is the one where the phenolic ring of the FA moiety is stabilized at the S1 subsite, in front of the catalytic residue Cys145, while the carbon chain extends towards the S2/S4 subsites. This particular orientation is seen in the first cluster for all ligands in this category, except for pentyl ferulate, where the orientation corresponds to the second cluster (Figure 4). The binding energy is almost identical between the two clusters. Moreover, no such conformation is observed in the cases of dodecyl and octadecyl ferulate, which possess a long carbon chain that folds differently into the active site.

Another commonly occurring geometry is characterized by the phenolic moiety of the ligands being located at the S2 subsite while the rest of the molecule is orientated towards the S1 or S1′ subsites, as seen, for example, in the case of the third cluster for methyl ferulate or the second cluster for butyl ferulate, respectively (Figure S1). However, taking into consideration the facts that this conformation does not appear as consistently as the previously described one and, when it does, it represents the second or third clusters emerging from the simulation, which often have a considerably lower binding affinity to the active site of the protease, it can be suggested that the most likely position for the phenolic ring is the one in front of the catalytic cysteine. In this spatial arrangement of the molecule, the hydroxyl substitution, which readily contributes to the formation of hydrogen bonds, appears to play an important role in the stabilization of the molecule in a position where access to one of the two catalytic residues is restricted, therefore potentially resulting in more effective inhibition of the catalytic activity of the enzyme.

Regarding comparable results in the literature, the binding of methyl ferulate to M^pro^ (PDB ID:6W63) was simulated with AutoDock 4.2, which calculated a binding energy of −5.19 kcal/mol, and the formation of hydrogen bonds with the residues Thr190 and Gln192 [100]. The binding energy is comparable to the one calculated in this work (−5.73 kcal/mol), but the hydrogen bond interactions are located in different parts of the active site, as the present study indicated interactions around the catalytic dyad with residues Gly143, Ser144, and Cys145 for the first cluster.

#### 3.4.2. Fatty Esters of FA and Other Related Esters

This category of compounds includes oleyl, glyceryl, diglyceryl, tocopheryl, and β-sitosteryl ferulates. The binding energies calculated via the docking simulation range from −5.17 to −7.81 kcal/mol. The lowest binding affinity was predicted for oleyl ferulate, which has an 18-carbon aliphatic chain attached to the FA moiety and exhibits similar results to these of octadecyl ferulate, which possesses an equally long carbon chain, both in terms of the binding energy (−5.17 and −5.20 kcal/mol, respectively) and the conformation of the first cluster. Glyceryl, diglyceryl, and tocopheryl ferulates do not fall below −7.0 kcal/mol, thus exhibiting a lower binding affinity compared to the inhibitors N3 (−8.26 kcal/mol) and GC376 (−7.80 kcal/mol), which is, however, not high enough to exclude the possibility of effective inhibition. The most promising results were yielded for β-sitosteryl ferulate in the first cluster, where the triterpene moiety of the molecule occupies the S4 and S1 subsites while the FA moiety extends to the S1′ subsite and out of the active site cavity. This ligand emerges as particularly promising, as its binding energy (−7.81 kcal/mol) is very close to that of GC376 (−7.80 kcal/mol). β-Sitosteryl ferulate also forms three hydrogen bonds, with residues Thr25, Gly143, and the catalytic His41. Another molecular docking study also using Autodock Vina and PDB structure 6LU7 reported an identical binding energy of −7.8 kcal/mol for β-sitosteryl ferulate; however, in this case, the compound appears to bind to M^pro^ at a different site [101].

**Figure 5:**
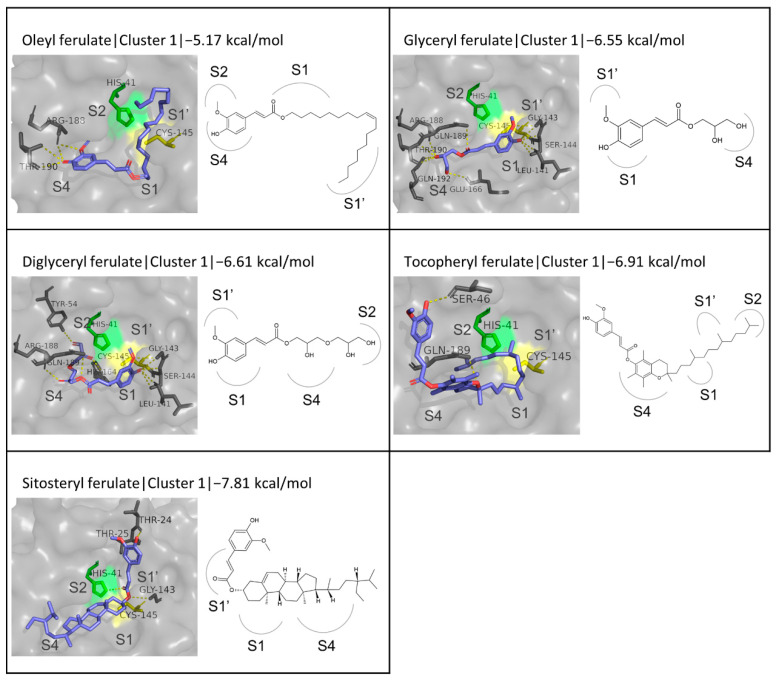
Structures and binding modes of fatty acid, polyol, tocopherol, and sterol FA derivatives at the active site of M^pro^.

Regarding other patterns observed in the binding mode to the active site, it is observed that the smaller ligands in this category (glyceryl and diglyceryl ferulate) bind to the active in a similar manner as the FA derivatives described in the previous paragraph, where the FA phenolic moiety is situated in front of Cys145 at the S1 subsite, and its hydroxyl group participates in hydrogen bonding with the neighboring residues while the tail of the molecule is orientated towards the S4/S2 subsites. This orientation is seen in the first cluster of both glyceryl and diglyceryl ferulate. As far as the molecules with the larger carbon chains are concerned (oley and tocopheryl ferulates), no binding patterns were observed while the orientation of tocopheryl ferulate in all three clusters appeared to be more efficiently blocking access to the catalytic dyad compared to oleyl ferulate, where the FA moiety mostly occupied the active site. The structures of the ligands and the conformations described above are shown in Figure 5 for cluster 1 and Figure S2 for clusters 2 and 3.

#### 3.4.3. Sugar Esters of FA

Several FA sugar derivatives were included in this study, as they are an interesting case of enzymatically synthesized derivatives with improved water solubility. The derivatives studied in this work include d-glucose, d-fructose, d-galactose, d-mannose, l-arabinose, d-xylose, d-lactose, d-sucrose, d-maltose, d-cellobiose, Galactobiose, Xylobiose, Raffinose, Arabinobiose, fructooligosaccharide (FOS 1, 2, and 3), d-mannitol, d-sorbitol, and d-xylitol ferulates.

**Figure 6:**
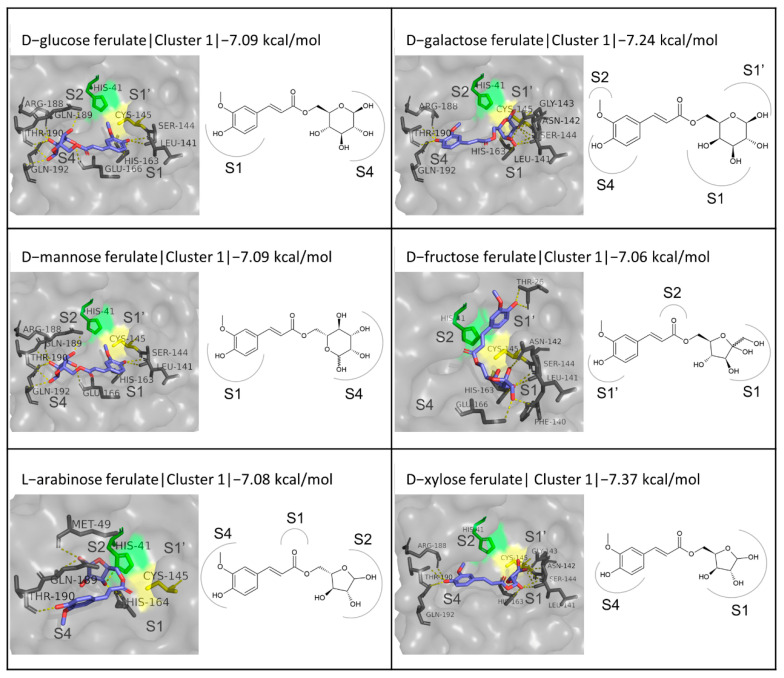
Structures and binding modes of the monosaccharide esters of FA at the active site of M^pro^.

The range of binding energies calculated for the monosaccharide-based esters is quite narrow, ranging from −7.06 kcal/mol for d-fructose ferulate, which is the only FA derivative with a keto-hexose tested, to −7.37 kcal/mol for d-xylose ferulate. In comparison to the inhibitors N3 and GC376, these results are not exceptional, but they are comparable. Therefore, also taking into consideration the improved solubility of these compounds, these sugar esters of FA are worthy of further investigation. As far as the first clusters are concerned, two orientations are more often seen. One is characterized by the positioning of the penta- or hexacyclic ring in the S1 subsite, close to the catalytic Cys145, and the extension of the rest of the molecule horizontally, towards the S4 (or sometimes S2) subsite, as in the case of the first cluster for d-galactose ferulate (Figure 6). With the exception of d-glucose and d-mannose ferulates, where the FA moiety occupies the S2 subsite, and l-arabinose ferulate, for which this conformation is not observed at all in the first three clusters, the interactions observed are almost identical and include hydrogen bonds with residues Leu141, Asn142, Gly143, Ser144, Cys145, His163, Arg188, Thr190, and Gln192. The second most prevalent orientation resembles the dominant orientation described in the previous paragraphs, and is opposite to the aforementioned one, with the phenolic ring of FA being stabilized in front of Cys145 at the S1 subsite and the sugar substitution blocking the S4 subsite. The residues more often involved in hydrogen bonding in this case are quite similar, including Leu141, Gly143, Ser144, Glu166, Thr190, and Gln192. Overall, based on the frequency of occurrence of the presented conformations in the first three clusters resulting from the simulation (Figure 6 and Figure S3), the first binding mode described is the prevailing one. This supposition is supported by the fact that in the cases where this geometry appears in the second cluster instead of the first, the resulting binding energies of the two clusters are almost identical. An example is d-glucose ferulate, with a binding energy of −7.09 kcal/mol for the first cluster and −7.08 kcal/mol for the second cluster.

**Figure 7:**
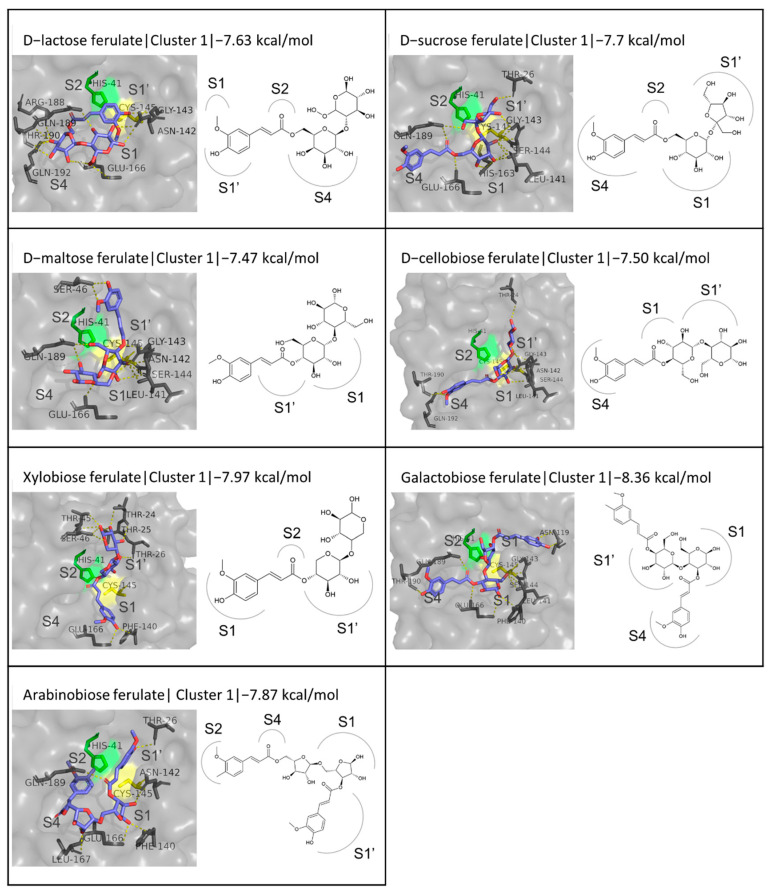
Structures and binding modes of disaccharide esters of FA at the active site of M^pro^.

The sugar esters of FA also include coupling with disaccharides d-lactose, d-sucrose, d-maltose, d-cellobiose, and xylobiose. Their binding energies provide a positive indication of the inhibitory potential, as they fluctuate within a small range comparable to that of the reference inhibitors, from −7.47 kcal/mol for d-maltose ferulate to −7.97 kcal/mol for xylobiose ferulate. Three binding patterns stand out from the evaluation of the first three clusters for each compound, with only one of them being present in all the cases. This involves the FA moiety blocking the S4 subsite, the monosaccharide closer to FA taking up the S1 subsite, and the second monosaccharide extending upwards at the S1′ subsite, as seen in the case of the third cluster for d-lactose ferulate or the first cluster for d-sucrose ferulate (Figure 7).

The occupation of the same regions of the active site is, however, not translated into identical hydrogen bond interactions. The most commonly occurring ones involve residues Gly143 and Thr190 in four out of five ligands and Thr26 in three out of five. Another conformation observed in four out of the five compounds (e.g., the first cluster for d-maltose ferulate) is a vertical one, with the cyclic ring of the sugar closer to FA blocking the catalytic cysteine between the S1 and S1′ subsites and the FA extending towards S1′. An evident motif is observed in the interactions between the compounds in this orientation and the protease active site, with Ser46, Gly143, Ser 144, Cys145, and Glu166 emerging as hydrogen bond hotspots. Lastly, a conformation involving the occupation of all four subsites, S4 by the phenolic ring of FA, S2 by its carbonyl group, S1′ by the monosaccharide ring closer to FA, and S1 by the second monosaccharide, respectively, is observed in two of the five cases (in the second cluster of D-lactose ferulate and the third clusters of D-sucrose ferulate (Figure S4). This binding pattern is stabilized by hydrogen bonds of the ligands with residues Phe140, Leu141, Asn142, Gly143, His163, and Glu166.

Two other disaccharide derivatives include two FA groups, galactobiose ferulate and arabinobiose ferulate, with very promising binding energies of −8.36 and −7.88 kcal/mol, respectively, the former of which is the second highest among the enzymatic derivatives tested. When observing the first three clusters for the two ligands, only the first cluster for galactobiose ferulate had an orientation comparable to any of the binding patterns detected for the other disaccharide derivatives. This conformation involves the two monosaccharides occupying the S1 and S1′ subsites and one of the FA groups extending to the S4 subsite while the other is located above the S1′ subsite, emerging out of the active site cavity (Figure 7).

**Figure 8:**
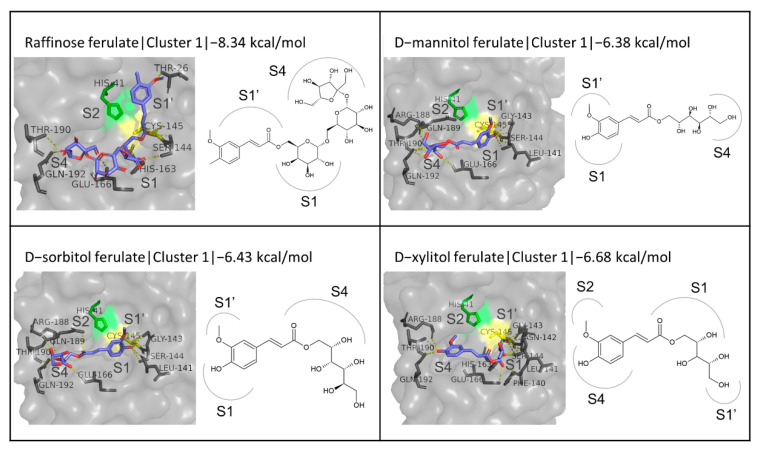
Structures and binding modes of trisaccharide and polyol esters of FA at the active site of M^pro^.

The only trisaccharide esterified with one FA moiety tested is raffinose ferulate, which exhibited the third best binding affinity (−8.34 kcal/mol) to M^pro^ among the enzymatic derivatives studied, which also surpasses that of the native inhibitor N3. These results were only exceeded by galactobiose ferulate and one of the structures examined for FOS ferulate (FOS ferulate 3), which will be further described below. Raffinose ferulate forms eight hydrogen bonds with residues Thr26, Ser144, Cys145, His163, Glu66, Thr190, and Gln192, most of which are between the hydroxyl groups of the sugar moieties and residues located at the S1 and S4 subsites. The binding mode of the compound as predicted by the docking simulation appears to be limiting access to Cys145. (Figure 8). Another category of compounds tested is sugar alcohol esters, and, more specifically, the FA esters with D-mannitol, D-sorbitol, and D-xylitol. These compounds had the lowest binding affinity to the active site among the sugar derivatives of FA: −6.38, −6.43, and −6.68 kcal/mol, respectively. In this category, the conformation where the FA ring is in front of Cys145, between the S1 and S1′ subsites, while the polyol is orientated towards the S4 subsite is the one that prevails in the first clusters and is stabilized in all three cases with hydrogen bonds with residues Leu141, Gly143, Ser144, Cys145, Glu166, Arg188, Thr190, and Gln192 (Figure 8).

**Figure 9:**
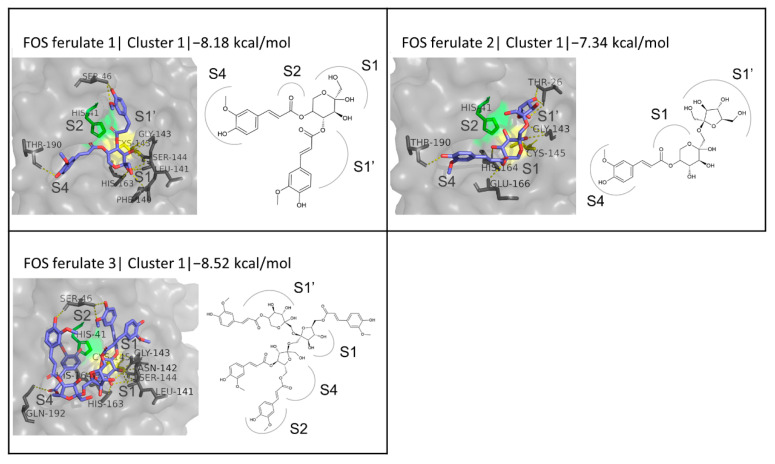
Structure and binding of the three suggested FOS ferulate structures at the active site of M^pro^.

Fructooligosaccharides (FOSs) are oligomers of fructose units linked by beta (2-1) glycosidic bonds, with the terminal unit often being a glucose. These oligosaccharides can comprise 2 to 60 monomers [102]; therefore, their exact structure is unknown. In this work, the structures of feruloylated FOS fragments proposed by Couto et al. [99] were adopted for the purposes of the simulation. It is stated in the original work that the position of the FA moieties on the oligosaccharide backbone is not confirmed, and the structures proposed are indicative. They include three forms of FOS ferulate, here referred to as FOS ferulate 1, 2, and 3. FOS ferulate 1 is a fragment that is composed of two FA moieties esterified to the O4 and O5 positions of a fructose and exhibits a binding energy of 8.175 kcal/mol, which is significantly high if compared with the rest of the monosaccharide FA esters. This value is very encouraging, being also highly comparable to the respective values for the reference inhibitors. Although FOS is not a monosaccharide, the orientation of the studied monosaccharide fragment, FOS ferulate 1, in its first cluster resembles the prevalent conformation described above for the rest of the monosaccharide-based derivatives, where the monosaccharide is located below Cys145 and the one FA group occupies the S4 subsite while the other extends upwards to block the S1′ subsite (Figure 9). Hydrogen bonds with residues Leu141, Gly143, Ser144, His163, and Thr190 are also common interactions in both cases. FOS ferulate 2 is a fragment depicting two fructose moieties linked with a (1-2) glycosidic bond, where FA is esterified to the O5 position of the first monomer. It exhibited an encouraging binding energy of −7.34 kcal/mol and 8 hydrogen bonds with residues commonly involved in ligand binding, namely Thr26, Gly143, His164, Glu166, and Thr190. As in the case of FOS ferulate 1, although FOS ferulate 2 is an arbitrarily defined fragment rather than a disaccharide, the binding mode of its first cluster can be compared to that of the disaccharides described above, as it follows the predominant pattern observed, where FA blocks the S4 subsite, the fructose linked to it occupies the S1 subsite, and the other fructose monomer extends to the S1′ subsite. FOS ferulate 3 is the largest of the molecules tested, including a fructose trimer with four FA groups esterified to the O5 position of the first monomer, the O6 of the second, and the O4 and O6 of the third, respectively. It showed the highest binding affinity (−8.52 kcal/mol), utilizing its volume to take up the entire active site of M^pro^ and being stabilized through 14 hydrogen bonds, with residues Ser46, Leu141, Asn142, Gly143, Ser144, Cys145, His163, His164, and Gln192.

Conformations for the second and third cluster results for the simulations regarding trisaccharide, polyol, and polysaccharide esters are presented in Figures S5 and S6.

#### 3.4.4. FA Glycosides

**Figure 10:**
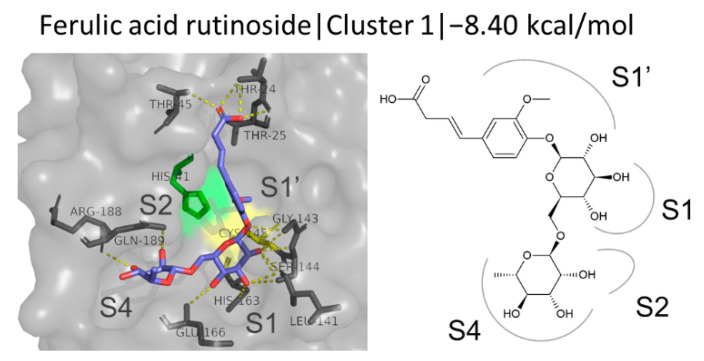
Structure and binding mode of FA rutinoside at the active site of M^pro^.

FA rutinoside is a rutinase-synthesized glycoside, which exhibited an exceptionally high binding affinity and a binding energy of 8.404 kcal/mol. It forms several hydrogen bonds with residues Thr24, Thr25, Thr45, Leu141, Gly143, Ser144, Cys145, His163, Glu166, Arg188, and Gln189. The docking simulation output suggests that it binds to the active site of M^pro^ blocking the S1, S1′, and S4 subsites, with its glucosyl group being stabilized through multiple hydrogen bonds at the S1 subsite, the rhamnosyl group occupying the S4 subsite, and the FA moiety extending upwards into the S1′ subsite (Figure 10). The conformations of the second and third cluster are shown in Figure S7.

### 3.5. Docking of Chemically Synthesized FA Derivatives

The chemically synthesized FA derivatives included in this study were selected based on their promising results in published works regarding antiviral effects (Table 3). The studies from which the derivatives were selected involve the synthesis of several derivatives and their in vitro evaluation against a range of viruses. The ones selected in this work for investigation are a selection of the compounds that showed the best antiviral potential against the respective tested virus. Such derivatives fall into the categories of FA amides, FA oligomers, and FA derivatives with dithioacetal, acylhydrazone, quinazoline, and chalcone moieties.

**Figure 11:**
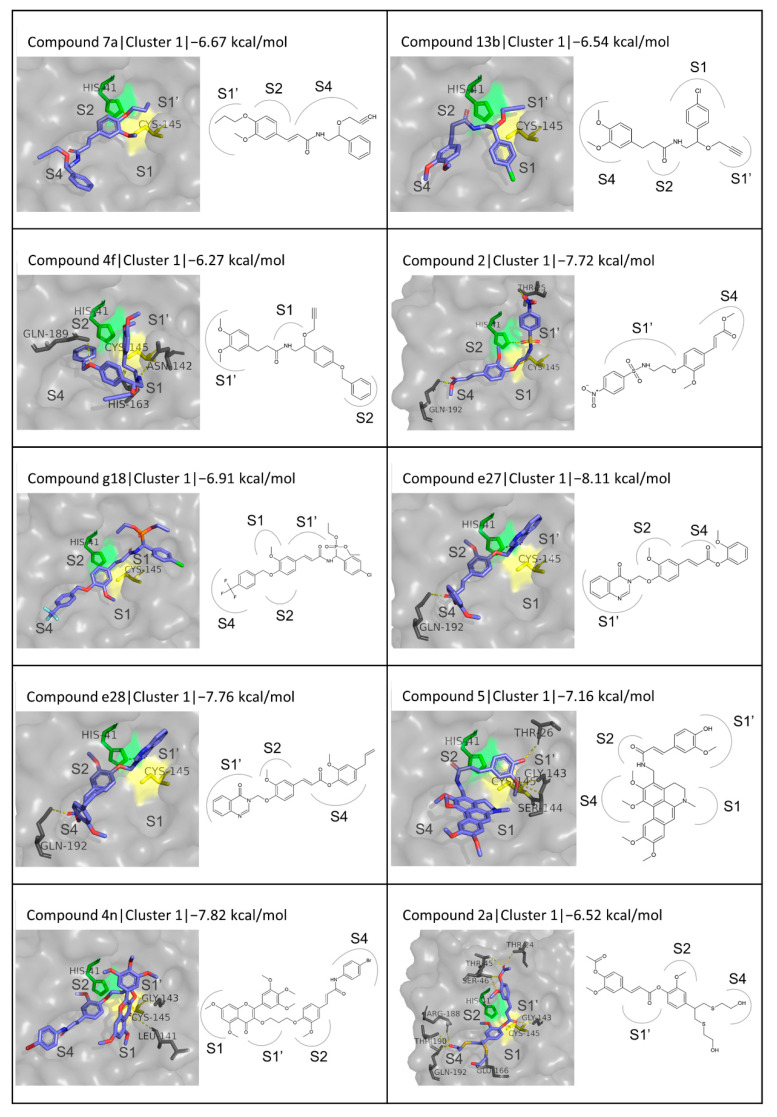

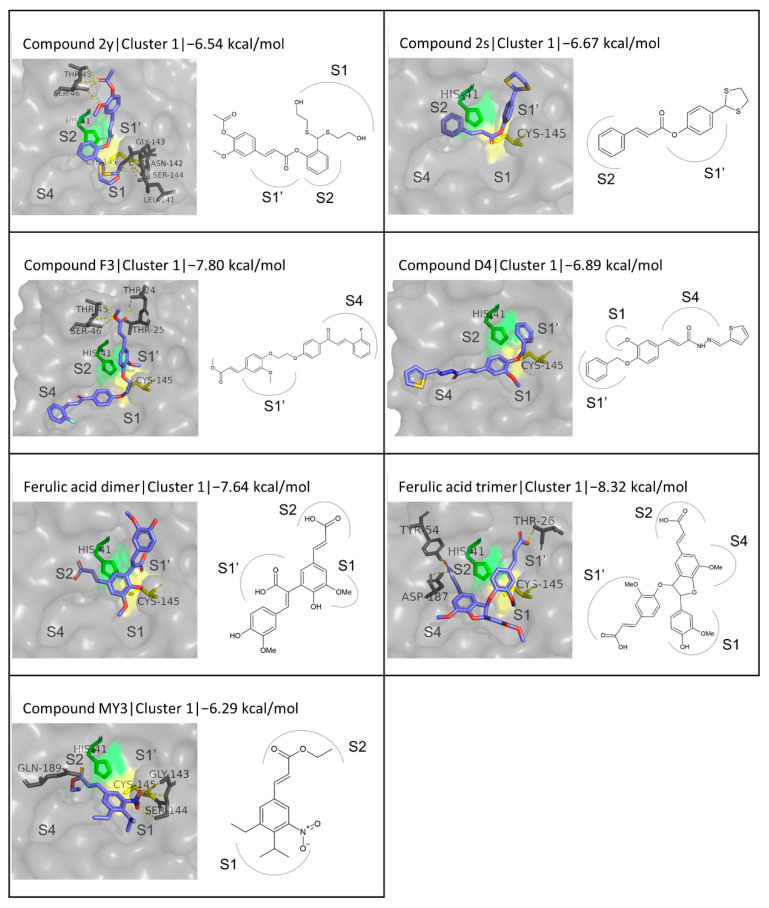
Structures and binding modes of chemically synthesized FA derivatives in the active site of M^pro^.

The considerable variety in the structure of the ligands in this category is reflected in a broad range of binding energies (from −6.27 to −8.40 kcal/mol) and a wide diversity of binding modes. The binding energy and detailed interactions for each compound are presented in Table 4. A general observation about the binding of the chemically synthesized derivatives, as seen in Figure 11, is that in a considerable number of them, for cluster 1, the phenolic moiety in FA appears to be located between the S2 and S1′ subsites, more towards S2. In this position, it is in the close vicinity of the catalytic dyad, as seen, for example, in the cases of compounds **7a**, **2**, and **g18**. Particularly in the cases of compound D4, FA dimer, and trimers, the phenolic ring is stabilized closer to the S1´ subsite, blocking access to the area around the catalytic residues even more effectively. The binding modes of the second and third clusters were also taken into consideration, but no evident binding pattern was observed (Figure S8).

The compounds that stood out are two FA derivatives with a quinazoline moiety, namely **e27** and **e28**, with binding energies of −8.11 and −7.76 kcal/mol respectively; **4n**, an FA amide and myricetin derivative with a binding energy of −7.82 kcal/mol; F3, an FA-chalcone ester with a binding energy of −7.80 kcal/mol; and triferulic acid, which exhibited a binding energy of −8.32 kcal/mol. The two quinazoline derivatives form hydrogen bonds with His41 and Gln192; the myricetin derivative with residues Leu141 and Gly143; the chalcone derivative with Thr24, Thr 25, Thr45, and Ser46; and the FA trimer with Thr26, Tyr54, and Asp187. Moreover, Gln189 and Met165 often participate in hydrophobic interactions while His41 appears to be involved in pi-pi stacking. Although the interactions do not exhibit any evident pattern, the ligands in all the cases appear to be occupying the area in front of the catalytic dyad, providing a positive indication of potential inhibitory activity. Compounds e27 and e28 have a very similar structure, which results in an almost identical binding mode. It is also interesting that in the case of compound **4n**, where the respective substitution is linked to FA at the same position of FA as in the case of e27, and e28 (at C4 of the phenolic ring) binds to the active site in a way that the FA moiety has the same position as in the quinazoline derivatives. Its bulkier substitution, however, allows for higher coverage of the active site and occupation of the S1 subsite as well, as opposed to only S4, S2, and S1´ in the case of e27 and e28. The FA trimer also appears to be quite flexible and bends in a way that allows blocking of all four subsites while F3 mostly occupies the S4 and S1´ subsites and is stabilized through hydrogen bonds in the upper part of the latter.

### 3.6. Summary of Docking Results

Taking into consideration the binding scores of the confirmed M^pro^ inhibitors GC376 and N3 (−7.80 and −8.26 kcal/mol, respectively), the compounds with a binding energy of −7.8 kcal/mol or lower can be highlighted as the most promising ones. These are namely FOS ferulate 3 (−8.52 kcal/mol), FA rutinoside (−8.40 kcal/mol), galactobiose ferulate (−8.36 kcal/mol), raffinose ferulate (−8.34 kcal/mol), FA trimer (−8.32 kcal/mol), FOS ferulate 1 (−8.18 kcal/mol), compound e27 (−8.11 kcal/mol), xylobiose ferulate (−7.97 kcal/mol), arabinobiose ferulate (−7.88 kcal/mol), compound **4n** (−7.82 kcal/mol), β-sitosteryl ferulate (−7.81 kcal/mol), compounds F3 (−7.80 kcal/mol) and e28 (−7.76 kcal/mol), and d-sucrose ferulate (−7.77 kcal/mol), in a series of decreasing binding affinity. It is observed that among these hits, the sugar derivatives form significantly more hydrogen bonds with the active site residues, a fact that can be attributed to the presence of several hydroxyl groups. Leu141, Gly143, Ser144, Cys145, His163, and Glu166 very often appear to be involved in these interactions, which is in consonance with the literature that highlights Gly143, Ser144, Cys145, and Glu166 among the key interacting residues, as indicated by computational tools such as molecular dynamics simulations [14,112].

### 3.7. ADMET Properties of the Selected FA Derivatives

#### 3.7.1. Bioavailability and Druglikeness

The estimation of the druglikeness of the compounds under investigation, performed using the SwissADME and MOLSOFT online servers, is presented in Table 5. The size, flexibility, polarity, and solubility/lipophilicity of a compound, and its ability to form hydrogen bonds, affect how easily it can permeate membranes, be absorbed, distributed, and reach and bind to biological targets. More specifically, low lipophilicity hinders the transfer of a compound through cell membranes, but high lipophilicity is usually related to low absorption and high metabolic turnover, and potential toxicity [113]. The majority of the sugar derivatives are less lipophilic than FA while the aliphatic and chemically synthesized derivatives are more lipophilic.

Water solubility is principally a desired property. Almost all the FA derivatives have acceptable solubility, but the derivatization itself causes an increase in the solubility compared to FA mostly in the case of the sugar derivatives. As expected, the alkyl and alkenyl derivatives become progressively less soluble in water as the carbon chain size of the substitution increases. Oleic acid, tocopherol, and sitosterol esters are poorly soluble in aqueous media. As far as the chemically synthesized derivatives are concerned, they display moderate to low solubility. Hydrogen bond donors and acceptors can contribute to the solubility and binding to the desired targets, but the presence of too many of such groups negatively impacts the permeability through cell membranes [114]. The derivatives tested possess an adequate amount of hydrogen bond donors and acceptors, aside from FA sugar derivatives, in which a high number of such groups is observed.

According to Daina et al. [115], the optimum lipophilicity, size, polarity, insolubility, insaturation, and flexibility for a compound to be considered as having good bioavailability correspond to logP_o/w_ values ranging between −0.7 and +5.0, molecular weight between 150 and 500 g/mol, topological polar surface area (TPSA) between 20 and 130 Å^2^, logS between 0 and 6, fraction of carbons in the sp^3^ hybridization between 0.25 and 1, and no more than 9 rotatable bonds. Based on these rules, more than half of the compounds have favorable properties in most of the categories. However, the most positive oral bioavailability indications are for the compounds that have less good binding scores.

Another rule for easily evaluating oral bioavailability is the Lipinski’s rule of 5, according to which a compound is likely to be orally bioavailable if it has less than 5 hydrogen bond donors and less than 10 acceptors, molecular weight lower than 500 g/mol, and an octanol-water partition coefficient lower than 4.15. It is hopeful that 41 out of the 57 compounds satisfy the Lipinski´s rule of 5 regarding oral bioavailability, even though the best hits from the molecular docking simulation are not included in those. However, it is important to note that this is not a strict criterion that can certainly exclude a compound from further investigation. For example, 13.6% of the oral and 50.0% of the non-oral drugs with low solubility and permeability violate the rule [116]. The bioavailability score is another index that demonstrates a similar tendency for the compounds tested as the two previously described criteria.

Overall, the druglikeness scores calculated for the investigated compounds, based on the respective index calculated by MOLSOFT, do not exclude them from being used as drugs. The index is calculated by comparing the molecular properties of the respective compound with a library of selected drug and non-drug compounds [117] and provides an indication of how likely a compound is to function as an oral drug. Scores between 0 and 1 are stronger indicators of druglikeness, but also negative values, particularly over −1, correspond to resemblance to drug molecules. The alkyl and alkenyl derivatives had the most negative scores, the lowest being −0.76, while the FA sugar esters and chemically synthesized derivatives showed more encouraging results, with tocopheryl and sitosteryl ferulate and compound 5 yielding the best scores, with druglikeness scores of 1.14, 1.21, and 1.40, respectively. It is worth mentioning that isobutyl and isopentyl ferulates stood out among the alkyl derivatives in terms of druglikeness, the sugar polyol esters exhibited lower scores compared to the other sugar derivatives, and the chemically designed molecules had an overall higher druglikeness, something that could be expected given the fact that many of them emerged from drug design procedures.

#### 3.7.2. Pharmacokinetics

Computational evaluation of the pharmacokinetic properties of the screened compounds using SwissADME showed high gastrointestinal absorption for the majority of the alkyl and alkenyl FA esters and chemically synthesized FA derivatives but low gastrointestinal absorption for the majority of the FA sugar esters (Table 6).

#### 3.7.3. Toxicity Profile

Altogether, the compounds were predicted to have low toxicity, with LD_50_ values being greater than 5000 mg/kg for the majority of them, and only a few alkyl, alkenyl, and fatty acid derivatives having a lethal dose lower than 1000 mg/kg. The prediction accuracy estimated by ProTox-II was around 70% for the vast majority of the compounds. Almost all appeared likely to be immunotoxic, but hepatotoxicity, carcinogenicity, mutagenicity, and cytotoxicity were predicted to be of no concern (Table 6).

### 3.8. Summary of the ADMET Properties of the Most Promising Compounds

Regarding the compounds with the best binding scores, namely FOS ferulate, FA rutinoside, galactobiose ferulate, raffinose ferulate, FA trimer, FOS ferulate 1, compound e27, xylobiose ferulate, arabinobiose ferulate, compound 4n, β-sitosteryl ferulate, compounds F3 and e28, and d-sucrose ferulate, the pharmacokinetic properties were not optimal. Among the best hits, only e27 has molecular weight, rotatable bond, polar surface area, logP, and logS values that indicate good oral bioavailability. The chemically synthesized derivatives, excluding 4n, which showed poor ADMET properties, are the only hits for which an adequate bioavailability score, good balance between water solubility and lipophilicity, and compliance with the Lipinski´s rule were predicted. On the other hand, the enzymatically synthesized derivatives, comprising the sugar esters of FA, exhibit higher solubility and significantly lower acute toxicity, portrayed in the lethal dose values. Only compounds **e27**, **e28**, and **F3** were predicted to have high gastrointestinal absorption while all of them appear to be unable to cross the BBB. Regarding selectivity, very few are assessed as cytochrome inhibitors, whereas none are a P-glycoprotein substrate.

Based on all the above, FOS ferulate 1 would appear to have the highest balance between lipophilicity and solubility among the enzymatically synthesized derivatives, and an adequate druglikeness score, slight deviation from the oral bioavailability criteria (compared to the rest of the compounds), and a relatively high lethal dose. However, due to their higher binding scores, exceptional solubility, and the fact that they can be more easily synthesized, FA rutinoside and raffinose ferulate are also highlighted as promising hits. Regarding the chemically synthesized derivatives, compound e27 appears to have an edge over the rest of the compounds in all the categories, with the only concern being its relatively low lethal dose value. In any case, despite being a useful indication, these predictions are not definite and cannot exclude the compounds from further evaluation for their potential utilization as drugs or nutraceuticals.

### 3.9. MD Simulation of Selected Derivatives

**Figure 12:**
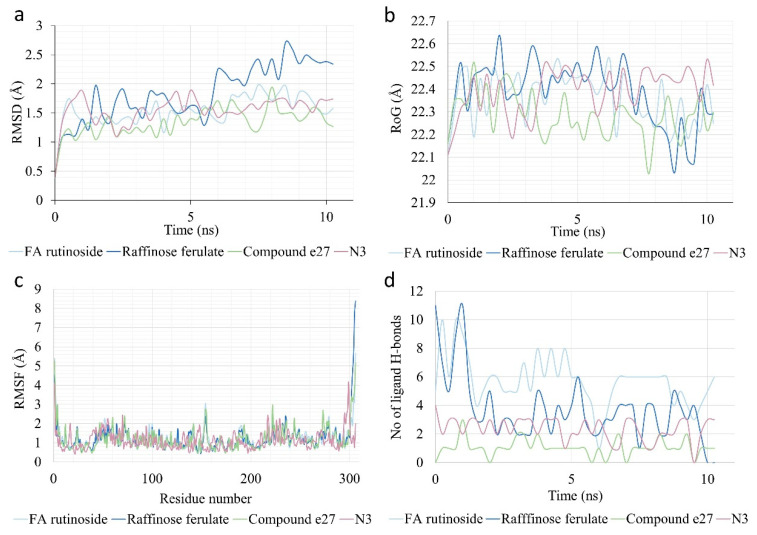
(**a**) RMSD values of the C-alpha atoms of the complexes of the ligands FA rutinoside, raffinose ferulate, compound e27, and reference inhibitor N3 with M^pro^ throughout the simulation time; (**b**) Radius of gyration of the complexes throughout the simulation time; (**c**) RMSF values of residues of M^pro^ in complex with the ligands; (**d**) Number of hydrogen bonds formed between the ligands and M^pro^ throughout the simulation time.

MD simulation was performed to more thoroughly evaluate the stability of the complexes of three ligands that emerged as particularly promising from the molecular docking simulation (raffinose ferulate, FA rutinoside, and compound e27) with SARS-CoV-2 M^pro^ while the complex of the protease with the native inhibitor N3 was also analyzed to provide a reference for the evaluation of the results. The complexes appeared to be particularly stable (Figure 12a), with overall low values and subtle fluctuation of the RMSD (1–2 Å) for FA rutinoside and compound **e27**, with the results for the latter indicating a complex that is more stable than that of N3 for the majority of the simulation time. Raffinose ferulate exhibited slightly higher RMSD values, which exceeded 2 Å after 6 ns. Although its RMSD values appear to have an ascending tendency, the simulation was run for 30 ns to confirm that equilibrium indeed occurred after 10 ns (results not shown). In any case, the RMSD values of the complex of raffinose ferulate are still below 3 Å, which is considered low enough to characterize the complex as stable [119].

The radius of gyration is a useful measure of the rigidity of the protein–ligand complexes. All the complexes appeared to have comparable results (Figure 12b), which can be regarded as indicators of compactness and stability [120,121]. Compound e27 showed the tightest complex with M^pro^. FA rutinoside had very similar RoG compared to N3, and raffinose ferulate exhibited a greater variation in its RoG values, which, however, still compose a low RoG profile. Analysis of the RMSF values allows the acquisition of an image of which residues are more flexible. As seen in Figure 12c, the majority of residues have low RMSF values below 1 Å [120,122] while the terminal residues of the protease exhibit a much larger deviation and are the only ones to exceed 2.5 Å, with the exception of Arg222, Tyr154, Gln273, and Asp155 for compound e27. Therefore, the structure of the protein appears to be quite stable.

**Figure 13:**
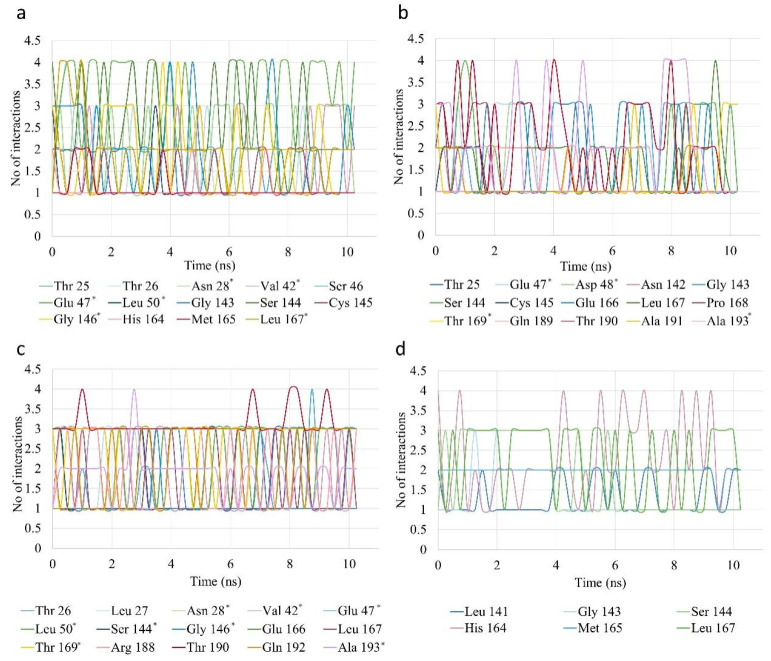
Number of contacts of the ligands (**a**) FA rutinoside, (**b**) raffinose ferulate, (**c**) compound e27, and (**d**) N3 with M^pro^ residues throughout the simulation time. The residues marked with an asterisk (*) are residues that appeared to have contact with the ligand in the MD simulation but were not given as contacting residues in the molecular docking simulation output. However, they are neighboring contacting residues. This can be due to the fact that since MD trajectory analysis uses the distance as a parameter to calculate contacts, sometimes atoms from neighboring residues can be within a distance small enough to be regarded as contacts.

As far as protein–ligand interactions are concerned, the total hydrogen bonds formed between the two as a function of time are presented in Figure 12d and the number of total interactions between the ligands and each M^pro^ residue (when occurring) are shown in Figure 13. The reference inhibitor N3 appears to be more stable in terms of its hydrogen bonds with the protease, which exhibit only slight deviations from the value of 3. Forming fewer hydrogen bonds but also showing good stability, compound e27 appears to have one hydrogen bond contact with the protease for most of the simulation time. The enzymatic FA derivatives FA rutinoside and raffinose ferulate form significantly more hydrogen bonds. In the case of FA rutinoside, the bonds appear to be more stable and fluctuate around the value of 6 while raffinose ferulate exhibits a more sudden drop after 2 ns of simulation to then be quite stable between 2 and 4 hydrogen bonds. Based on the number and stability of the hydrogen bonds, FA rutinoside shows indications of stronger binding to the protease.

Regarding the overall interactions, as seen in Figure 13a, FA rutinoside interacts with numerous residues around the active site, including multiple contacts with oxyanion hole residues Gly143, Ser144, and Cys145. Moreover, Val42 appears to interact with the ligand. Although it is not calculated as a contacting residue in the molecular docking simulation, it is a neighboring residue to the catalytic histidine (His41). Considering that the contacts are calculated based on the distance of the residue atoms from the atoms of the ligands, it can be difficult to identify and separate individual contacts of the ligand with neighboring residues. However, results show the vicinity of the ligand to this area of the active site. FA rutinoside also interacts with residues located at the S1′ (Thr25, Thr26, and Asn28, which neighbor Leu 27, which is given as a contacting residue), S2 (Ser46, Glu47, Leu50), and S4 subsites (His164, Met165, Leu167). Raffinose ferulate also interacts with oxyanion hole residues, including the catalytic cysteine, and the residues Glu47 and Asp48, which are located near the contacting residues Thr45 and Met49 at the S2 subsite (Figure 13b). Moreover, it interacts with several residues of the S4 subsite, with the ones appearing to form the most interactions being Thr190 and Ala193 (neighbor of the contacting residue Gln192), followed by Glu166. Lastly, compound e27 interacts with similar residues covering all four subsites (Figure 13c). Although it does not appear to interact with any of the catalytic residues, it forms contacts with their neighboring residues Val42, Ser144, and Gly146. Another observation is that the majority of the contacts are with residues located at the S4 subsite (Glu166, Leu167, Thr169, Arg188, Thr190, Gln192, Ala193).

Overall, compared to inhibitor N3 (Figure 13d), it is evident that the ligands examined form more contacts with the protease residues. The fact that the confirmed protease inhibitor N3 exhibits fluctuations in the number of contacts with the various contacting residues over time indicates that such fluctuations are normal. Although the contacts established between N3 and the protease appear to be more stable compared to the rest of the ligands, compound e27 also exhibits a consistent interaction profile. The contacts of FA rutinoside are also quite stable and higher in number overall. Raffinose ferulate, confirming the tendency shown from the RMSD and H-bond diagrams, appears to have the least balance in terms of interactions among the three candidates. In general, the MD simulation leads to the conclusion that the complex of compound e27 is the most stable among the three ligands investigated. However, the results yielded for FA rutinoside are also very encouraging and do not deviate much. This fact, combined with the greater number of interactions with the protease, establishes FA rutinoside as a very interesting candidate for the inhibition of M^pro^.

## 4. Conclusions

The difficulties that have arisen in combating the ongoing pandemic highlight the importance of utilizing any available tool for immunity boosting. In this context, phytochemicals emerge as valuable allies. FA is an abundant bioactive compound, whose derivatization has been investigated both through chemical and enzymatic routes, in search for improved properties. This work studied 54 FA derivatives, among which 14 exhibited similar or better in silico binding affinity to the SARS-CoV-2 M^pro^ compared to confirmed inhibitors. Further computational evaluation of their ADMET properties indicated FA rutinoside, raffinose ferulate, and compound e27 as the most promising hits to be further examined through molecular dynamics simulation. Analysis of the MD trajectories identified FA rutinoside and compound e27 as promising candidates, representing enzymatically and chemically synthesized derivatives, respectively. Several derivatives exhibited good binding affinity to the main protease though, indicating that it would be worth investigating different administration routes in order to overcome the problem of low oral bioavailability, and potential structural modifications that could lead to even higher affinity and more favorable pharmacokinetic properties. Moreover, the favorable results for many enzymatically synthesized derivatives encourage the development and optimization of more sustainable enzymatic processes, which can also include the valorization of biomass in which FA can be found. Even though further in vitro and in vivo research is necessary to provide more reliable data regarding the efficacy of the compounds, this work suggests that FA derivatives are interesting candidates to be considered for their antiviral potential against the current public health concern SARS-CoV-2 and future viral threats.

## Figures and Tables

**Table 1:** Listing and synthesis methods of enzymatically synthesized FA derivatives.

Compound	Reaction	Donor	Acceptor	Enzyme	Solvent System	Yield (Time)	T (°C)	Reference
Methyl ferulate	Esterification	FA	Methanol	CLEAs AnFaeA	Solvent-free (containing buffer)	20.6% (24 h)	30	[71]
	Esterification	Methanol	FA	Immobilized CALB	[bmim]PF6	41.7% (72 h)	60	[72]
Ethyl ferulate	Esterification	FA	Ethanol	CLEAs AnFaeA	Solvent-free (containing buffer)	50.5% (24 h)	30	[71]
	Esterification	Ethanol	FA	*RML*	Hexane	76.2% (72 h)	61	[73]
	Esterification	Ethanol	FA	Immobilized CALB	[bmim]PF6	40.7% (72 h)	60	[72]
Propyl ferulate	Esterification	FA	Propanol	CLEAs AnFaeA	Solvent-free (containing buffer)	98.8% (24 h)	30	[71]
	Esterification	Propanol	FA	Immobilized RML	[bmim]PF6	48.2% (72 h)	60	[72]
	Transesterification	MFA	Propanol	FoFae-II	n-Hexane:1-propanol: water	16% (224 h)	30	[74]
Butyl ferulate	Esterification	FA	Butanol	CLEAs AnFaeA	Solvent-free (containing buffer)	99.5% (24 h)	30	[71]
	Esterification	Butanol	FA	Immobilized RML	[bmim]PF6	52.6% (72 h)	60	[72]
	Esterification	Butanol	FA	Novozym 435	Solvent-free	Traces (15 d)	60	[75]
	Transesterification	MFA	Butanol	AocFaeC	Isooctane: butanol: buffer	n.q.	30	[76]
	Transesterification	MFA	1-Butanol	C1 FAEs immobilized on mesoporous silica	Solvent-free (containing buffer)	n.q.	30	[77]
	Transesterification	MFA	1-Butanol	Depol 740 L	Solvent-free (containing buffer)	Up to 90% (6 d)	37	[78]
	Transesterification	MFA	1-Butanol	CLEAs Ultraflo L	Hexane: 1-butanol: buffer	97% (144 h)	37	[79]
	Transesterification	MFA	1-Butanol	FoFae-I	Hexane:1-butanol: buffer	∼13% (144 h)	35	[80]
Isobutyl ferulate	Esterification	FA	Isobutanol	CLEAs AnFaeA	Solvent-free (containing buffer)	98.4% (24 h)	30	[71]
Pentyl ferulate	Esterification	FA	Pentanol	CLEAs AnFaeA	Solvent-free (containing buffer)	99.4% (24 h)	30	[71]
	Esterification	FA	1-Pentanol	FAEA	CTAB: hexane: pentanol: buffer	60% (n/q)	40	[81]
Isopentyl ferulate	Esterification	FA	Isopentanol	CLEAs AnFaeA	Solvent-free (containing buffer)	97.0% (24 h)	30	[71]
Prenyl ferulate	Transesterification	VFA	Prenol	Fae125	n-Hexane: buffer: DMSO	92.5% (24 h)	25	[82]
	Transesterification	VFA	Prenol	CLEAs Fae125	n-Hexane: buffer	83.7% (34.3 h)	32	[83]
	Transesterification	VFA	Prenol	Fae125	n-Hexane: t-butanol: buffer	81.1% (24 h)	40	[84]
	Transesterification	VFA	Prenol	C1FaeB2	n-Hexane: t-butanol: buffer	71.5% (24 h)	30	[85]
Hexyl ferulate	Esterification	FA	Hexanol	CLEAs AnFaeA	Solvent-free (containing buffer)	98.5% (24 h)	30	[71]
	Esterification	Hexanol	FA	Immobilized RML	[bmim]PF6	38.1% (72 h)	60	[72]
Octyl ferulate	Esterification	FA	Octanol	CLEAs AnFaeA	Solvent-free (containing buffer)	99.3% (24 h)	30	[71]
	Esterification	Octanol	FA	Novozym 435	Solvent-free	93.2% (72 h)	92.2	[86]
	Esterification	Octanol	FA	Immobilized RML	[bmim]PF6	34.9% (72 h)	60	[72]
	Esterification	Octanol	FA	Novozym 435	Solvent free	13% (15 d)	60	[75]
Dodecyl (or lauryl) ferulate	Esterification	FA	Dodecanol	CLEAs AnFaeA	Solvent-free (containing buffer)	96.6% (24 h)	30	[71]
	Esterification	Dodecanol	FA	Novozym 435	Solvent-free	10% (15 d)	60	[75]
Octadecyl (or stearyl) ferulate	Esterification	Octadecanol	FA	Immobilized RML	Hexane	n.q. (72 h)	61	[73]
Oleyl ferulate	Esterification	FA	Oleyl alcohol	CLEAs AnFaeA	Solvent-free (containing buffer)	100% (24 h)	30	[71]
	Transesterification	Oleyl alcohol	FA	Novozym 435	Hexane	99.17% (4 d)	60	[87]
Glyceryl ferulate	Transesterification	Glycerol	EFA	Novozym 435	EMIMTF2N	100% (12 h)	70	[88]
	Esterification	FA	Glycerol	Chirazyme L2 C-2	Solvent-free	80% (>3 h)	80	[89]
	Esterification	FA	Glycerol	FAE-PL	Glycerol: DMSO: buffer	81% (n.q.)	50	[90]
Diglyceryl ferulate	Esterification	FA	Diglycerin S	FAE-PL	Diglycerin S: DMSO: buffer	95% (12 h)	50	[91]
Tocopheryl ferulate	Transesterification	Vitamin E	EFA	Novozym 435	Solvent-free	25.2% (72 h)	60	[92]
Sitosteryl ferulate	Transesterification Esterification	Sitosterol	EFA FA	CRL	Hexane	55% (5 d) 35% (5 d)	63	[73]
	Transesterification	Sitosterol	VFA	CRL	Hexane: 2 butanone	∼55% (10 d)	45	[93]
d-glucose ferulate	Transesterification	VFA	D-glucose	Fae125	n-Hexane: t-butanol: buffer	22.5% (8 h)	45	[94]
d-galactose ferulate	Transesterification	VFA	D-galactose	C1FaeA1	n-Hexane: t-butanol: buffer	22.8% (8 h)	45	[94]
	Esterification	FA	D-galactose	Flavourzyme	Hexane: t-butanol: buffer	41.9% (144 h)	35	[95]
d-mannose ferulate	Transesterification	VFA	D-mannose	C1FaeA1	n-Hexane: t-butanol: buffer	21.5% (8 h)	45	[94]
d-fructose ferulate	Transesterification	VFA	D-fructose	C1FaeA1	n-Hexane: t-butanol: buffer	29.4% (8 h)	45	[94]
Arabinose ferulate	Transesterification	VFA	L-Arabinose	Fae125	n Hexane: buffer: DMSO	56.2% (24 h)	40	[82]
	Transesterification	VFA	L-Arabinose	Fae125	n-Hexane: t-butanol: buffer	33.0%	40	[96]
	Transesterification	VFA	L-Arabinose	C1FaeA1	n-Hexane: t-butanol: buffer	52.2% (8 h)	55	[84]
	Transesterification	VFA	L-arabinose	CLEAs Fae125	n-Hexane: buffer	58.1% (10 h)	32	[83]
	Esterification	FA	D-Arabinose	Multifect P3000	Hexane: t-butanol: buffer	36.7% (144 h)	35	[95]
	Transesterification	MFA	D-arabinose	StFae-C	Hexane: t-butanol: buffer	45% (n.q.)	35	[97]
	Transesterification	MFA EFA	L-arabinose	StFae-C	Hexane: t-butanol: buffer	Up to 50% (120 h) 6.3% (n.q.)	35	[98]
d-xylose ferulate	Transesterification	VFA	D-xylose	C1FaeA1	n-Hexane: t-butanol: buffer	7.5% (8 h)	45	[94]
	Esterification	FA	D-xylose	Multifect P3000	Hexane: t-butanol: buffer	30.8% (144 h)	35	[95]
d-lactose ferulate	Transesterification	VFA	D-lactose	C1FaeA1	n-Hexane: t-butanol: buffer	<2% (8 h)	45	[94]
	Esterification	FA	Lactose	Depol 740 L	n-Hexane: 2-butanone: buffer	4.4% (n.q.)	35	[99]
d-sucrose ferulate	Transesterification	VFA	D-sucrose	FaeA1	n-Hexane: t-butanol: buffer	8.2% (8 h)	45	[94]
	Esterification	FA	D-sucrose	Depol 740 L	n-Hexane: 2-butanone: buffer	13.2% (n.q.)	35	[99]
d-maltose ferulate	Transesterification	VFA	D-maltose	C1FaeA1	n-Hexane: t-butanol: buffer	8.9% (8 h)	45	[94]
d-cellobiose ferulate	Transesterification	VFA	D-cellobiose	C1FaeA1	n-Hexane: t-butanol: buffer	<2% (8 h)	45	[94]
Xylobiose ferulate	Esterification	FA	Xylobiose	Depol 740 L	n-Hexane: 2-butanone: buffer	9.4% (n.q.)	35	[99]
Galactobiose ferulate	Esterification	FA	Galactobiose	Depol 740 L	n-Hexane: 2-butanone: buffer	5.4% (n.q)	35	[99]
Arabinobiose ferulate	Esterification	FA	Arabinobiose	Depol 740 L	n-Hexane: 2-butanone: buffer	7.9% (n.q)	35	[99]
Raffinose ferulate	Esterification	FA	Raffinose	Depol 740 L	n-Hexane: 2-butanone: buffer	11.9% (7 d)	35	[99]
FOS ferulate	Esterification	FA	FOS	Depol 740 L	n-Hexane: 2-butanone: buffer	9.6% (n.q.)	35	[99]
d-mannitol ferulate	Transesterification	VFA	D-mannitol	C1FaeA1	n-Hexane: t-butanol: buffer	26.7% (8 h)	45	[94]
d-sorbitol ferulate	Transesterification	VFA	D-sorbitol	C1FaeA1	n-Hexane: t-butanol: buffer	50.0% (8 h)	45	[94]
d-xylitol ferulate	Transesterification	VFA	D-xylitol	C1FaeA1	n-Hexane: t-butanol: buffer	43.3% (8 h)	45	[94]
FA rutinoside	Transglycosylation	Rutin	FA	Rutinase derived from tartary buckwheat	Buffer	∼4.5 μmol (48 h)	40	[35]

Yields were expressed based on the limiting reactant. FA: Ferulic acid; VFA: Vinyl ferulate; MFA: Methyl ferulate; EFA: Ethyl ferulate; CLEAs: Cross-linked enzyme aggregates; AnFaeA, FAEA: Feruloyl esterase from Aspergillus niger; RML: Lipase from Rhizomucor miehei; FoFae-I, FoFae-II: Feruloyl esterases from Fusarium oxysporum; Novozym 435: Lipase B from C. antarctica immobilized on a microporous acrylic resin (synonym CALB); AocFaeC: Feruloyl esterase from Aspergillus ochraceus; C1FAEs, e.g., C1FaeA1, C1FaeB2: Feruloyl esterases from Myceliophthora thermophila C1; Depol 740 L, Ultraflo L: Commercial multi-enzymatic preparation from Humicola spp. with side feruloyl esterase activity; FAEA: Fae125: Feruloyl esterase from Talaromyces wortmanni; Chirazyme L-2: Immobilized lipase from Candida antarctica; FAE-PL: FAE from Aspergillus niger purified from the commercial preparation ‘Amano’ Pectinase PL; CRL: Lipase from Candida rugosa; Flavourzyme: Commercial multi-enzymatic preparation from Aspergillus oryzae with side feruloyl esterase activity; Multifect P300: Commercial multi-enzymatic preparation from Bacillus amyloliquefaciens with side feruloyl esterase activity; St-FaeC: Feruloyl esterase from Sporotrichum thermophile ATCC 34628; Cetyltrimethylammoniumbromide (CTAB); n.q.: not quantified.

**Table 2:** Molecular docking simulation results for FA and its enzymatically synthesized derivatives.

Compound	Binding Energy (kcal/mol) ^1^	No of Interactions			Total Contacting Residues
		H-Bond ^2^	Hydrophobic ^3^	Pi-Pi	
Ferulic acid	−5.87	3 (ARG 188, THR 190 × 2)	1 (GLN 189)	1 (HIS 41)	HIS 41, MET 49, TYR 54, CYS 145, HIS 164, MET 165, GLU 166, ASP 187, ARG 188, GLN 189, THR 190, GLN 192
Methyl ferulate	−5.73	4 (GLY 143 × 2, SER 144, CYS 145)	1 (MET 165)	1 (HIS 163)	LEU 27, HIS 41, PHE 140, LEU 141, ASN 142, GLY 143, SER 144, CYS 145, HIS 163, MET 165, GLU 166, ARG 188, GLN 189, GLN 192
Ethyl ferulate	−5.87	7 (LEU 141, GLY 143 × 2, SER 144 × 2, CYS 145, GLN 189)	1 (GLN 189)	1 (HIS 163)	LEU 27, HIS 41, PHE 140, LEU 141, ASN 142, GLY 143, SER 144, CYS 145, HIS 163, HIS 164, MET 165, GLU 166, LEU 167, PRO 168, ARG 188, GLN 189, THR 190, GLN 192
Propyl ferulate	−5.98	6 (LEU 141, GLY 143 × 2, SER 144 × 2, CYS 145)	1 (MET 165)	1 (HIS 163)	LEU 27, HIS 41, CYS 44, MET 49, TYR 54, PHE 140, LEU 141, ASN 142, GLY 143, SER 144, CYS 145, HIS 163, HIS 164, MET 165, GLU 166, ASP 187, ARG 188, GLN 189
Butyl ferulate	−6.02	6 (LEU 141, GLY 143 × 2, SER 144 × 2, CYS 145)	1 (MET 165)	1 (HIS 163)	HIS 41, CYS 44, MET 49, PRO 52, TYR 54, PHE 140, LEU 141, ASN 142, GLY 143, SER 144, CYS 145, HIS 163, HIS 164, MET 165, GLU 166, ASP 187, ARG 188, GLN 189
Isobutyl ferulate	−6.30	7 (LEU 141, GLY 143 × 2, SER 144 × 2, CYS 145, GLN 189)	1 (MET 165)	1 (HIS 163)	HIS 41, PHE 140, LEU 141, ASN 142, GLY 143, SER 144, CYS 145, HIS 163, MET 165, GLU 166, LEU 167, PRO 168, ARG 188, GLN 189, THR 190, GLN 192
Pentyl ferulate	−5.92	2 (THR 190, GLN 192)	1 (MET 165)	1 (HIS 41)	HIS 41, MET 49, TYR 54, CYS 145, HIS 164, MET 165, GLU 166, LEU 167, PRO 168, ASP 187, ARG 188, GLN 189, THR 190, GLN 192
Isopentyl ferulate	−6.58	6 (LEU 141, GLY 143 × 2, SER 144 × 2, CYS 145)	1 (MET 49)	1 (HIS 163)	LEU 27, HIS 41, CYS 44, MET 49, PRO 52, TYR 54, PHE 140, LEU 141, ASN 142, GLY 143, SER 144, CYS 145, HIS 163, HIS 164, MET 165, GLU 166, ASP 187, ARG 188, GLN 189
Prenyl ferulate	−6.75	6 (LEU 141, GLY 143 × 2, SER 144 × 2, CYS 145)	1 (GLN 189)	1 (HIS 163)	LEU 27, HIS 41, CYS 44, MET 49, PRO 52, TYR 54, PHE 140, LEU 141, ASN 142, GLY 143, SER 144, CYS 145, HIS 163, HIS 164, MET 165, GLU 166, ASP 187, ARG 188, GLN 189
Hexyl ferulate	−6.47	7 (LEU 141, GLY 143 × 2, SER 144 × 2, CYS 145, GLN 189)	1 (GLN 189)	1 (HIS 163)	HIS 41, MET 49, PRO 52, TYR 54, PHE 140, LEU 141, ASN 142, GLY 143, SER 144, CYS 145, HIS 163, HIS 164, MET 165, GLU 166, HIS 172, ASP 187, ARG 188, GLN 189
Octyl ferulate	−6.20	6 (LEU 141, GLY 143 × 2, SER 144 × 2, CYS 145)	1 (HIS 41)	1 (HIS 163)	HIS 41, MET 49, TYR 54, PHE 140, LEU 141, ASN 142, GLY 143, SER 144, CYS 145, HIS 163, HIS 164, MET 165, GLU 166, ASP 187, ARG 188, GLN 189, THR 190
Dodecyl ferulate	−5.56	3 (ARG 188, THR 190 × 2)	1 (GLU 166)	1 (HIS 41)	HIS 41, MET 49, PHE 140, LEU 141, ASN 142, SER 144, CYS 145, HIS 163, HIS 164, MET 165, GLU 166, HIS 172, PHE 181, ASP 187, ARG 188, GLN 189, THR 190, ALA 191, GLN 192
Octadecyl ferulate	−5.20	4 (ARG 188, THR 190 × 2, GLN 192)	1 (MET 49)	0	THR 25, THR 26, LEU 27, HIS 41, MET 49, PHE 140, LEU 141, ASN 142, GLY 143, SER 144, CYS 145, HIS 163, HIS 164, MET 165, GLU 166, PRO 168, HIS 172, ARG 188, GLN 189, THR 190, ALA 191, GLN 192
Oleyl ferulate	−5.17	4 (ARG 188 × 2, THR 190 × 2)	1 (MET 49)	1 (HIS 41)	THR 24, THR 25, THR 26, LEU 27, HIS 41, MET 49, PHE 140, LEU 141, ASN 142, GLY 143, SER 144, CYS 145, HIS 163, HIS 164, MET 165, GLU 166, PRO 168, HIS 172, ASP 187, ARG 188, GLN 189, THR 190, GLN 192
Glyceryl ferulate	−6.55	12 (LEU 141, GLY 143 × 2, SER 144 × 2, CYS 145, GLU 166, ARG 188, GLN 189, THR 190 × 2, GLN 192)	1 (MET 165)	1 (HIS 163)	LEU 27, HIS 41, PHE 140, LEU 141, ASN 142, GLY 143, SER 144, CYS 145, HIS 163, MET 165, GLU 166, LEU 167, PRO 168, ARG 188, GLN 189, THR 190, GLN 192
Diglyceryl ferulate	−6.61	10 (TYR 54, LEU 141, GLY 143 × 2, SER 144 × 2, CYS 145, HIS 164, ARG 188, GLN 189)	1 (GLN 189)	1 (HIS 163)	HIS 41, CYS 44, MET 49, PRO 52, TYR 54, PHE 140, LEU 141, ASN 142, GLY 143, SER 144, CYS 145, HIS 163, HIS 164, MET 165, GLU 166, HIS 172, ASP 187, ARG 188, GLN 189, THR 190, GLN 192
Tocopheryl ferulate	−6.91	2 (SER 46, GLN 189)	1 (GLN 189)		THR 25, LEU 27, HIS 41, SER 46, GLU 47, MET 49, LEU 50, TYR 54, PHE 140, LEU 141, ASN 142, GLY 143, SER 144, CYS 145, HIS 163, HIS 164, MET 165, GLU 166, PRO 168, HIS 172, ASP 187, ARG 188, GLN 189, THR 190
Sitosteryl ferulate	−7.81	3 (THR 25, HIS 41, GLY 143)	1 (PRO 168)	1 (HIS 41)	THR 24, THR 25, THR 26, LEU 27, HIS 41, CYS 44, THR 45, SER 46, MET 49, LEU 141, ASN 142, GLY 143, SER 144, CYS 145, HIS 164, MET 165, GLU 166, LEU 167, PRO 168, ARG 188, GLN 189, THR 190, ALA 191, GLN 192
d-glucose ferulate	−7.09	11 (LEU 141, SER 144, HIS 163, GLU 166, ARG 188, GLN 189, THR 190 × 3, GLN 192 × 2)	1 (GLU 166)	1 (HIS 163)	HIS 41, PHE 140, LEU 141, ASN 142, SER 144, CYS 145, HIS 163, HIS 164, MET 165, GLU 166, LEU 167, PRO 168, ARG 188, GLN 189, THR 190, ALA 191, GLN 192
d-galactose ferulate	−7.24	13 (LEU 141 × 2, ASN 142, GLY 143, SER 144 × 3, CYS 145, HIS 163, ARG 188, THR 190 × 2, GLN 192)	1 (GLN 189)	0	PHE 140, LEU 141, ASN 142, GLY 143, SER 144, CYS 145, HIS 163, MET 165, GLU 166, PRO 168, ASP 187, ARG 188, GLN 189, THR 190, ALA 191, GLN 192
d-mannose ferulate	−7.09	11 (LEU 141, SER 144, HIS 163, GLU 166, ARG 188, GLN 189, THR 190 × 3, GLN 192 × 2)	1 (GLU 166)	1 (HIS 163)	HIS 41, PHE 140, LEU 141, ASN 142, SER 144, CYS 145, HIS 163, HIS 164, MET 165, GLU 166, LEU 167, PRO 168, ARG 188, GLN 189, THR 190, ALA 191, GLN 192
d-fructose ferulate	−7.06	8 (THR 26 × 2, PHE 140, LEU 141, ASN 142, SER 144, HIS 163, GLU 166)	1 (HIS 41)	1 (HIS 41)	THR 24, THR 25, THR 26, LEU 27, HIS 41, THR 45, MET 49, PHE 140, LEU 141, ASN 142, GLY 143, SER 144, CYS 145, HIS 163, HIS 164, MET 165, GLU 166, HIS 172, GLN 189
l-arabinose ferulate	−7.08	5 (HIS 41, MET 49, HIS 164, GLN 189, THR 190)	1 (LEU 167)	0	HIS 41, MET 49, TYR 54, CYS 145, HIS 164, MET 165, GLU 166, LEU 167, PRO 168, ASP 187, ARG 188, GLN 189, THR 190, ALA 191, GLN 192
d-xylose ferulate	−7.37	13 (LEU 141 × 2, ASN 142, GLY 143, SER 144 × 3, CYS 145, HIS 163, ARG 188, THR 190 × 2, GLN 192)	1 (ΜΕΤ 165)	1 (HIS 163)	PHE 140, LEU 141, ASN 142, GLY 143, SER 144, CYS 145, HIS 163, HIS 164, MET 165, GLU 166, PRO 168, ASP 187, ARG 188, GLN 189, THR 190, GLN 192
d-lactose ferulate	−7.63	12 (ASN 142 × 2, GLY 143, GLU 166 × 3, ARG 188, GLN 189, THR 190 × 3, GLN 192)	1 (ΜΕΤ 165)	1 (HIS 41)	HIS 41, MET 49, LEU 141, ASN 142, GLY 143, SER 144, CYS 145, HIS 163, HIS 164, MET 165, GLU 166, LEU 167, PRO 168, ASP 187, ARG 188, GLN 189, THR 190, ALA 191, GLN 192
d-sucrose ferulate	−7.77	14 (THR 26, LEU 141 × 2, GLY 143 × 2, SER 144 × 3, CYS 145, HIS 163 × 2, GLU 166, GLN 189 × 2)	1 (GLN 189)	0	THR 25, THR 26, LEU 27, HIS 41, MET 49, PHE 140, LEU 141, ASN 142, GLY 143, SER 144, CYS 145, HIS 163, HIS 164, MET 165, GLU 166, LEU 167, PRO 168, HIS 172, GLN 189, THR 190, ALA 191, GLN 192
d-maltose ferulate	−7.47	13 (SER 46 × 2, LEU 141 × 2, ASN 142, GLY 143 × 2, SER 144 × 2, CYS 145, GLU 166, GLN 189 × 2)	1 (THR 25)	0	THR 24, THR 25, THR 26, LEU 27, HIS 41, THR 45, SER 46, MET 49, PHE 140, LEU 141, ASN 142, GLY 143, SER 144, CYS 145, HIS 163, HIS 164, MET 165, GLU 166, GLN 189
d-cellobiose ferulate	−7.50	9 (THR 24, LEU 141, ASN 142 × 2, GLY 143 × 2, SER 144, THR 190, GLN 192)	1 (ΜΕΤ 165)	0	THR 24, THR 25, THR 26, LEU 27, HIS 41, MET 49, PHE 140, LEU 141, ASN 142, GLY 143, SER 144, CYS 145, HIS 163, HIS 164, MET 165, GLU 166, LEU 167, PRO 168, ARG 188, GLN 189, THR 190, ALA 191, GLN 192
Xylobiose ferulate	−7.97	11 (THR 24 × 2, THR 25, THR 26, THR 45 × 2, THR 46 × 2, HIS 41, PHE 140, GLU 166)	1 (GLU 166)	1 (HIS 163)	THR 24, THR 25, THR 26, LEU 27, HIS 41, THR 45, SER 46, MET 49, PHE 140, LEU 141, GLY 143, SER 144, CYS 145, HIS 163, HIS 164, MET 165, GLU 166, HIS 172, GLN 189
Galactobiose ferulate	−8.36	12 (ASN 119 × 2, PHE 140, LEU 141, GLY 143, SER 144 × 2, CYS 145, GLU 166 × 2, GLN 189, THR 190)	1 (GLN 189)	0	GLN 19, THR 25, THR 26, LEU 27, HIS 41, MET 49, TYR 118, ASN 119, PHE 140, LEU 141, ASN 142, GLY 143, SER 144, CYS 145, HIS 163, HIS 164, MET 165, GLU 166, LEU 167, PRO 168, HIS 172, GLN 189, THR 190, ALA 191, GLN 192
Arabinobiose ferulate	−7.88	7 (THR 26, PHE 140, ASN 142, GLU 166 × 2, LEU 167, GLN 189)	1 (GLN 189)	1 (HIS 41)	THR 24, THR 25, THR 26, LEU 27, HIS 41, MET 49, PRO 52, TYR 54, PHE 140, LEU 141, ASN 142, GLY 143, SER 144, CYS 145, HIS 164, MET 165, GLU 166, LEU 167, PRO 168, HIS 172, ASP 187, ARG 188, GLN 189, THR 190, GLN 192
Raffinose ferulate	−8.34	8 (THR 26, SER 144 × 2, CYS 145, HIS 163, GLU 166, THR 190, GLN 192)	1 (MET 49)	0	THR 24, THR 25, THR 26, LEU 27, THR 45, MET 49, PHE 140, LEU 141, ASN 142, GLY 143, SER 144, CYS 145, HIS 163, HIS 164, MET 165, GLU 166, LEU 167, PRO 168, HIS 172, ARG 188, GLN 189, THR 190, ALA 191, GLN 192
FOS ferulate 1	−8.18	9 (SER 46 × 2, PHE 140, LEU 141 × 2, GLY 143, SER 144, HIS 163, THR 190)	1 (GLN 189)	0	THR 25, THR 26, HIS 41, THR 45, SER 46, MET 49, PHE 140, LEU 141, ASN 142, GLY 143, SER 144, CYS 145, HIS 163, HIS 164, MET 165, GLU 166, LEU 167, PRO 168, ARG 188, GLN 189, THR 190, ALA 191, GLN 192
FOS ferulate 2	−7.34	8 (THR 26 × 3, GLY 143 × 2, HIS 164, GLU 166, THR 190)	1 (LEU 27)	0	THR 25, THR 26, LEU 27, HIS 41, VAL 42, MET 49, PHE 140, LEU 141, ASN 142, GLY 143, SER 144, CYS 145, HIS 163, HIS 164, MET 165, GLU 166, LEU 167, PRO 168, ARG 188, GLN 189, THR 190, GLN 192
FOS ferulate 3	−8.52	14 (SER 46 × 3, LEU 141 × 2, ASN 142, GLY 143 × 2, SER 144 × 2, CYS 145, HIS 163, HIS 164, GLN 192)	1 (LEU 50)	0	THR 24, THR 25, THR 26, LEU 27, HIS 41, CYS 44, THR 45, SER 46, GLU 47, MET 49, LEU 50, PRO 52, TYR 54, PHE 140, LEU 141, ASN 142, GLY 143, SER 144, CYS 145, HIS 163, HIS 164, MET 165, GLU 166, LEU 167, PRO 168, HIS 172, ASP 187, ARG 188, GLN 189, THR 190, ALA 191, GLN 192
d-mannitol ferulate	−6.38	12 (LEU 141, GLY 143 × 2, SER 144 × 2, CYS 145, GLU 166, ARG 188, GLN 189, THR 190 × 2, GLN 192)	1 (ΜΕΤ 165)	1 (HIS 163)	PHE 140, LEU 141, ASN 142, GLY 143, SER 144, CYS 145, HIS 163, MET 165, GLU 166, LEU 167, PRO 168, HIS 172, ARG 188, GLN 189, THR 190, ALA 191, GLN 192
d-sorbitol ferulate	−6.43	13 (LEU 141, GLY 143 × 2, SER 144 × 2, CYS 145, GLU 166, ARG 188, GLN 189 × 2, THR 190 × 2, GLN 192)	1 (ΜΕΤ 165)	1 (HIS 163)	PHE 140, LEU 141, ASN 142, GLY 143, SER 144, CYS 145, HIS 163, HIS 164, MET 165, GLU 166, LEU 167, PRO 168, ARG 188, GLN 189, THR 190, ALA 191, GLN 192
d-xylitol ferulate	−6.68	15 (PHE 140, LEU 141 × 2, ASN 142, GLY 143, SER 144 × 3, CYS 145, HIS 163, GLU 166, ARG 188, THR 190 × 2, GLN 192)	1 (GLN 189)	0	PHE 140, LEU 141, ASN 142, GLY 143, SER 144, CYS 145, HIS 163, MET 165, GLU 166, PRO 168, HIS 172, ASP 187, ARG 188, GLN 189, THR 190, GLN 192
FA rutinoside	−8.40	16 (THR 24 × 3, THR 25, THR 45, LEU 141 × 2, GLY 143 × 3, SER 144 × 2, CYS 145, HIS 163, GLU 166, ARG 188, GLN 189)	1 (MET 49)	0	THR 24, THR 25, THR 26, LEU 27, HIS 41, CYS 44, THR 45, SER 46, MET 49, PHE 140, LEU 141, ASN 142, GLY 143, SER 144, CYS 145, HIS 163, HIS 164, MET 165, GLU 166, HIS 172, ARG 188, GLN 189, THR 190, GLN 192
**N3**	−8.26	4 (CYS 145, GLU 166, GLN 189)	1 (MET 49)	1(HIS 41)	THR 25, LEU 27, HIS 41, MET 49, LEU 50, TYR 54, PHE 140, LEU 141, ASN 142, GLY 143, SER 144, CYS 145, HIS 163, HIS 164, MET 165, GLU 166, LEU 167, PRO 168, HIS 172, ASP 187, ARG 188, GLN 189, THR 190, ALA 191, GLN 192
**GC376**	−7.80	5 (HIS 41, PHE 140, HIS 163, GLU 166, GLN 189)	1 (ASP 187)	0	HIS 41, MET 49, TYR 54, PHE 140, LEU 141, ASN 142, GLY 143, SER 144, CYS 145, HIS 163, HIS 164, MET 165, GLU 166, LEU 167, PRO 168, HIS 172, ASP 187, ARG 188, GLN 189, THR 190, ALA 191, GLN 192

^1^: The binding energies are given as negative values and correspond to the best cluster for each compound. A lower binding energy corresponds to a higher binding affinity. ^2^: H-bonds were calculated by Pymol. ^3^: Hydrophobic and pi-pi interactions were calculated by the YASARA structure.

**Table 3:** Listing and synthesis method of chemically synthesized FA derivatives.

Category	Code ^1^	Name	Derivatization Method	Yield	NoD ^2^	Reference
FA amide	**7a**	Not given	Six steps: acetylation with acetic anhydride in aqueous sodium hydroxide solution, reaction with thionyl chloride, reaction with a substituted 2-amino-1-phenylethanone, reduction to the corresponding alcohol with sodium borohydride and hydrolyzation of the acetyl group with NaOH, alkylation of the phenolic hydroxyl group, and alkylation with a bromoalkane in the presence of NaH.	Not given	16	[32]
Hydrogenated FA amides (A)	**13b**	Not given	Four steps: catalytic hydrogenation of FA using Pd/C and H2 in the presence of HCl, multi-step reaction involving an acid chloride intermediate (including hydrolysis, hydroxyl protection, acyl chloride formation, amidation, and deprotection) and microwave radiation, alkylation of the phenolic hydroxyl group with bromoalkene and NaOH, reaction with bromoalkane, and deprotonation with NaOH.	71%	9	[32]
Hydrogenated FA amides (B)	**4f**	N-(2-(4-(Benzyloxy)phenyl)-2-(prop-2-yn-1-yloxy)ethyl)-3-(3,4-dimethoxyphenyl)propanamide	Three steps: catalytic hydrogenation of FA with Pd/C and H2 towards ethyl ferulate, substitution with 2-amino-1-phenylethanol under microwave radiation at 130 oC, alkylation with bromoalkane.	68%	7	[103]
FA sulfonamide	**2**	€-3-(4-(2-((4-acetamidophenyl)sulfonamido)ethoxy)-3-methoxyphenyl)acrylate methyl	Two parallel steps: reaction of FA with alcohol catalyzed by sulfuric acid, and reaction of sulfonyl chloride and bromoethylamine hydrobromide in dichloromethane in the presence of triethylamine. Dissolvation of intermediate A in acetonitrile and potassium carbonate and combination with the other intermediate towards the target compound.	45%	16	[33]
α,β-Unsaturated amide derivatives of FA with an α-aminophosphonate moiety	**g18**	(E)-((4-chlorophenyl)(3-(3-methoxy-4-((4-(trifluoromethyl)benzyl)oxy)phenyl)acrylamido)methyl)phosphonate	Two parallel routes towards two intermediates, which are then combined via dehydration condensation reaction towards the final product. The first intermediate involves treatment of an aromatic aldehyde with ammonia, reaction with diethyl phosphite, and then hydrolysis to diethyl 1-aminoarylmethylphosphonate. The second intermediate is also produced in three steps, starting from FA, which is methylated in with methyl alcohol in the presence of sulfuric acid, then esterification with benzyl halide in the presence of potassium carbonate and acetonitrile, and then hydrolyzation with NaOH.	53.60%	26	[104]
FA derivatives with a quinazoline moiety (A)	**e27**	2-methoxyphen€(E)-3-(3-methoxy-4-((4-oxoquinazolin-3(4H)-yl)methoxy)phenyl)acrylate	The first step of the derivatization involves esterification of FA, either with the appropriate alcohol in the presence of sulfuric acid, or by reaction with acetic anhydride, NaOH, and then with thionyl chloride towards an O-acetyl ferulic acid chloride, and finally mixture with tetrahydrofuran, triethylamine, and the appropriate phenol. Then, the intermediate was mixed with 3-chloromethyl-4(3H)-quinazolinone, potassium carbonate, potassium iodide, and acetronitrile toward the final product.	50.80%	28	[105]
FA derivatives with a quinazoline moiety (B)	**e28**	4-allyl-2-methoxyp€yl-(E)-3-(3-methoxy-4-((4-oxoquinazolin-3(4H)-yl)methoxy)phenyl)acrylate	The first step of the derivatization involves esterification of FA, either with the appropriate alcohol in the presence of sulfuric acid, or by reaction with acetic anhydride, NaOH, and then with thionyl chloride towards an O-acetyl ferulic acid chloride, and finally mixture with tetrahydrofuran, triethylamine, and the appropriate phenol. Then, the intermediate was mixed with 3-chloromethyl-4(3H)-quinazolinone, potassium carbonate, potassium iodide, and acetronitrile toward the final product.	64.80%	28	[105]
FA amide of 3-aminomethyl glaucine	**5**	Feruloyl amide of 3-aminomethylglaucine	Peptide chemistry methods using EDC/HOBt to link 3-aminomethylglaucine to FA. 3-aminomethylglaucine was produced from glaucine through reaction with N-(hydroxymethyl)acetamide in acidic media and subsequent hydrolyzation.	61.60%	1	[106]
Myricetin derivatives with a FA amide scaffold	**4n**	(E)-N-(4-bromophenyl)-3-(4-(3-((5,7-dimethoxy-4-oxo-2-(3,4,5-trimethoxyphenyl)-4Hchromen-3-yl)oxy)propoxy)-3-methoxyphenyl)acrylamide	Synthesis of two intermediates, which are then combined using DMF and potassium carbonate. The first one is derived from FA, involving reaction with acetic anhydride in the presence of NaOH, then amidation through a reaction with a phenylamine in the presence of HOBt and EDCl and lastly dissolution in acetonitrile and hydrazine hydrate. The second is a myricitrin derivative occurring from reaction with DMF, potassium carbonate, and methyl iodide in the presence of hydrochloride and then with DMF and dibromoalkanes.	67.92%	22	[107]
FA derivatives containing dithioacetal moiety (A)	**2a**	4-(bis((2-Hydroxyethyl)thio)methyl)-2-methoxyphenyl(E)-3-(4-acetoxy-3-methoxyphenyl)acrylate	Reaction of FA with acetic anhydride and NaOH towards O-acetyl FA, then reaction with thionyl chloride and mixing of the respective chloride with 1,2 dioxane, triethylamine, and hydroxy aldehyde. The synthesized intermediate was mixed with thiol, NaHSO_4_·SiO_2_, and dichloromethane towards the final product.	69.30%	17	[34]
FA derivatives containing dithioacetal moiety (B)	**2y**	Not given	Reaction of FA with acetic anhydride and NaOH towards O-acetyl FA, then reaction with thionyl chloride and mixing of the respective chloride with 1,2 dioxane, triethylamine, and hydroxy aldehyde. The synthesized intermediate was mixed with thiol, NaHSO_4_·SiO_2_, and dichloromethane towards the final product.	Not given	8	[34]
FA derivatives containing dithioacetal moiety (C)	**2s**	Not given	Reaction of FA with acetic anhydride and NaOH towards O-acetyl FA, then reaction with thionyl chloride and mixing of the respective chloride with 1,2 dioxane, triethylamine, and hydroxy aldehyde. The synthesized intermediate was mixed with thiol, NaHSO_4_·SiO_2_, and dichloromethane towards the final product.	Not given	2	[34]
Trans-FA esters with a chalcone group	**F3**	(E)-methyl-3-(4-(2-(4-((E)-3-(2-fluorophenyl)acryloyl)phenoxy)ethoxy)-3-methoxyphenyl)acrylate	Esterification of FA with the appropriate alcohol in the presence of sulfuric acid and reaction with potassium carbonate in butanone with the addition of 1,2, dibromoethane towards an intermediate compound. Reaction of 4-hydroxyacetophenone with the appropriate aromatic aldehyde and reaction of the product with the previously mentioned intermediate and potassium carbonate in dimethylformamide.	92.60%	35	[108]
Trans-Fa derivatives containing acylhydrazone moiety	**D4**	(E)-3-(4-(benzyloxy)-3-methoxyphenyl)-N’-(thiophen-2-ylmethylene)acrylohydrazide	Starting with trans-ferulic acid, through four steps, including substitution, using RX in potassium carbonate and dimethylformamide, hydrolysis, using KOH and methanol, hydrazinoly-sis, using hydroxybenzotriazole (HoBt/EDCl) in DMF, and condensation, involving an appropriate aldehyde and methanol.	80.80%	23	[109]
DiFA		Diferulic acid	Fractionation of dehydrogenated polymers of FA by ultrafiltration (synthesized with horseradish peroxidase from FA).	Not given	n.d.	[110]
TriFA		Triferulic acid	Fractionation of dehydrogenated polymers of FA by ultrafiltration (synthesized with horseradish peroxidase from FA).	Not given	n.d.	[110]
FA 3-amino derivatives	**MY3**	(E)-ethyl 3-(4-isopropoxy-3-methoxy-5-nitrophenyl)acrylate	Nitration of FA with acetic and nitric acid, esterification of the product with the corresponding alcohol in sulfuric acid, and mixture of the derived compound with sodium carbonate in DMF. Addition of the corresponding alkyl bromide and tetra-butylammonium iodide in DMF to the previously described solution to yield the final product.	9%	23	[111]

^1^: Name of the compound as it is coded in the respective publication; ^2^: Number of FA derivatives synthesized in the respective study, among which the ones used in this work were selected.

**Table 4:** Molecular docking simulation results for the chemically synthesized derivatives of FA.

Compound	Binding Energy ^1^ (kcal/mol)	No of Interactions			Total Contacting Residues
		H-Bond ^2^	Hydrophobic ^3^	Pi-Pi	
**7a**	−6.67	1 (HIS 41)	1 (MET 165)	1 (HIS 41)	THR 25, THR 26, LEU 27, HIS 41, MET 49, LEU 141, ASN 142, GLY 143, CYS 145, HIS 164, MET 165, GLU 166, LEU 167, PRO 168, ARG 188, GLN 189, THR 190, ALA 191, GLN 192
**13b**	−6.54	0	1 (GLN 189)	0	THR 26, LEU 27, HIS 41, MET 49, PHE 140, LEU 141, ASN 142, GLY 143, SER 144, CYS 145, HIS 163, HIS 164, MET 165, GLU 166, LEU 167, PRO 168, HIS 172, ASP 187, ARG 188, GLN 189, THR 190, ALA 191, GLN 192
**4f**	−6.27	3 (ASN 142, HIS 163, GLN 189)	1 (GLN 189)	1 (HIS 41)	THR 25, THR 26, LEU 27, HIS 41, VAL 42, MET 49, PHE 140, LEU 141, ASN 142, GLY 143, SER 144, CYS 145, HIS 163, HIS 164, MET 165, GLU 166, HIS 172, ASP 187, ARG 188, GLN 189
**2**	−7.72	3 (THR 25, HIS 41, GLN 192)	1 (THR 25)	0	THR 24, THR 25, THR 26, LEU 27, HIS 41, THR 45, MET 49, LEU 141, ASN 142, GLY 143, SER 144, CYS 145, HIS 164, MET 165, GLU 166, LEU 167, PRO 168, ARG 188, GLN 189, THR 190, GLN 192
**g18**	−6.91	0	1 (GLY 143)	1 (HIS 41)	THR 24, THR 25, THR 26, LEU 27, HIS 41, MET 49, ASN 119, LEU 141, ASN 142, GLY 143, SER 144, CYS 145, HIS 163, HIS 164, MET 165, GLU 166, LEU 167, PRO 168, HIS 172, ARG 188, GLN 189, THR 190, ALA 191, GLN 192
**e27**	−8.11	2 (HIS 41, GLN 192)	1 (GLN 189)	1 (HIS 41)	THR 24, THR 25, THR 26, LEU 27, HIS 41, CYS 44, MET 49, PRO 52, TYR 54, GLY 143, CYS 145, HIS 164, MET 165, GLU 166, LEU 167, PRO 168, ASP 187, ARG 188, GLN 189, THR 190, GLN 192
**e28**	−7.76	2 (HIS 41, GLN 192)	1 (GLN 189)	1 (HIS 41)	THR 25, THR 26, LEU 27, HIS 41, CYS 44, MET 49, PRO 52, TYR 54, GLY 143, CYS 145, HIS 164, MET 165, GLU 166, LEU 167, PRO 168, ASP 187, ARG 188, GLN 189, THR 190, GLN 192
**5**	−7.16	4 (THR 26, GLY 143, SER 144, CYS 145)	1 (MET 49)	1 (HIS 41)	THR 25, THR 26, LEU 27, HIS 41, MET 49, PHE 140, LEU 141, ASN 142, GLY 143, SER 144, CYS 145, HIS 163, HIS 164, MET 165, GLU 166, PRO 168, HIS 172, ARG 188, GLN 189, THR 190, GLN 192
**4n**	−7.82	2 (LEU 141, GLY 143)	1 (MET 165)	0	LEU 27, HIS 41, SER 46, MET 49, TYR 54, PHE 140, LEU 141, ASN 142, GLY 143, SER 144, CYS 145, HIS 163, HIS 164, MET 165, GLU 166, PRO 168, HIS 172, ASP 187, ARG 188, GLN 189, THR 190, ALA 191
**2a**	−6.52	10 (THR 24, THR 45, THR 46 × 2, GLY 143, GLU 166, ARG 188, THR 190 × 2, GLN 192)	1 (THR 25)	0	THR 24, THR 25, THR 26, HIS 41, CYS 44, THR 45, SER 46, MET 49, ASN 142, GLY 143, SER 144, CYS 145, HIS 164, MET 165, GLU 166, LEU 167, PRO 168, ARG 188, GLN 189, THR 190, GLN 192
**2y**	−6.54	11 (HIS 41, THR 45, SER 46 × 3, LEU 141, ASN 142, GLY 143, SER 144 × 2, CYS 145)	1 (HIS 41)	1 (HIS 41)	THR 24, THR 25, THR 26, LEU 27, HIS 41, THR 45, SER 46, MET 49, PHE 140, LEU 141, ASN 142, GLY 143, SER 144, CYS 145, HIS 163, HIS 164, MET 165, GLU 166, GLN 189
**2s**	−6.67	0	1 (MET 165)	1 (HIS 41)	THR 24, THR 25, THR 26, LEU 27, HIS 41, MET 49, TYR 54, ASN 142, GLY 143, CYS 145, HIS 164, MET 165, ASP 187, ARG 188, GLN 189
**F3**	−7.80	5 (THR 24, THR 25, THR 45 × 2, SER 46)	1 (MET 165)	2 (HIS 41 × 2)	THR 24, THR 25, THR 26, LEU 27, HIS 41, THR 45, SER 46, MET 49, LEU 141, ASN 142, GLY 143, SER 144, CYS 145, HIS 164, MET 165, GLU 166, LEU 167, PRO 168, ARG 188, GLN 189, THR 190
**D4**	−6.89	0	1 (MET 165)	1 (HIS 41)	THR 25, THR 26, LEU 27, HIS 41, VAL 42, MET 49, LEU 50, PHE 140, LEU 141, ASN 142, GLY 143, SER 144, CYS 145, HIS 163, HIS 164, MET 165, GLU 166, PRO 168, HIS 172, GLN 189, THR 190, ALA 191
**diFA**	−7.64	0	1 (HIS 41)	1 (HIS 41)	THR 24, THR 25, THR 26, LEU 27, HIS 41, CYS 44, THR 45, SER 46, MET 49, TYR 54, LEU 141, ASN 142, GLY 143, SER 144, CYS 145, HIS 164, MET 165, GLU 166, ASP 187, ARG 188, GLN 189
**triFA**	−8.32	4 (THR 26, TYR 54 × 2, ASP 187)	1 (MET 165)	0	THR 24, THR 25, THR 26, LEU 27, HIS 41, CYS 44, MET 49, PRO 52, TYR 54, PHE 140, LEU 141, ASN 142, GLY 143, SER 144, CYS 145, HIS 163, HIS 164, MET 165, GLU 166, LEU 167, PRO 168, ASP 187, ARG 188, GLN 189, THR 190, GLN 192
**MY3**	−6.29	4 (GLY 143, SER 144 × 2, GLN 189)	1 (MET 165)	0	HIS 41, MET 49, PHE 140, LEU 141, ASN 142, GLY 143, SER 144, CYS 145, HIS 163, HIS 164, MET 165, GLU 166, HIS 172, PHE 181, VAL 186, ASP 187, ARG 188, GLN 189
**N3**	−8.26	4 (CYS 145, GLU 166, GLN 189)	1 (MET 49)	1(HIS 41)	THR 25, LEU 27, HIS 41, MET 49, LEU 50, TYR 54, PHE 140, LEU 141, ASN 142, GLY 143, SER 144, CYS 145, HIS 163, HIS 164, MET 165, GLU 166, LEU 167, PRO 168, HIS 172, ASP 187, ARG 188, GLN 189, THR 190, ALA 191, GLN 192
**GC376**	−7.80	5 (HIS 41, PHE 140, HIS 163, GLU 166, GLN 189)	1 (ASP 187)	0	HIS 41, MET 49, TYR 54, PHE 140, LEU 141, ASN 142, GLY 143, SER 144, CYS 145, HIS 163, HIS 164, MET 165, GLU 166, LEU 167, PRO 168, HIS 172, ASP 187, ARG 188, GLN 189, THR 190, ALA 191, GLN 192

^1^: The binding energies are given as negative values and correspond to the best cluster for each compound. A lower binding energy corresponds to a higher binding affinity. ^2^: H-bonds were calculated by Pymol. ^3^: Hydrophobic and pi-pi interactions were calculated by the YASARA structure.

**Table 5:** Physicochemical properties, lipophilicity, solubility, bioavailability, and druglikeness predictions for FA and its selected derivatives.

	Physicochemical Properties								Lipophilicity/Solubility		Bioavailability and Druglikeness		
Compound Name	Formula	MW ^1^ (g/mol)	RB ^2^	HBA ^3^	HBD ^4^	Fraction C sp^3 5^	TPSA ^6^ (Å²)	pKa of Most Basic/Acidic Group ^7^	Log P_o/w_ ^8^	LogS ^9^	Lipinski ^10^	Bioavailability Score ^11^	Druglikeness Score ^12^
FA	C_10_H_10_O_4_	194.18	3	4	2	0.1	66.76	<0./4.54	1.51	−2.11	Yes (0)	0.85	−0.61
Methyl ferulate	C_11_H_12_O_4_	208.21	4	4	1	0.18	55.76	<0./9.69	1.84	−2.32	Yes (0)	0.55	−0.76
Ethyl ferulate	C_12_H_14_O_4_	222.24	5	4	1	0.25	55.76	<0./9.69	2.2	−2.55	Yes (0)	0.55	−0.55
Propyl ferulate	C_13_H_16_O_4_	236.26	6	4	1	0.31	55.76	<0./9.69	2.73	−2.89	Yes (0)	0.55	−0.34
Butyl ferulate	C_14_H_18_O_4_	250.29	7	4	1	0.36	55.76	<0./9.69	3.09	−3.12	Yes (0)	0.55	−0.42
Isobutyl ferulate	C_14_H_18_O_4_	250.29	6	4	1	0.36	55.76	<0./9.69	3.17	−3.24	Yes (0)	0.55	−0.1
Pentyl ferulate	C_15_H_20_O_4_	264.32	8	4	1	0.4	55.76	<0./9.69	3.63	−3.47	Yes (0)	0.55	−0.51
Isopentyl ferulate	C_15_H_20_O_4_	264.32	7	4	1	0.4	55.76	<0./9.69	3.52	−3.47	Yes (0)	0.55	−0.01
Prenyl ferulate	C_15_H_18_O_4_	262.3	6	4	1	0.27	55.76	<0./9.69	3.34	−3.41	Yes (0)	0.55	−0.49
Hexyl ferulate	C_16_H_22_O_4_	278.34	9	4	1	0.44	55.76	<0./9.69	4.17	−3.82	Yes (0)	0.55	−0.51
Octyl ferulate	C_18_H_26_O_4_	306.4	11	4	1	0.5	55.76	<0./9.69	5.25	−4.52	Yes (0)	0.55	−0.51
Dodecyl ferulate	C_22_H_34_O_4_	362.5	15	4	1	0.59	55.76	<0./9.69	7.42	−5.94	Yes (0)	0.55	−0.51
Octadecyl ferulate	C_28_H_46_O_4_	446.66	21	4	1	0.68	55.76	<0./9.68	10.67	−8.08	Yes; (1, logP)	0.55	−0.51
Oleyl ferulate	C_28_H_44_O_4_	444.65	20	4	1	0.61	55.76	<0./9.69	9.74	−7.55	Yes (1, logP)	0.55	−0.45
Glyceryl ferulate	C_13_H_16_O_6_	268.26	7	6	3	0.31	96.22	<0./9.69	0.53	−1.61	Yes (0)	0.55	−0.05
Diglyceryl ferulate	C_16_H_22_O_8_	342.34	11	8	4	0.44	125.68	<0./9.70	−0.23	−1.28	Yes (0)	0.55	−0.11
Tocopheryl ferulate	C_39_H_58_O_5_	606.87	17	5	1	0.62	64.99	<0./9.69	12.51	−10.56	No (2, MW, logP)	0.17	1.14
Sitosteryl ferulate	C_39_H_58_O_4_	590.88	11	4	1	0.72	55.76	<0./9.69	11.61	−10.19	No (2, MW, logP)	0.17	1.21
D-glucose ferulate	C_16_H_20_O_9_	356.32	6	9	5	0.44	145.91	<0./9.69	−0.87	−1.28	Yes (0)	0.55	−0.15
D-galactose ferulate	C_16_H_20_O_9_	356.32	6	9	5	0.44	145.91	<0./9.69	−0.87	−1.28	Yes (0)	0.55	−0.15
D-mannose ferulate	C_16_H_20_O_9_	356.32	6	9	5	0.44	145.91	<0./9.69	−0.87	−1.28	Yes (0)	0.55	−0.15
D-fructose ferulate	C_16_H_20_O_9_	356.32	7	9	5	0.44	145.91	<0./9.69	−0.56	−1.41	Yes (0)	0.55	−0.24
L-arabinose ferulate	C_15_H_18_O_8_	326.3	6	8	4	0.4	125.68	<0./9.69	−0.25	−1.5	Yes (0)	0.55	−0.18
D-xylose ferulate	C_15_H_18_O_8_	326.3	6	8	4	0.4	125.68	<0./9.69	−0.25	−1.5	Yes (0)	0.55	−0.18
FOS ferulate 1	C_26_H_28_O_12_	532.49	11	12	5	0.31	181.44	<0./9.69	1.71	−3.73	No (2; MW, HBD)	0.17	0.58
D-lactose ferulate	C_21_H_28_O_15_	520.44	9	15	8	0.57	234.29	<0./9.69	−2.87	−0.79	No (3; MW, HBD, HBA)	0.17	−0.02
D-sucrose ferulate	C_22_H_30_O_14_	518.47	10	14	8	0.59	225.06	<0./9.69	−1.98	−1.27	No (3; MW, HBD, HBA)	0.17	0.01
D-maltose ferulate	C_22_H_30_O_14_	518.47	10	14	8	0.59	225.06	<0./9.69	−3.01	−0.69	No (3; MW, HBD, HBA)	0.17	0.03
D-cellobiose ferulate	C_22_H_30_O_14_	518.47	9	14	8	0.59	225.06	<0./9.69	−3.01	−0.69	No (3; MW, HBD, HBA)	0.17	0.03
Xylobiose ferulate	C_20_H_26_O_12_	458.41	7	12	6	0.55	184.6	<0./9.69	−1.78	−1.24	No (2; HBD, HBA)	0.17	0.33
FOS ferulate 2	C_22_H_30_O_14_	518.47	10	14	8	0.59	225.06	<0./9.69	−2.4	−1.01	No (3; MW, HBD, HBA)	0.17	0.27
Galactobiose ferulate	C_32_H_38_O_17_	694.63	14	17	8	0.44	260.59	<0./9.69	−0.74	−2.94	No (3; MW, HBD, HBA)	0.17	0.03
Arabinobiose ferulate	C_31_H_36_O_14_	632.61	14	14	5	0.42	199.9	<0./9.69	1.22	−3.8	No (2; MW, HBA)	0.17	0.07
Raffinose ferulate	C_29_H_42_O_18_	678.63	13	18	10	0.69	283.98	<0./12.89	−3.58	−1.03	No; (3; MW, HBD, HBA)	0.17	−0.21
FOS ferulate 3	C_58_H_64_O_28_	1209.1	29	28	11	0.38	410.8	<0./9.69	1.47	−6.56	No (3; MW, HBD, HBA)	0.17	0.27
d-mannitol ferulate	C_16_H_22_O_9_	358.34	10	9	6	0.44	156.91	<0./9.69	−1.32	−0.75	Yes (1; HBD)	0.55	−0.26
d-sorbitol ferulate	C_16_H_22_O_9_	358.34	10	9	6	0.44	156.91	<0./9.69	−1.32	−0.75	Yes (1; HBD)	0.55	−0.26
d-xylitol ferulate	C_15_H_20_O_8_	328.31	9	8	5	0.40	136.68	<0./9.69	−0.7	−1.03	Yes (0)	0.55	−0.26
FA rutinoside	C_23_H_32_O_13_	516.49	9	13	7	0.61	204.83	<0./4.07	−1.81	−1.43	No (3; MW, HBD, HBA)	0.11	−0.22
Compound 7a	C_24_H_27_NO_4_	393.48	12	4	1	0.29	56.79	−1.12/15.25	3.96	−4.29	Yes (0)	0.55	0.81
Compound 13b	C_22_H_24_ClNO_4_	401.88	11	4	1	0.32	56.79	−1.89/13.89	3.5	−4.13	Yes (0)	0.55	0.92
Compound 4f	C_29_H_31_NO_5_	473.56	14	5	1	0.28	66.02	−1.89/13.89	4.33	−4.96	Yes (0)	0.55	0.42
Compound 2	C_21_H_23_NO_8_S	449.47	12	8	0	0.29	133.1	<0./17.65	3.63	−4.41	Yes (0)	0.55	−0.54
Compound g18	C_29_H_30_ClF_3_NO_6_P	611.97	15	9	1	0.28	92.9	−5.09/12.64	6.18	−6.86	No (2; MW, logP)	0.17	0.37
Compound e27	C_26_H_22_N_2_O_6_	458.46	9	7	0	0.12	88.88	2.31/25.16	4.43	−5.36	Yes (0)	0.55	0.59
Compound e28	C_29_H_26_N_2_O_6_	498.53	11	7	0	0.14	88.88	2.31/19.29	5.5	−6.11	Yes (0)	0.55	0.54
Compound 5	C_32_H_34_N_2_O_7_	558.62	10	7	2	0.28	98.72	6.30/9.69	5.28	−6.33	Yes (1; MW)	0.55	1.4
Compound 4n	C_39_H_38_BrNO_11_	776.62	17	11	1	0.23	133.15	1.05/13.50	7.16	−8.44	No (2; MW, HBA)	0.17	0.6
Compound 2a	C_26_H_32_O_8_S_2_	536.66	17	8	2	0.38	162.12	<0./15.39	3.44	−4.46	Yes (1; MW)	0.55	0.84
Compound 2y	C_23_H_26_O_7_S_2_	478.58	14	7	2	0.30	152.89	<0./15.39	3.34	−4.26	Yes (0)	0.55	0.45
Compound 2s	C_18_H_16_O_2_S_2_	328.45	5	2	0	0.17	76.9	<0./25.90	4.76	−4.95	Yes (1; logP)	0.55	0.05
Compound F3	C_28_H_25_FO_6_	476.49	12	7	0	0.14	71.06	<0./27.89	5.68	−5.96	Yes (0)	0.55	0.02
Compound D4	C_22_H_20_N_2_O_3_S	392.47	9	4	1	0.09	88.16	1.11/13.72	4.71	−5.1	Yes (0)	0.55	−0.42
DiFA	C_20_H_18_O_8_	386.35	7	8	4	0.10	133.52	<0./4.54	2.69	−3.79	Yes (0)	0.56	0
TriFA	C_29_H_26_O_10_	534.51	10	10	3	0.17	140.98	<0./4.54	4.16	−5.46	Yes (1; MW)	0.56	0.32
Compound MY3	C_16_H_21_NO_4_	291.34	7	4	0	0.44	72.12	<0./20.14	4.32	−4.12	Yes (0)	0.55	−0.61

^1^: Molecular weight; ^2^: Number of rotatable bonds; ^3^: Number of hydrogen bond acceptors; ^4^: Number of hydrogen bond donors; ^5^: The ratio of sp^3^ hybridized carbons over the total carbon count of the molecule; ^6^: Topological polar surface area, as calculated by SwissADME; ^7^: As calculated by MOLSOFT; ^8^: The octanol water partition coefficient, as calculated by the program XLOGP through SwissADME; ^9^: LogS calculated by SwissADME, as a measure of solubility. Based on its value, the compounds are categorized into insoluble (logS < −10), poorly soluble (−10 < logS < −6), moderately soluble (−6 < logS < −4), soluble (−4 < logS < −2), very soluble (−2 < logS < 0), and highly soluble (logS > 0); ^10^: Lipinski´s rule of 5 sets 5 criteria, the violation 2 or more of which indicates low oral bioavailability of a compound. In the parentheses, the number and description of the criteria violated are given; ^11^: Probability of a compound to have a bioavailability of more than 10% in rats, given by SwissADME; ^12^: Druglikeness as calculated by MOLSOFT. Overall, positive values prompt a drug-like compound.

**Table 6:** Pharmacokinetic and toxicity predictions for FA and its selected derivatives.

Compound Name	GI Absorption ^1^	BBB ^2^ Permeant	P-gp Substrate ^3^	CYP1A2 Inhibitor	CYP2C19 Inhibitor	CYP2C9 Inhibitor	CYP2D6 Inhibitor	CYP3A4 Inhibitor	Log Kp ^4^ (cm/s)	LD_50_ ^5^ (mg/kg)	Hepatotoxicity		Carcinogenicity		Mutagenicity		Cytotoxicity	
											A ^6^	P ^7^	A	P	A	P	A	P
FA	High	Yes	No	No	No	No	No	No	−6.41	1772		0.51		0.61		0.96		0.88
Methyl ferulate	High	Yes	No	No	No	No	No	No	−6.26	978		0.56		0.67		0.89		0.94
Ethyl ferulate	High	Yes	No	Yes	No	No	No	No	−6.09	978		0.67		0.73		0.82		0.91
Propyl ferulate	High	Yes	No	Yes	Yes	No	No	No	−5.8	978		0.6		0.77		0.81		0.87
Butyl ferulate	High	Yes	No	Yes	Yes	No	No	No	−5.63	9600		0.6		0.78		0.8		0.86
Isobutyl ferulate	High	Yes	No	Yes	Yes	No	No	No	−5.58	978		0.6		0.7		0.81		0.84
Pentyl ferulate	High	Yes	No	Yes	Yes	No	No	No	−5.34	9600		0.72		0.74		0.77		0.85
Isopentyl ferulate	High	Yes	No	Yes	Yes	No	No	No	−5.41	9600		0.53		0.69		0.79		0.81
Prenyl ferulate	High	Yes	No	Yes	Yes	No	No	No	−5.53	978		0.57		0.68		0.7		0.75
Hexyl ferulate	High	Yes	No	Yes	Yes	No	No	No	−5.04	9600		0.8		0.76		0.72		0.82
Octyl ferulate	High	Yes	No	Yes	No	Yes	Yes	No	−4.44	9600		0.8		0.76		0.72		0.82
Dodecyl ferulate	High	No	No	Yes	No	Yes	Yes	No	−3.24	9600		0.8		0.76		0.72		0.82
Octadecyl ferulate	Low	No	No	No	Yes	No	No	No	−1.45	9600		0.8		0.76		0.72		0.82
Oleyl ferulate	Low	No	No	No	No	No	No	Yes	−2.1	9600		0.8		0.76		0.72		0.82
Glyceryl ferulate	High	No	No	No	No	No	No	No	−7.56	978		0.89		0.83		0.76		0.86
Diglyceryl ferulate	High	No	No	No	No	No	No	No	−8.55	978		0.9		0.83		0.77		0.83
Tocopheryl ferulate	Low	No	Yes	No	No	No	No	No	−1.12	5000		0.74		0.68		0.73		0.81
Sitosteryl ferulate	Low	No	No	No	No	No	No	No	−1.66	9600		0.74		0.66		0.93		0.79
D-glucose ferulate	Low	No	No	No	No	No	No	No	−9.09	5000		0.78		0.8		0.77		0.83
D-galactose ferulate	Low	No	No	No	No	No	No	No	−9.09	5000		0.78		0.8		0.77		0.83
D-mannose ferulate	Low	No	No	No	No	No	No	No	−9.09	5000		0.78		0.8		0.77		0.83
D-fructose ferulate	Low	No	No	No	No	No	No	No	−8.87	5000		0.89		0.84		0.74		0.82
L-arabinose ferulate	High	No	No	No	No	No	No	No	−8.47	5000		0.76		0.76		0.69		0.85
D-xylose ferulate	High	No	No	No	No	No	No	No	−8.47	5000		0.76		0.76		0.69		0.85
FOS ferulate 1	Low	No	Yes	No	No	No	No	No	−8.33	5000		0.87		0.84		0.77		0.76
D-lactose ferulate	Low	No	No	No	No	No	No	No	−11.51	5000		0.75		0.81		0.69		0.72
D-sucrose ferulate	Low	No	No	No	No	No	No	No	−10.87	5000		0.87		0.83		0.77		0.79
D-maltose ferulate	Low	No	No	No	No	No	No	No	−11.6	5000		0.81		0.81		0.79		0.78
D-cellobiose ferulate	Low	No	No	No	No	No	No	No	−11.6	5000		0.81		0.81		0.79		0.78
Xylobiose ferulate	Low	No	No	No	No	No	No	No	−10.36	5000		0.77		0.76		0.8		0.75
FOS ferulate 2	Low	No	No	No	No	No	No	No	−11.17	n.a.		0.91		0.84		0.77		0.77
Galactobiose ferulate	Low	No	Yes	No	No	No	No	No	−11.06	5000		0.83		0.8		0.81		0.79
Arabinobiose ferulate	Low	No	Yes	No	No	No	No	No	−9.29	5000		0.77		0.74		0.78		0.76
Raffinose ferulate	Low	No	No	No	No	No	No	No	−12.98	5000		0.87		0.86		0.79		0.77
FOS ferulate 3	Low	No	Yes	No	No	No	No	No	−12.63	n.a.		0.88		0.83		0.76		0.73
D-mannitol ferulate	Low	No	No	No	No	No	No	No	−9.42	9600		0.87		0.86		0.77		0.84
D-sorbitol ferulate	Low	No	No	No	No	No	No	No	−9.42	9600		0.87		0.86		0.77		0.84
D-xylitol ferulate	Low	No	No	No	No	No	No	No	−8.8	9600		0.9		0.87		0.77		0.84
FA rutinoside	Low	No	No	No	No	No	No	No	−10.74	4000		0.82		0.86		0.82		0.73
Compound 7a	High	Yes	Yes	Yes	No	Yes	Yes	Yes	−5.89	1500		0.76		0.53		0.69		0.78
Compound 13b	High	Yes	Yes	Yes	Yes	Yes	Yes	Yes	−6.27	1200		0.83		0.59		0.73		0.7
Compound 4f	High	Yes	Yes	No	Yes	Yes	Yes	Yes	−6.11	5300		0.88		0.56		0.72		0.74
Compound 2	Low	No	Yes	No	Yes	Yes	Yes	Yes	−6.46	1000		0.51		0.66		0.69		0.72
Compound g18	Low	No	Yes	No	Yes	Yes	Yes	Yes	−5.65	1500		0.67		0.65		0.66		0.72
Compound e27	High	No	No	No	Yes	Yes	No	Yes	−5.95	1000		0.76		0.52		0.53		0.63
Compound e28	High	No	No	No	Yes	Yes	No	Yes	−5.44	1000		0.75		0.51		0.55		0.62
Compound 5	High	No	Yes	No	Yes	Yes	No	No	−5.96	600		0.86		0.57		0.58		0.57
Compound 4n	Low	No	No	No	No	No	No	No	−5.95	5000		0.51		0.52		0.6		0.59
Compound 2a	Low	No	No	No	Yes	No	Yes	No	−7.13	1772		0.84		0.68		0.81		0.79
Compound 2y	Low	No	No	No	Yes	Yes	Yes	No	−6.85	1000		0.76		0.75		0.84		0.79
Compound 2s	High	No	No	Yes	Yes	Yes	No	No	−4.92	3150		0.54		0.55		0.74		0.74
Compound F3	High	No	Yes	No	No	Yes	No	Yes	−5.17	2100		0.58		0.68		0.82		0.62
Compound D4	High	No	No	Yes	Yes	Yes	No	Yes	−5.35	3506		0.61		0.59		0.52		0.72
DiFA	High	No	No	No	No	No	No	No	−6.75	1100		0.52		0.67		0.83		0.63
TriFA	Low	No	No	No	No	Yes	No	No	−6.61	1772		0.65		0.51		0.58		0.79
Compound MY3	High	Yes	No	Yes	Yes	No	No	No	−5.01	5000		0.64		0.6		0.61		0.71

^1^: Gastrointestinal absorption; ^2^: Blood brain barrier; ^3^: P-glycoprotein; ^4^: Measure of skin permeation. The more negative the value of logK_p_, the lower the skin permeability indicated. All of the aforementioned pharmacokinetic properties were predicted by SwissADME; ^5^: Lethal dose, as calculated by ProToxII. Based on this value, chemicals are categorized in 6 toxicity classes. Compounds in this study fall under the three less toxic categories: Class IV: harmful if swallowed (300 < LD50 ≤ 2000); Class V: may be harmful if swallowed (2000 < LD50 ≤ 5000); and Class VI: non-toxic (LD50 > 5000); ^6^: Prediction for the inactivity (green) or activity (red) of the compound in the respective toxicity category, as given by ProToxII; ^7^: Probability of this prediction, given by ProToxII. An encouraging indicator is that most of the compounds are not P-glycoprotein or cytochrome inhibitors, meaning that they can be selective M^pro^ targets, while they also seem to have acceptable skin permeability, taking into consideration that more negative values of logK_p_ are interpreted as lower skin permeability [115]. As far as blood brain barrier (BBB) permeability is concerned, most compounds are not estimated to be able to permeate the BBB. This means that unwanted side-effects in the central nervous system are avoided. Nevertheless, there have been indications that SARS-CoV-2 does cross the barrier, potentially causing brain damage [118], so it is not entirely clear whether BBB permeability is an undesired property.

## Data Availability

Data is contained within the article and Supplemental Materials.

## Supplementary Materials

The following supporting information can be downloaded at: https://www.mdpi.com/article/10.3390/biomedicines10081787/s1, Figure S1: Binding modes of the second and third clusters for alkyl and alkenyl FA esters at the active site of M^pro^; Figure S2: Binding modes of the second and third clusters for fatty acid, polyol, tocopherol, and sterol FA derivatives at the active site of M^pro^; Figure S3: Binding modes of the second and third clusters for monosaccharide esters of FA at the active site of M^pro^; Figure S4: Binding modes of the second and third clusters for disaccharide esters of FA at the active site of M^pro^; Figure S5: Binding modes of the second and third clusters for trisaccharide and sugar alcohol esters of FA at the active site of M^pro^; Figure S6: Binding modes of the second and third clusters for FOS esters of FA at the active site of M^pro^; Figure S7: Binding modes of the second and third clusters for FA rutinoside at the active site of M^pro^; Figure S8: Binding modes of the second and third clusters for chemically synthesized FA derivatives at the active site of M^pro^.
