# Supplementary material for: The Inhibitory Potential of Ferulic Acid Derivatives against the SARS-CoV-2 Main Protease: Molecular Docking, Molecular Dynamics, and ADMET Evaluation
Source: Biomedicines. 2022 Jul 25;10(8):1787. doi: 10.3390/biomedicines10081787 (PMC9329733; doi:10.3390/biomedicines10081787)
Supplement: Supplementary file 1 [file biomedicines-10-01787-s001.zip › biomedicines-1796204-supplementary.pdf]

Supplementary material

The inhibitory potential of ferulic acid derivatives against the main protease (M^{pro}) of SARS-CoV-2: molecular docking, molecular dynamics and ADMET evaluation

Io Antonopoulou[1]*, Eleftheria Sapountzaki[1], Ulrika Rova, Paul Christakopoulos

Biochemical Process Engineering, Division of Chemical Engineering, Department of Civil, Environmental and Natural Resources Engineering, Luleå University of Technology, SE-97187 Luleå, Sweden

*Corresponding author: e-mail: io.antonopoulou@ltu.se

[1]: equal contribution

Methyl ferulate|Cluster 2|5.158 kcal/mol 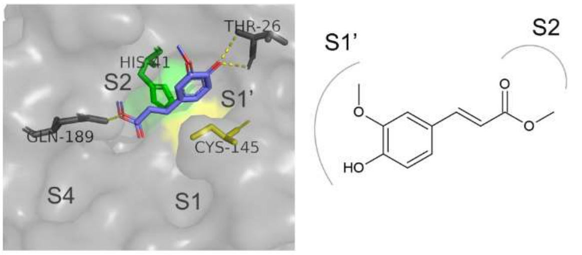	Methyl ferulate|Cluster 3|5.147 kcal/mol 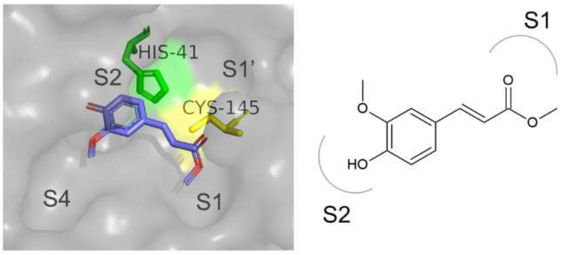
Ethyl ferulate|Cluster 2|5.525 kcal/mol 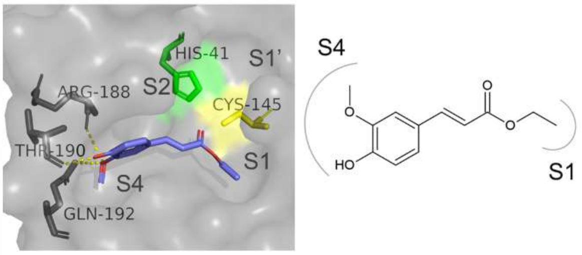	Ethyl ferulate|Cluster 3|5.477 kcal/mol 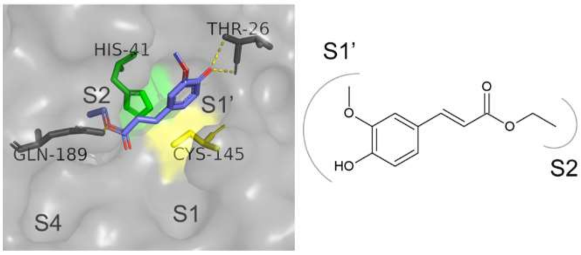
Propyl ferulate|Cluster 2|5.481 kcal/mol 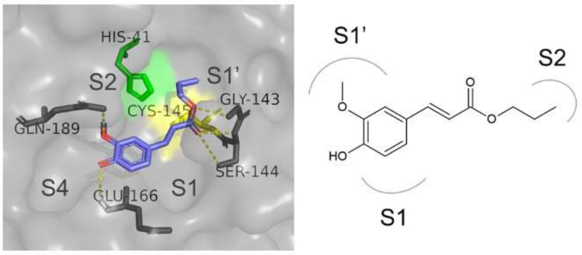	Propyl ferulate|Cluster 3|5.324 kcal/mol 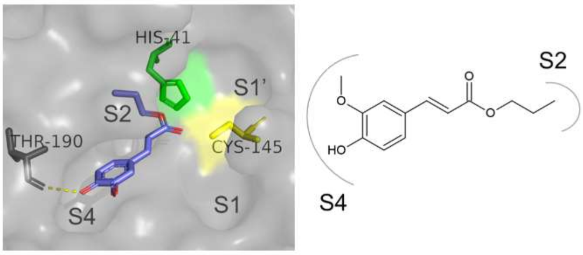
Butyl ferulate|Cluster 2|5.568 kcal/mol 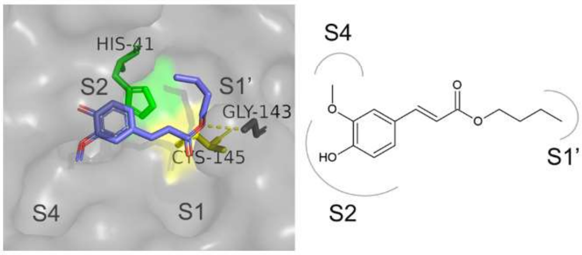	Butyl ferulate|Cluster 3|5.553 kcal/mol 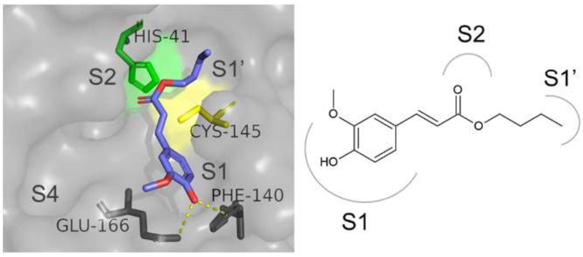
Isobutyl ferulate|Cluster 2|6.039 kcal/mol 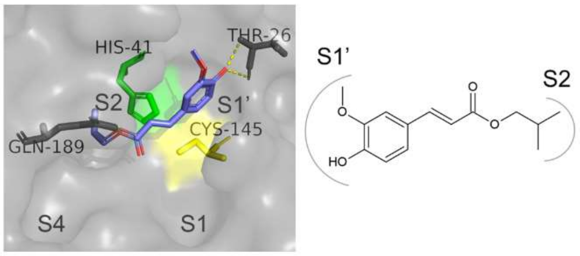	Isobutyl ferulate|Cluster 3|5.896 kcal/mol 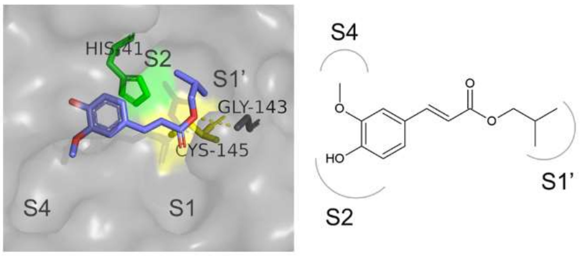

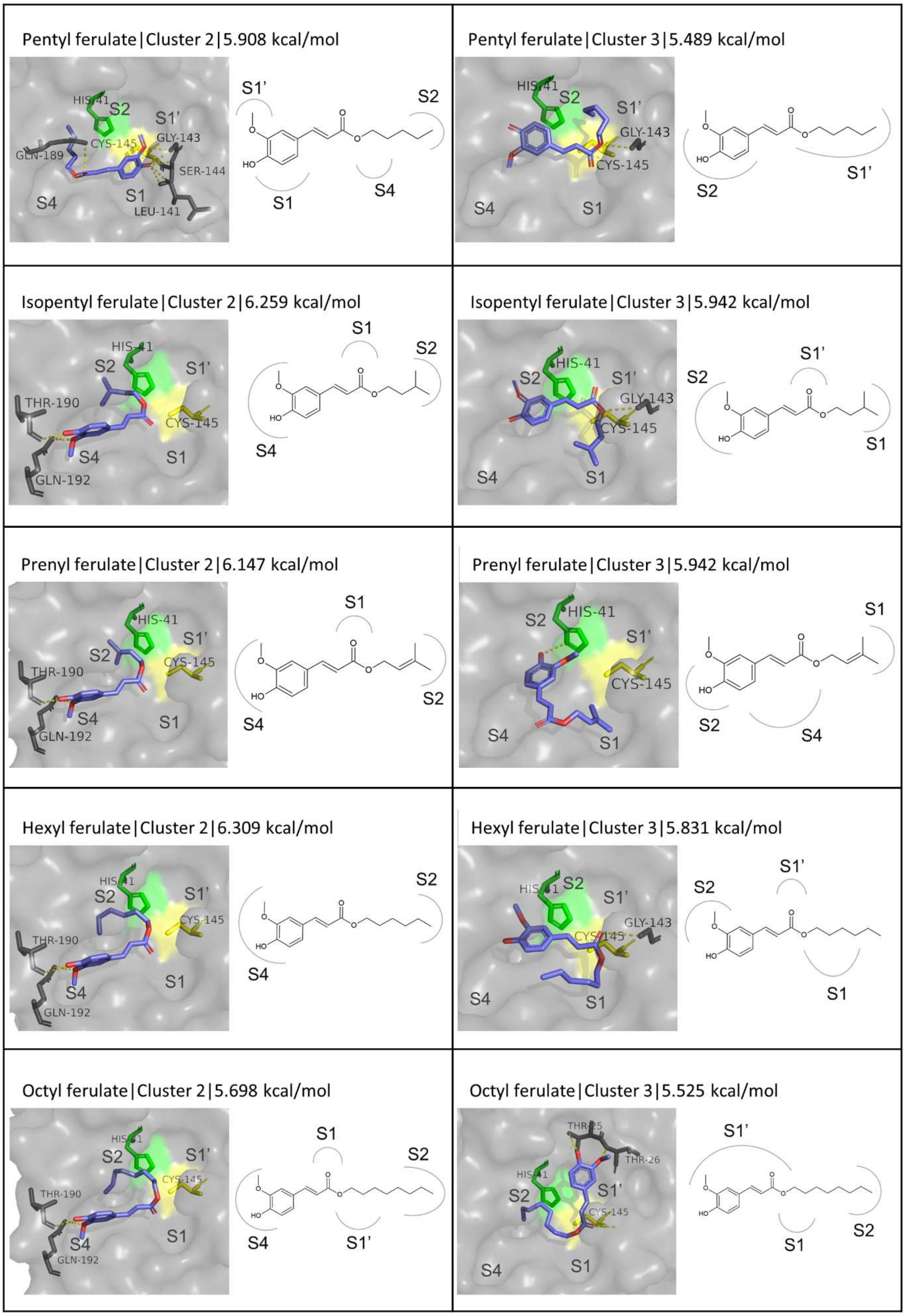
Pentyl ferulate|Cluster 2|5.908 kcal/mol
HIS-41
S2
S1'
CYS-145
GLY-143
GLN-189
SER-144
S1
LEU-141
S4
S1'
S2
HO
S1
S4
Pentyl ferulate|Cluster 3|5.489 kcal/mol
HIS-41
S2
S1'
GLY-143
CYS-145
S4
S1
HO
S2
S1'
Isopentyl ferulate|Cluster 2|6.259 kcal/mol
HIS-41
S2
S1'
THR-190
CYS-145
S4
S1
GLN-192
S1
S2
HO
S4
Isopentyl ferulate|Cluster 3|5.942 kcal/mol
HIS-41
S2
S1'
GLY-143
CYS-145
S4
S1
S2
S1'
HO
S1
Prenyl ferulate|Cluster 2|6.147 kcal/mol
HIS-41
S1'
S2
CYS-145
THR-190
S4
S1
GLN-192
S1
HO
S2
S4
Prenyl ferulate|Cluster 3|5.942 kcal/mol
S2
HIS-41
S1'
CYS-145
S4
S1
S1
HO
S2
S4
Hexyl ferulate|Cluster 2|6.309 kcal/mol
HIS-41
S2
S1'
CYS-145
THR-190
S4
S1
GLN-192
S2
HO
S4
Hexyl ferulate|Cluster 3|5.831 kcal/mol
HIS-41
S2
S1'
GLY-143
CYS-145
S4
S1
S2
S1'
HO
S1
Octyl ferulate|Cluster 2|5.698 kcal/mol
HIS-41
S2
S1'
THR-190
CYS-145
S4
S1
GLN-192
S1
S2
HO
S4
S1'
Octyl ferulate|Cluster 3|5.525 kcal/mol
THR-25
THR-26
HIS-41
S2
S1'
CYS-145
S4
S1
S1'
HO
S1
S2

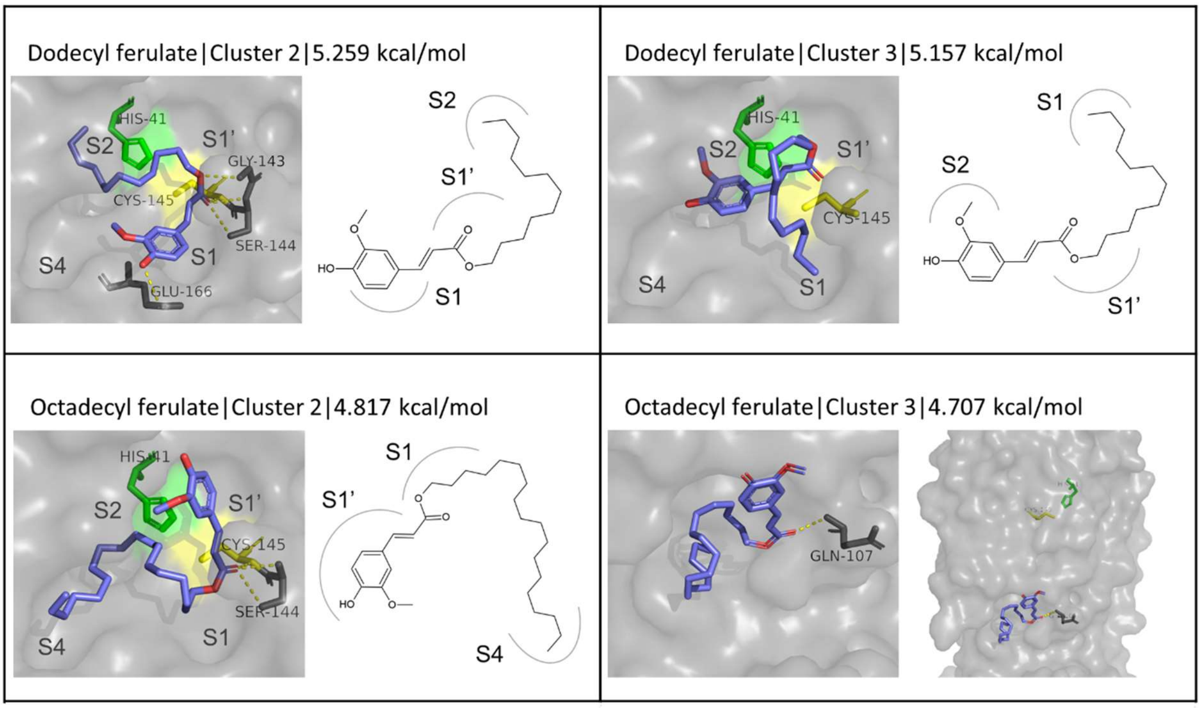

Figure S1. Binding modes of the second and third clusters for alkyl and alkenyl FA esters in the active site of M^{pro}.

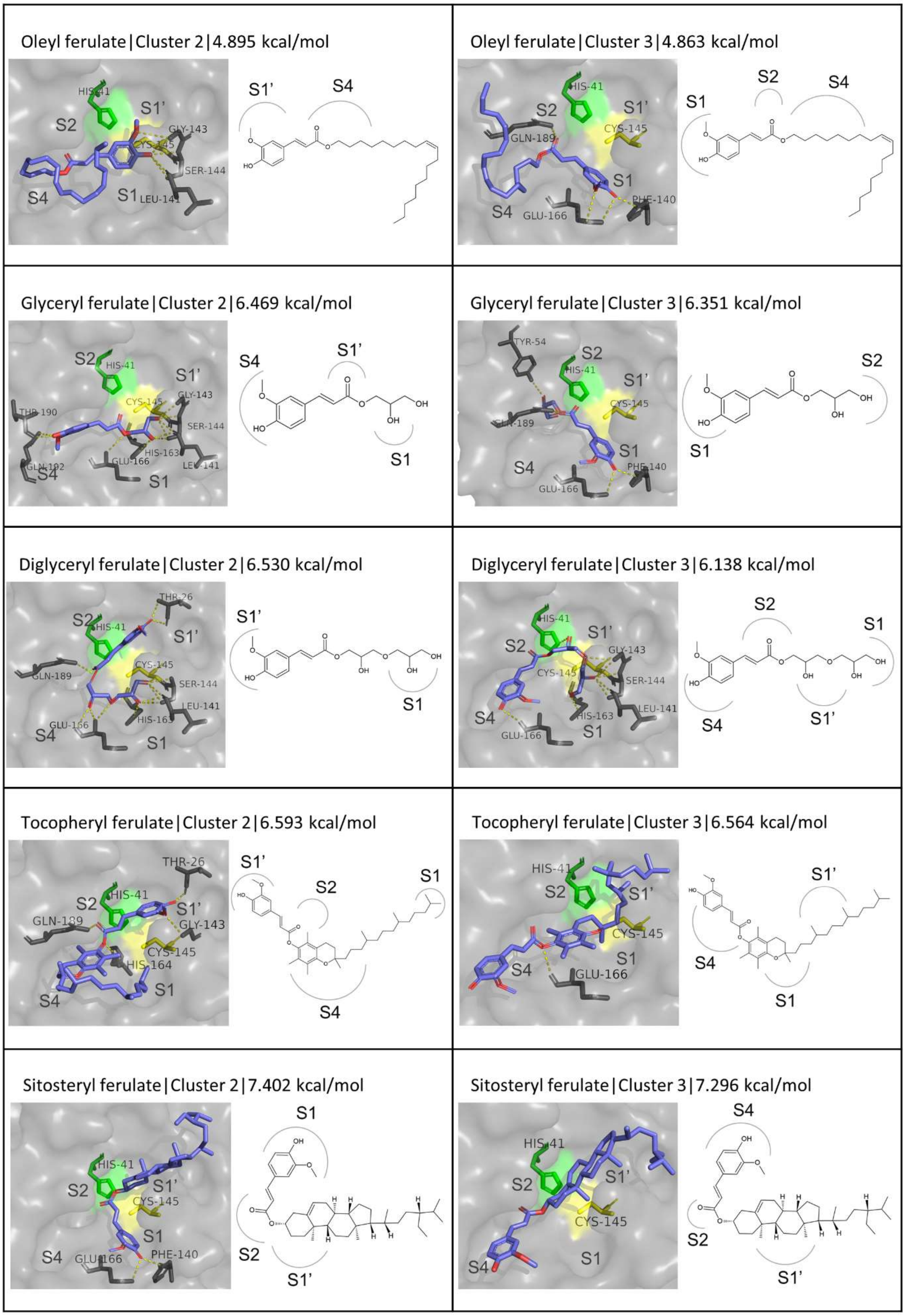

Figure S2. Binding modes of the second and third clusters for fatty acid, polyol, tocopherol and sterol FA derivatives in the active site of M^{pro}.

D-glucose ferulate|Cluster 2|7.078 kcal/mol

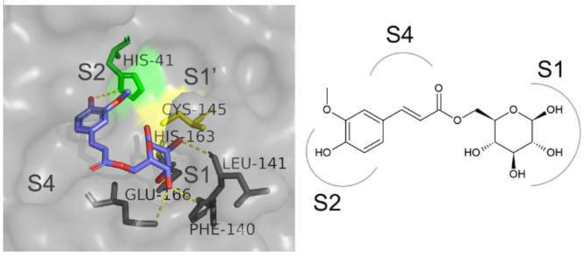

D-glucose ferulate|Cluster 3|6.925 kcal/mol

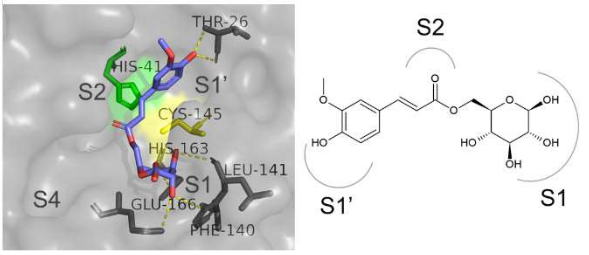

D-galactose ferulate|Cluster 2|6.940 kcal/mol

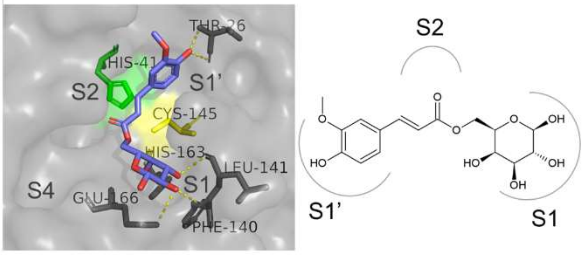

D-galactose ferulate|Cluster 3|6.774 kcal/mol

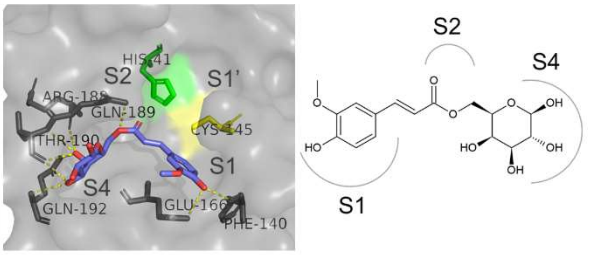

D-mannose ferulate|Cluster 2|7.078 kcal/mol

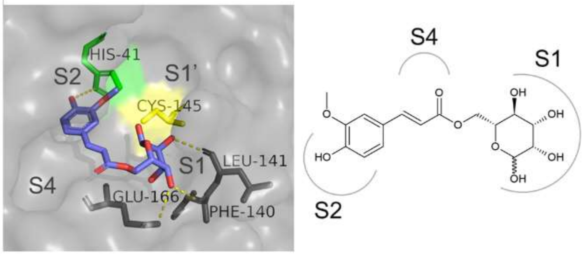

D-mannose ferulate|Cluster 3|6.925 kcal/mol

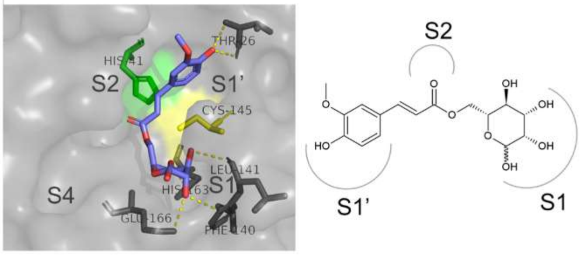

D-fructose ferulate|Cluster 2|6.873 kcal/mol

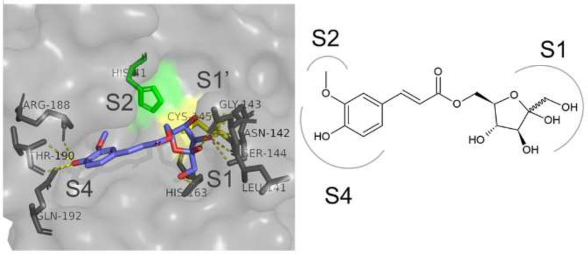

D-fructose ferulate|Cluster 3|6.565 kcal/mol

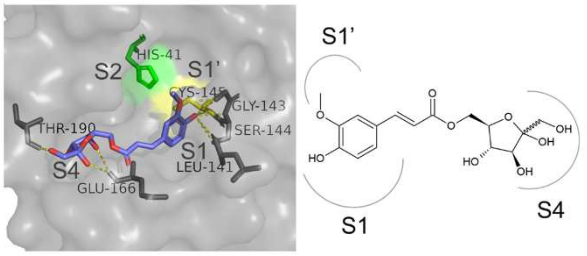

L-arabinose ferulate|Cluster 2|6.975 kcal/mol

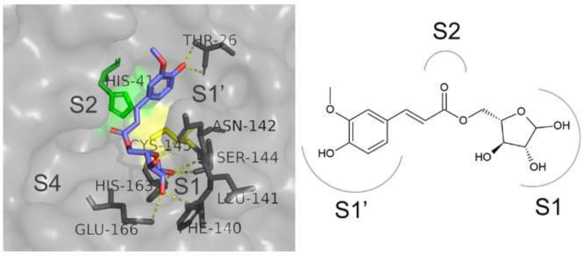

L-arabinose ferulate|Cluster 3|6.879 kcal/mol

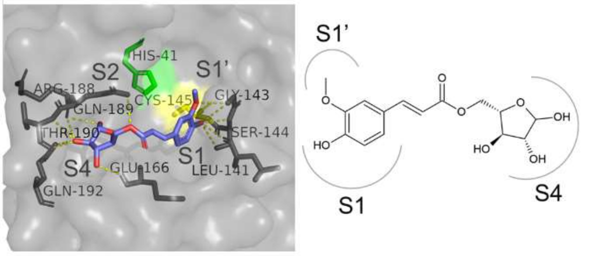

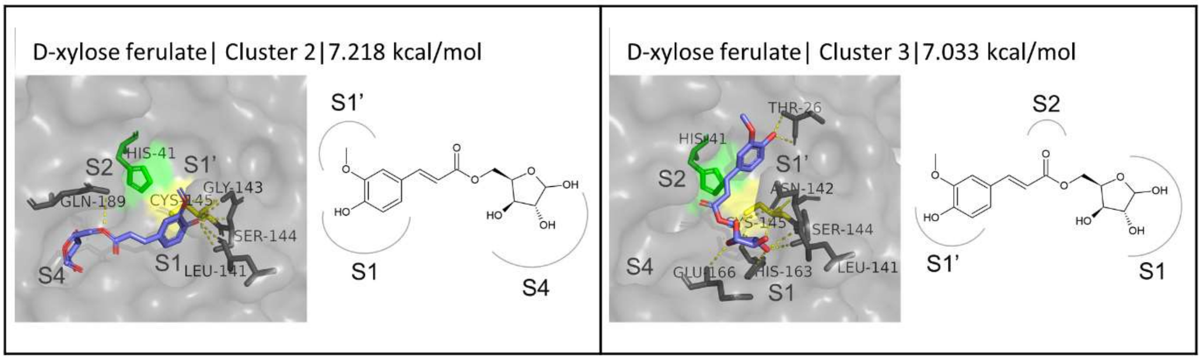

Figure S3. Binding modes of the second and third clusters for monosaccharide esters of FA in the active site of M^{pro}.

D-lactose ferulate|Cluster 2|7.387 kcal/mol

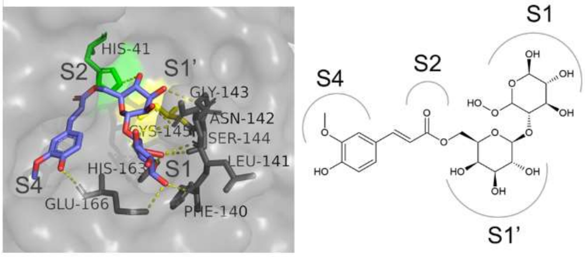

D-lactose ferulate|Cluster 3|7.368 kcal/mol

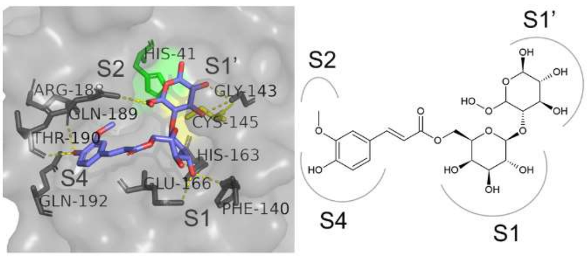

D-sucrose ferulate|Cluster 2|7.625 kcal/mol

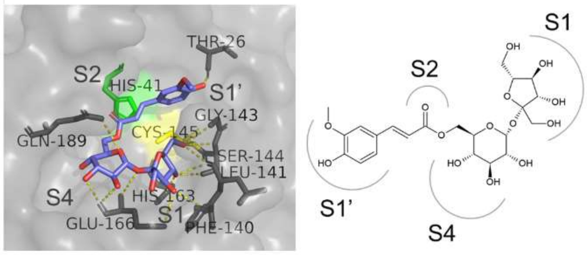

D-sucrose ferulate|Cluster 3|7.319 kcal/mol

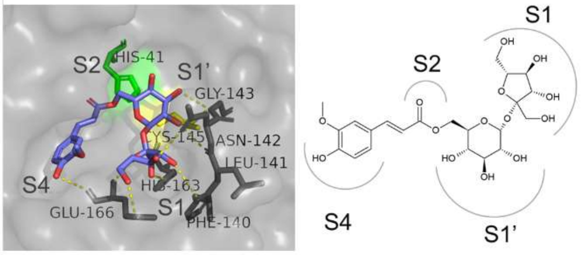

D-maltose ferulate|Cluster 2|7.028 kcal/mol

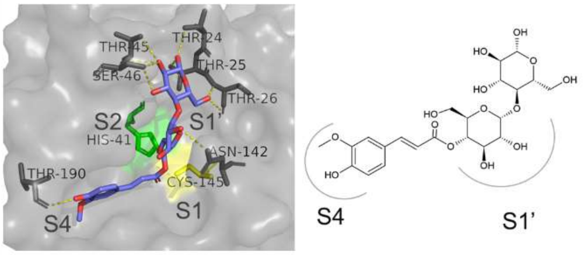

D-maltose ferulate|Cluster 3|6.917 kcal/mol

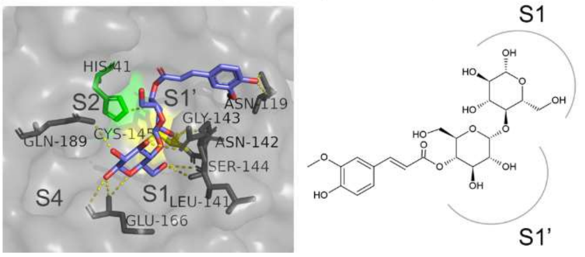

D-cellobiose ferulate|Cluster 2|7.451 kcal/mol

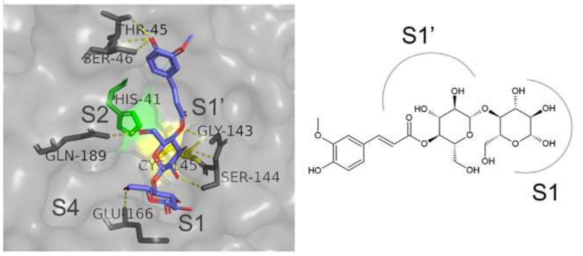

D-cellobiose ferulate|Cluster 3|7.370 kcal/mol

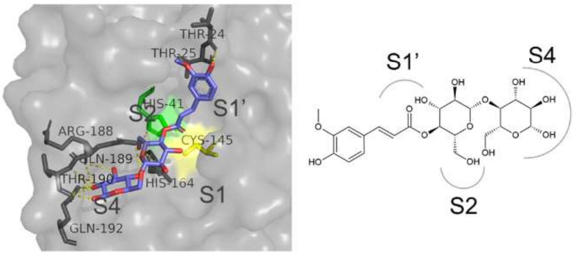

Xylobiose ferulate|Cluster 2|7.359 kcal/mol

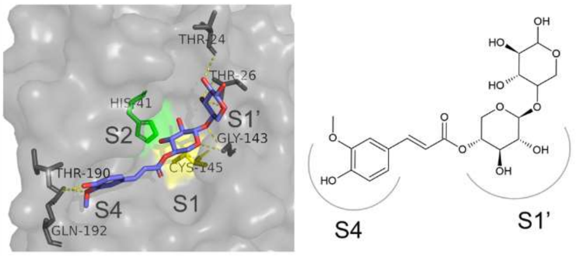

Xylobiose ferulate|Cluster 3|7.328 kcal/mol

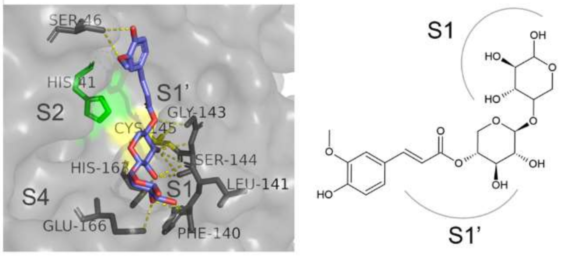

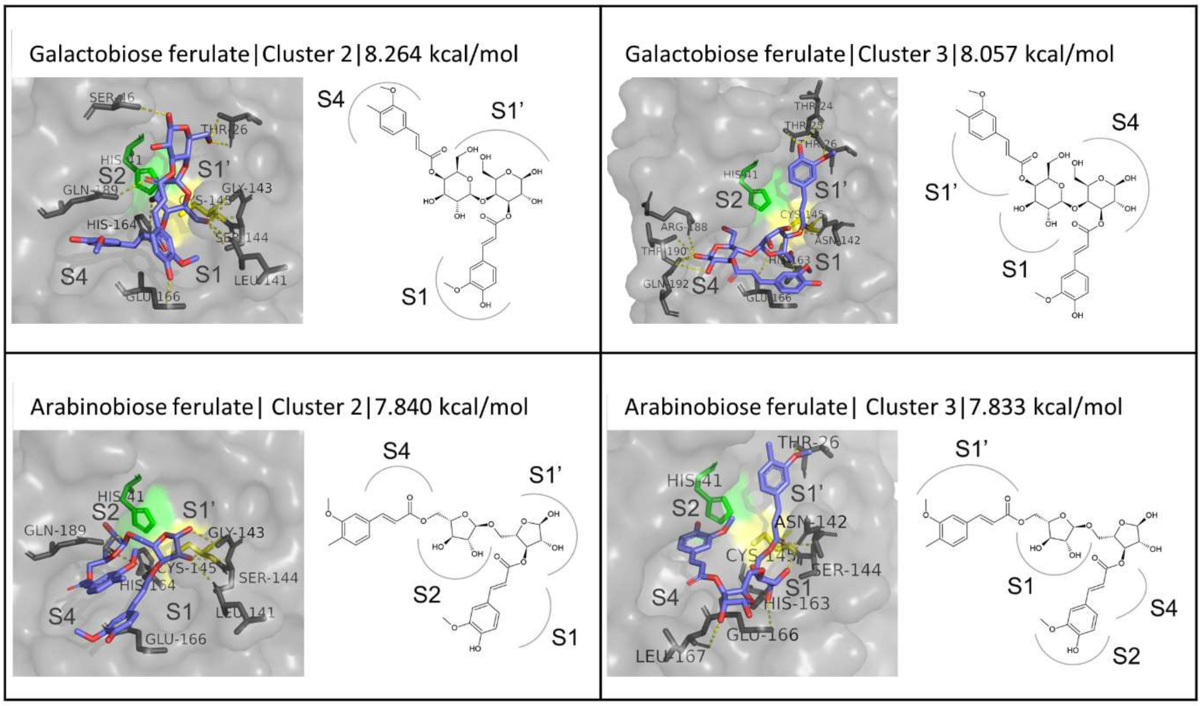

Figure S4. Binding modes of the second and third clusters for disaccharide esters of FA in the active site of M[pro].

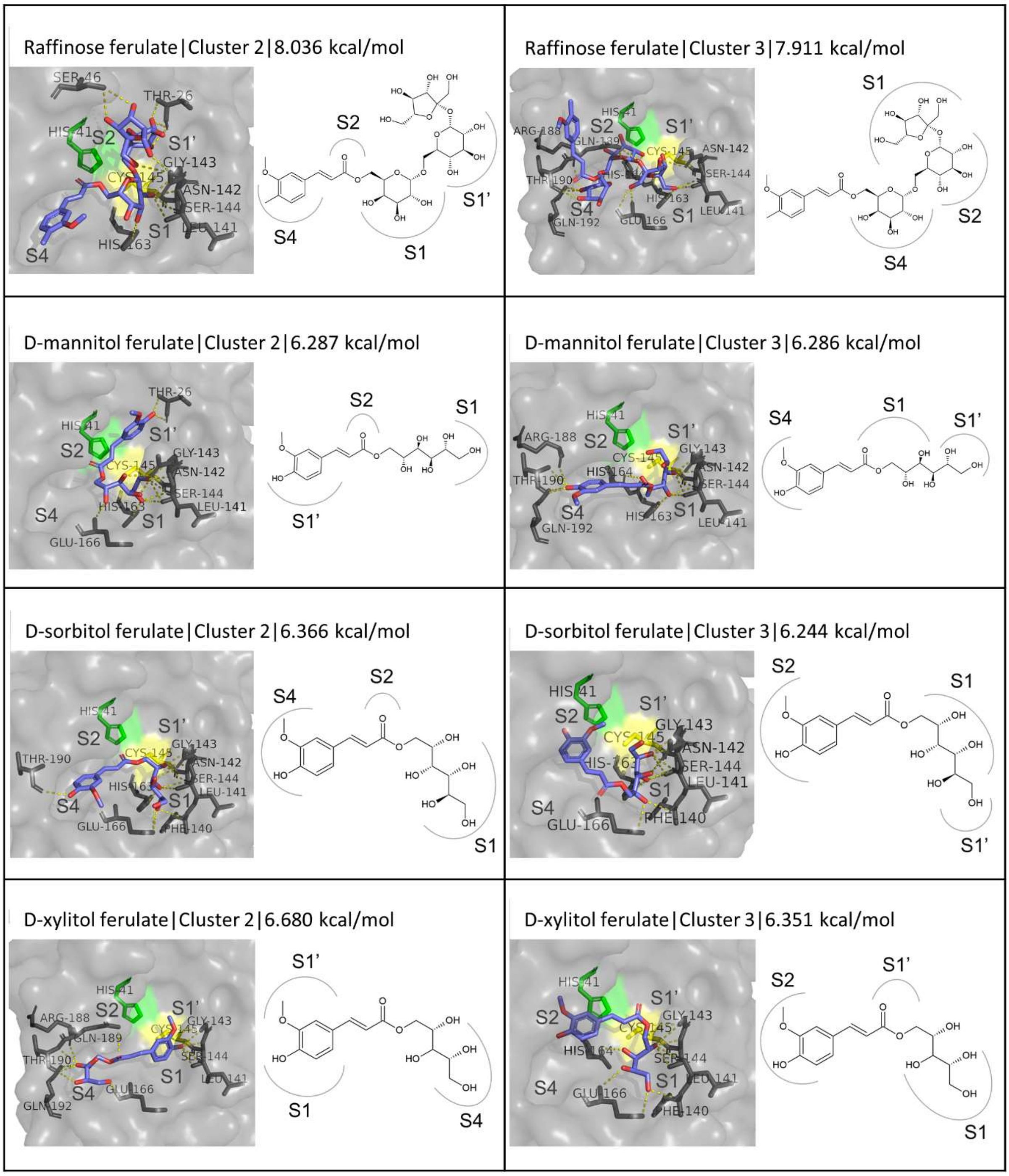

Figure S5. Binding modes of the second and third clusters for trisaccharide and sugar alcohol esters of FA in the active site of M^{pro}.

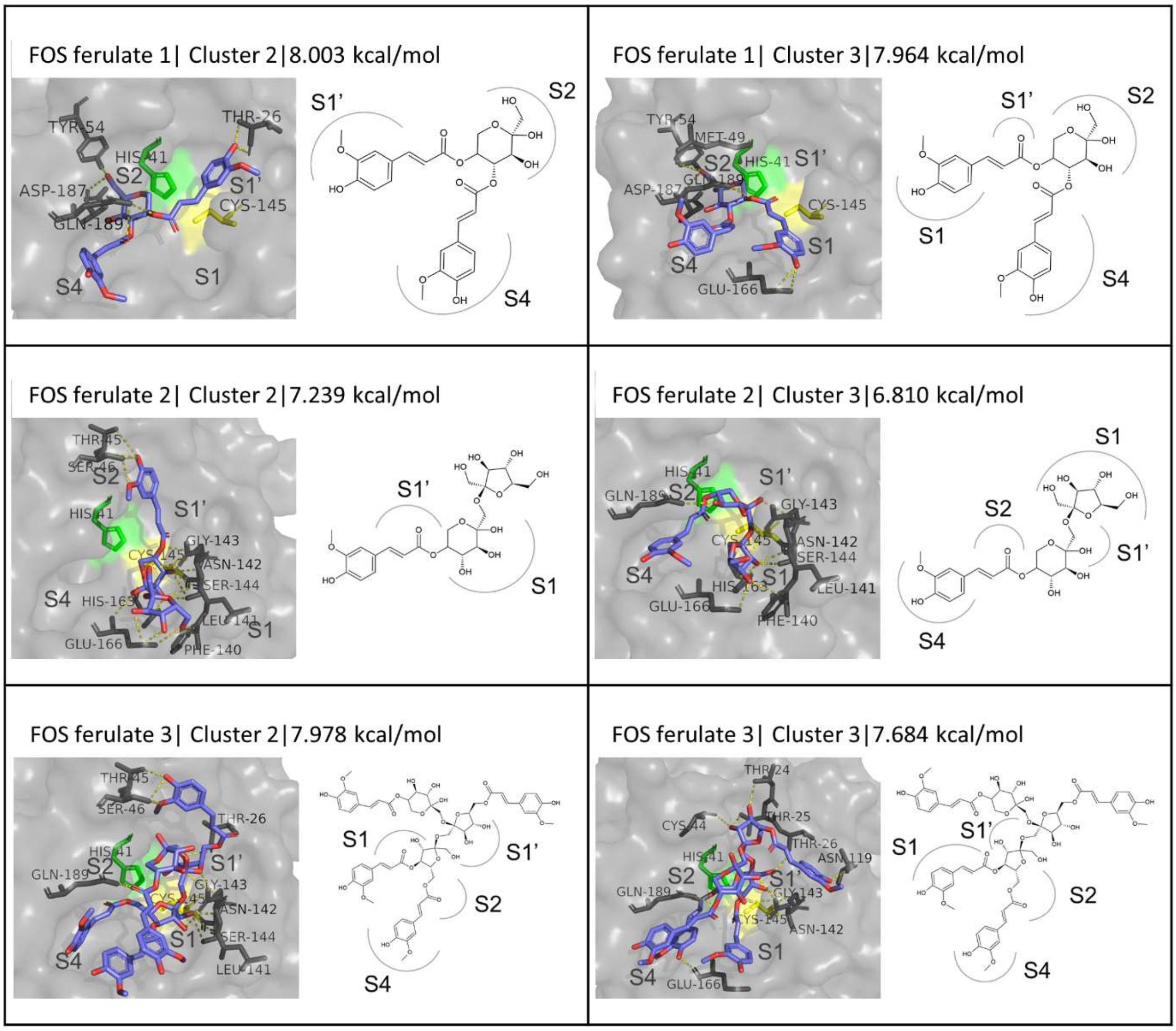

Figure S6. Binding modes of the second and third clusters for FOS esters of FA in the active site of M^{pro}.

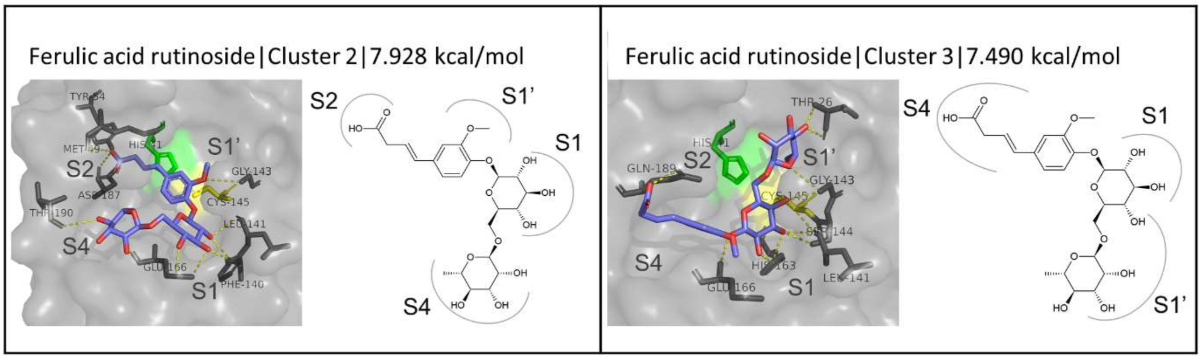

Figure S7. Binding modes of the second and third clusters for FA rutinoside in the active site of M^{pro}.

Compound 7a|Cluster 2|6.557 kcal/mol

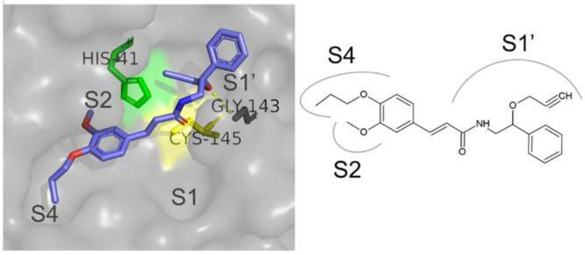

Compound 7a|Cluster 3|6.363 kcal/mol

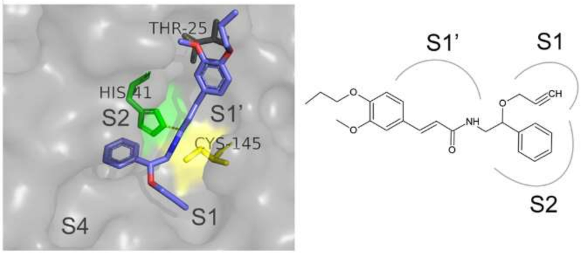

Compound 13b|Cluster 2|6.348 kcal/mol

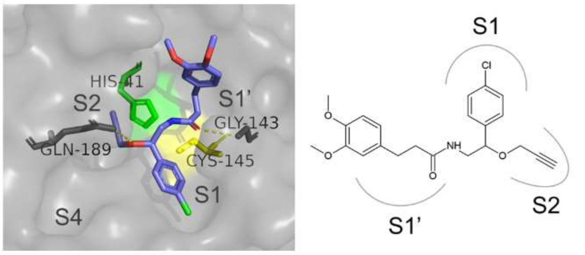

Compound 13b|Cluster 3|6.212 kcal/mol

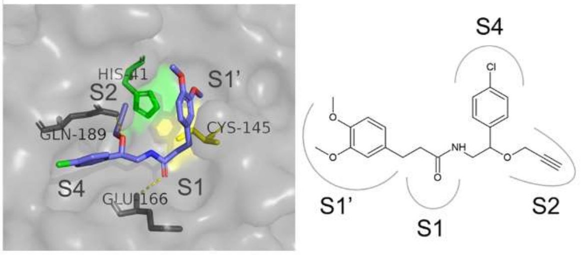

Compound 4f|Cluster 2|6.075 kcal/mol

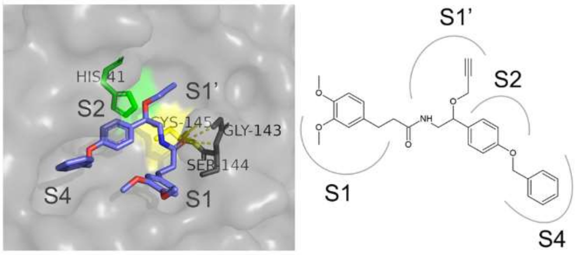

Compound 4f|Cluster 3|5.971 kcal/mol

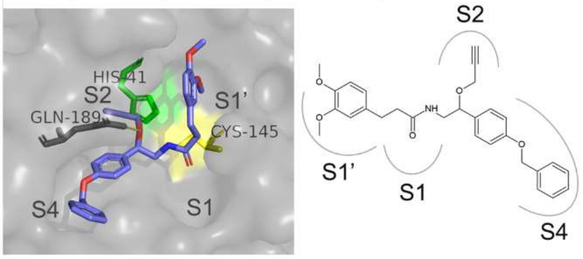

Compound 2|Cluster 2|7.280 kcal/mol

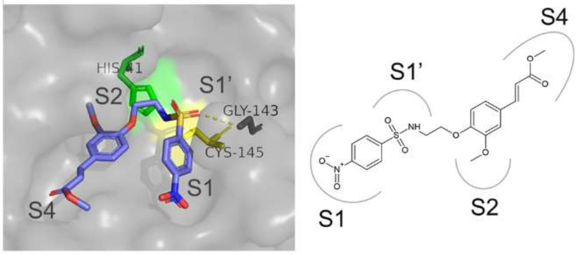

Compound 2|Cluster 3|7.266 kcal/mol

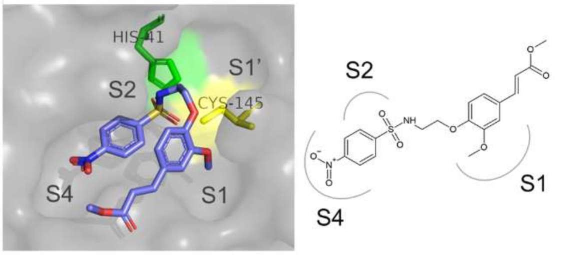

Compound g18|Cluster 2|6.815 kcal/mol

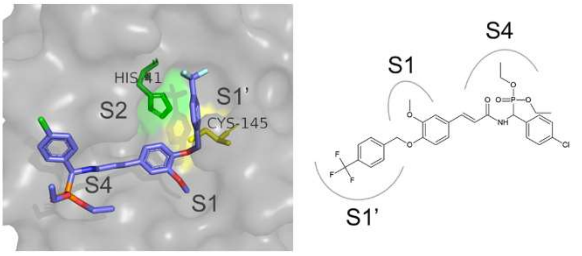

Compound g18|Cluster 3|6.667 kcal/mol

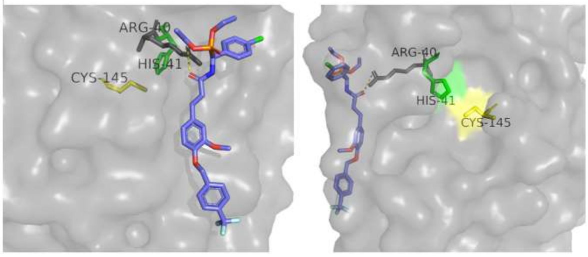

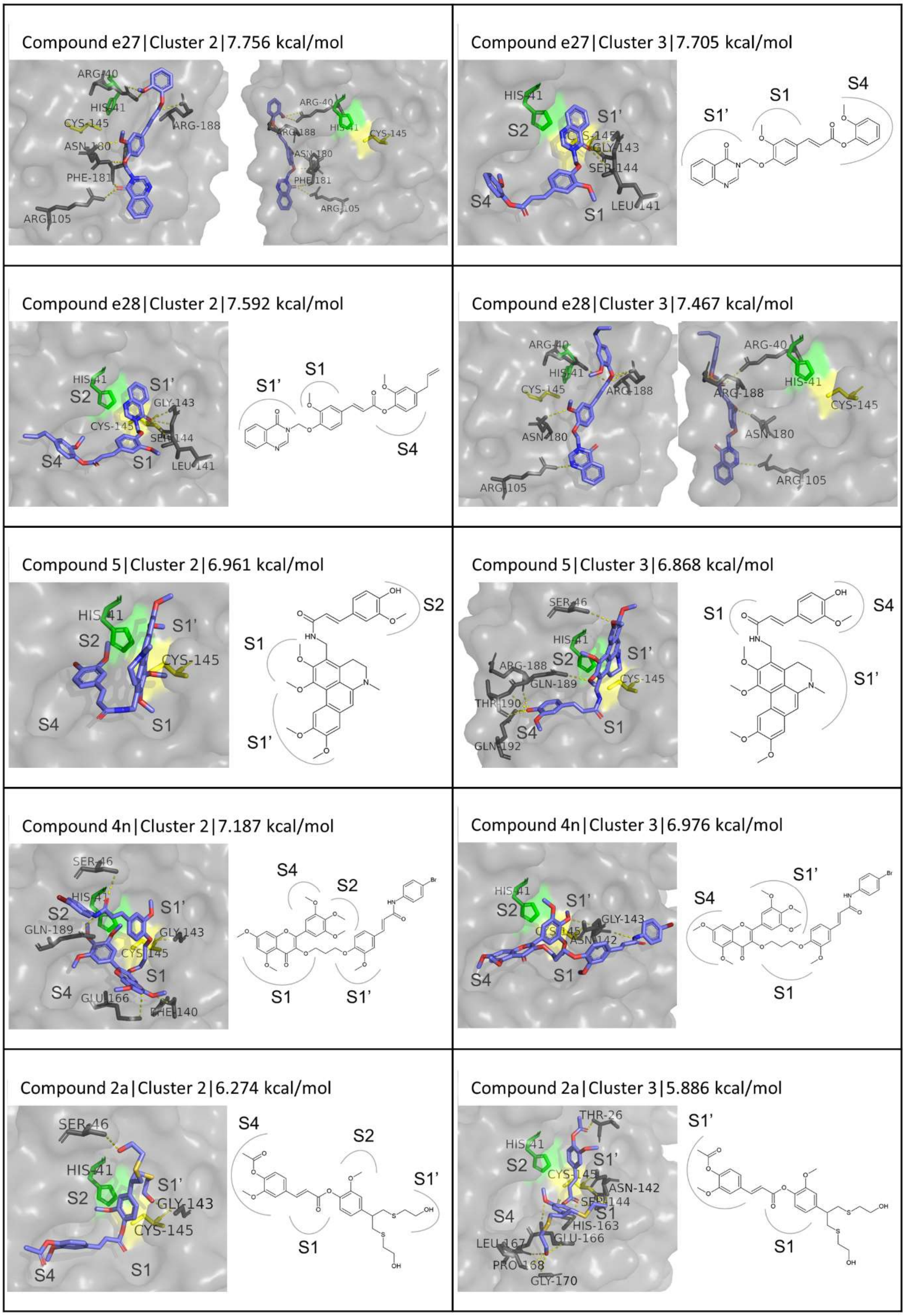
Compound e27|Cluster 2|7.756 kcal/mol
ARG-40
HIS-41
CYS-145
ARG-188
ASN-180
PHE-181
ARG-105
Compound e27|Cluster 3|7.705 kcal/mol
HIS-41
S2
S1'
CYS-145
GLY-143
SER-144
S4
S1
LEU-141
S1'
S1
S4
Compound e28|Cluster 2|7.592 kcal/mol
HIS-41
S2
S1'
GLY-143
CYS-145
SER-144
S4
S1
LEU-141
S1'
S1
S4
Compound e28|Cluster 3|7.467 kcal/mol
ARG-40
HIS-41
CYS-145
ARG-188
ASN-180
ARG-105
Compound 5|Cluster 2|6.961 kcal/mol
HIS-41
S1'
S2
CYS-145
S4
S1
S2
S1
S1'
Compound 5|Cluster 3|6.868 kcal/mol
SER-46
HIS-41
S1'
ARG-188
S2
GLN-189
CYS-145
THR-190
S4
S1
GLN-192
S1
S4
S1'
Compound 4n|Cluster 2|7.187 kcal/mol
SER-46
HIS-41
S1'
S2
GLN-189
GLY-143
CYS-145
S1
S4
GLU-166
PHE-140
S4
S2
S1
S1'
Compound 4n|Cluster 3|6.976 kcal/mol
HIS-41
S2
S1'
GLY-143
CYS-145
ASN-142
S4
S1
S1'
S4
S1
Compound 2a|Cluster 2|6.274 kcal/mol
SER-46
HIS-41
S1'
S2
GLY-143
CYS-145
S4
S1
S4
S2
S1'
S1
Compound 2a|Cluster 3|5.886 kcal/mol
THR-26
HIS-41
S2
S1'
CYS-145
ASN-142
S4
SER-144
S1
HIS-163
GLU-166
LEU-167
PRO-168
GLY-170
S1'
S1

Compound 2y|Cluster 2|6.174 kcal/mol

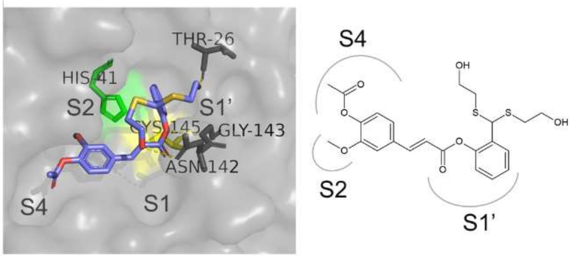

Compound 2y|Cluster 3|6.099 kcal/mol

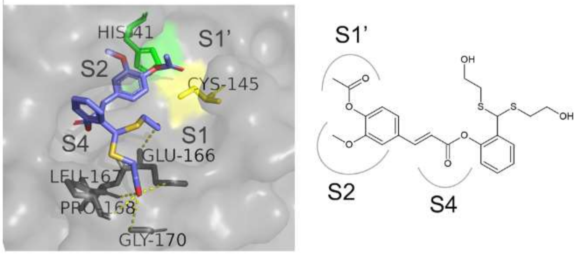

Compound 2s|Cluster 2|6.662 kcal/mol

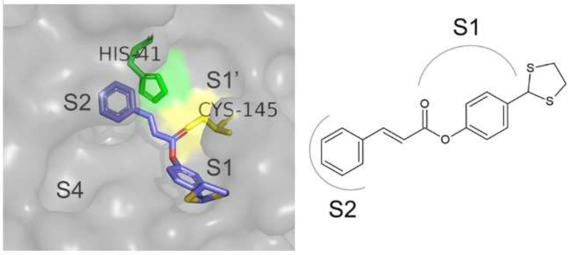

Compound 2s|Cluster 3|6.313 kcal/mol

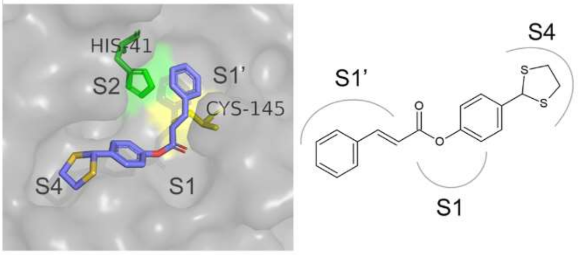

Compound F3|Cluster 2|7.648 kcal/mol

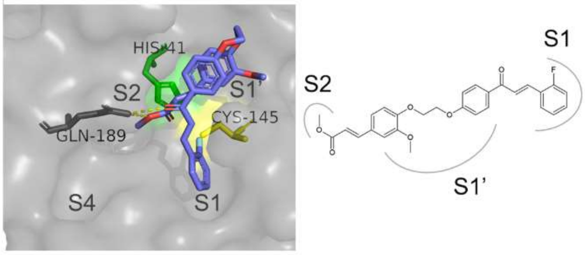

Compound F3|Cluster 3|7.604 kcal/mol

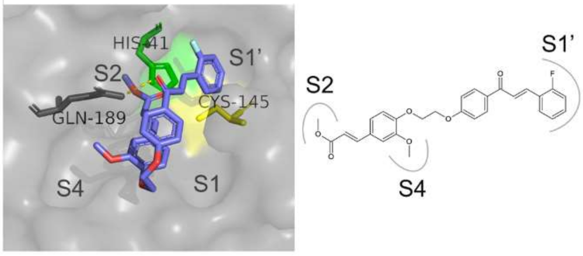

Compound D4|Cluster 2|6.607 kcal/mol

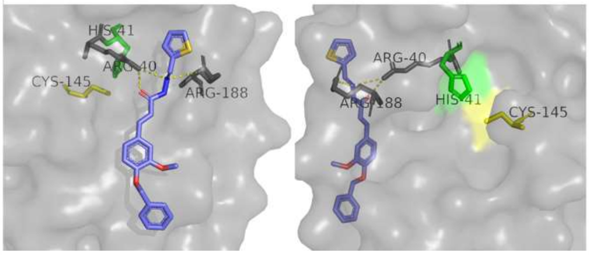

Compound D4|Cluster 3|6.509 kcal/mol

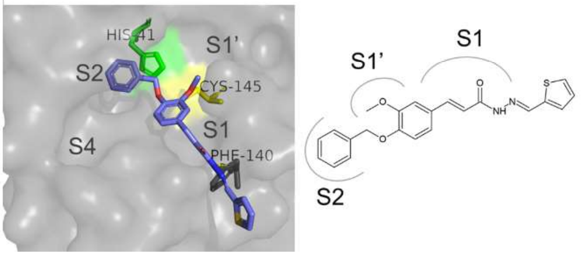

Ferulic acid dimer|Cluster 2|7.001 kcal/mol

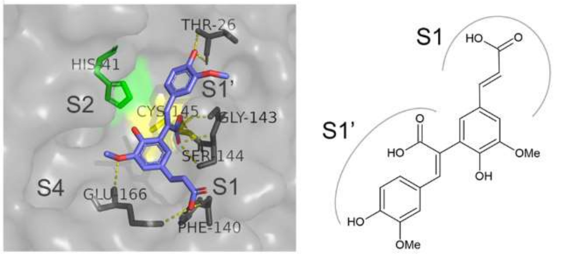

Ferulic acid dimer|Cluster 3|6.875 kcal/mol

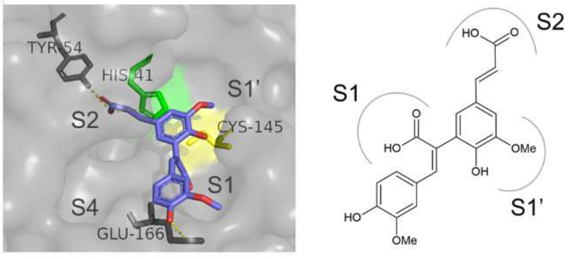

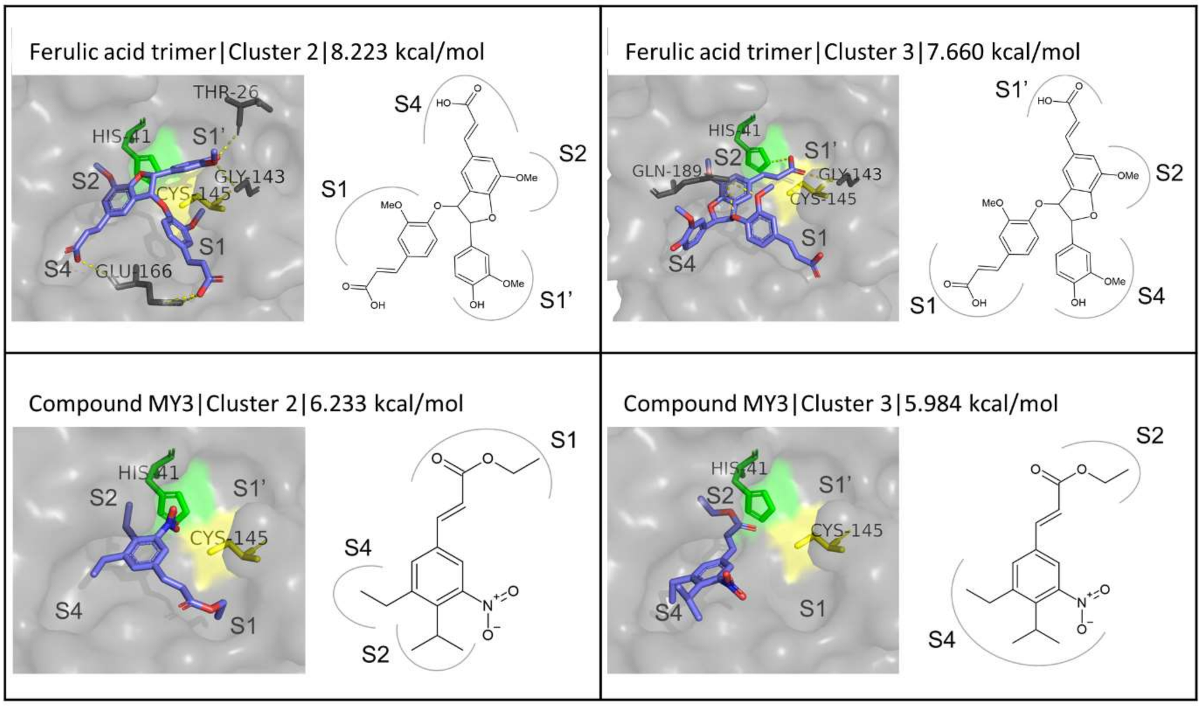

Figure S8. Binding modes of the second and third clusters for chemically synthesized FA derivatives in the active site of M^{pro}.
